# The Very First Modification of Pleuromutilin and Lefamulin by Photoinitiated Radical Addition Reactions—Synthesis and Antibacterial Studies

**DOI:** 10.3390/pharmaceutics13122028

**Published:** 2021-11-28

**Authors:** Son Thai Le, Dávid Páll, Erzsébet Rőth, Tuyen Tran, Nóra Debreczeni, Miklós Bege, Ilona Bereczki, Eszter Ostorházi, Márton Milánkovits, Pál Herczegh, Anikó Borbás, Magdolna Csávás

**Affiliations:** 1 Department of Pharmaceutical Chemistry, University of Debrecen, Egyetem tér 1, H-4032 Debrecen, Hungary; le.thai.son@pharm.unideb.hu (S.T.L.); Azgard-Gwendir@hotmail.com (D.P.); rothnej@gmail.com (E.R.); tuyentran1211@gmail.com (T.T.); debreczeni.nora@science.unideb.hu (N.D.); bege.miklos@euipar.unideb.hu (M.B.); bereczki.ilona@pharm.unideb.hu (I.B.); herczegh.pal@pharm.unideb.hu (P.H.); 2 Doctoral School of Pharmaceutical Sciences, University of Debrecen, Egyetem tér 1, H-4032 Debrecen, Hungary; 3 Doctoral School of Chemistry, University of Debrecen, Egyetem tér 1, H-4032 Debrecen, Hungary; 4 Institute of Healthcare Industry, University of Debrecen, Nagyerdei körút 98, H-4032 Debrecen, Hungary; 5 Loránd Eötvös Research Network, Molecular Recognition and Interaction Research Group, University of Debrecen, Egyetem tér 1, H-4032 Debrecen, Hungary; 6 Department of Medical Microbiology, Semmelweis University, Mária u. 41, H-1085 Budapest, Hungary; ostorhazi.eszter@med.semmelweis-univ.hu (E.O.); milankovits.marton@gmail.com (M.M.)

**Keywords:** pleuromutilin, lefamulin, synthesis, photoinitiated thiol-ene addition, atom transfer radical addition, perfluoroalkylated side chains, semisynthetic antibiotics, antibacterial effect, MRSA

## Abstract

Pleuromutilin is a fungal diterpene natural product with antimicrobial properties, semisynthetic derivatives of which are used in veterinary and human medicine. The development of bacterial resistance to pleuromutilins is known to be very slow, which makes the tricyclic diterpene skeleton of pleuromutilin a very attractive starting structure for the development of new antibiotic derivatives that are unlikely to induce resistance. Here, we report the very first synthetic modifications of pleuromutilin and lefamulin at alkene position C19–C20, by two different photoinduced addition reactions, the radical thiol-ene coupling reaction, and the atom transfer radical additions (ATRAs) of perfluoroalkyl iodides. Pleuromutilin were modified with the addition of several alkyl- and aryl-thiols, thiol-containing amino acids and nucleoside and carbohydrate thiols, as well as perfluoroalkylated side chains. The antibacterial properties of the novel semisynthetic pleuromutilin derivatives were investigated on a panel of bacterial strains, including susceptible and multiresistant pathogens and normal flora members. We have identified some novel semisynthetic pleuromutilin and lefamulin derivatives with promising antimicrobial properties.

## 1. Introduction

The development of novel semisynthetic antibiotics is essential for society, to protect human health from threatening antimicrobial infections and antibiotic resistance. It is estimated that by 2050, the number of deaths caused by antimicrobial resistant pathogens will be 10 million per year. The WHO have stated that “antibiotic resistance is putting the achievements of modern medicine at risk” [1]. This statement summarizes the difficulty of antibiotic developments in one sentence. Luckily, modern medicinal chemistry sometimes finds “forgotten” drugs for improvement; previously discovered drugs can find new therapeutic applications. Moreover, some synthetic modifications can enhance the antibacterial effect and/or break down antibiotic resistance.

**Figure 1:**
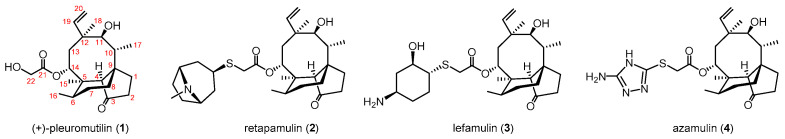
Pleuromutilin derivatives approved or studied for human antibacterial therapies.

Pleuromutilin (**1**) was discovered in the middle of the 20th century in species of *Clitopilus* fungi, a secondary metabolite diterpene with a tricyclic skeleton. It exerts antibiotic activity against Gram-positive bacteria by acting as a peptidyl tranferase inhibitor [2] (Figure 1). Semisynthetic derivatives [3,4,5,6,7,8,9,10], bearing a basic substituent at the C14 glycolic ester moiety of the parent pleuromutilin, have been developed for human and veterinary use. Valnemulin and tiamulin have long been used in veterinary medicine [11], retamapulin (**2**) was introduced in 2007 for the treatment of bacterial skin infections [12], and lefamulin (**3**) [13,14] has very recently been approved for systemic use to treat community-acquired pneumonia (CAP) in adults. Azamulin (**4**), an azole derivative of pleuromutilin was also intended for human use; however, it did not progress beyond a Phase I trial, because it proved to be a strong and irreversible inhibitor of CYP3A, the most abundant isozyme of cytochrome P450 enzymes. The C22 modifications of these compounds broadened the antibacterial spectrum to include drug-resistant Gram-negative bacteria [8].

Although there are three known resistance mechanisms against these drugs [8] (mutations in ribosomal protein L3, nucleotide methylation by Cfr methyltransferase and efflux), the development of all types of resistance is slow, which is one of the most valuable features of pleuromutilins [3]. Semisynthetic pleuromutilin derivatives is a subject of heightened interest, because of the lack of cross-resistance with other antibiotic classes [3]. In addition, the COVID-19 pandemic has caused a high incidence of infections in the bloodstream during hospitalization, which places an urgent need for the development of antibiotics that are effective against nosocomial infections [15].

The mechanism of action [3,6,16] of pleuromutilin and its derivatives is based on the inhibition of bacterial protein synthesis by binding to the 50S ribosomal subunit at the peptidyl transferase center, and finally inhibiting the peptide bond formation.

Several novel semisynthetic pleuromutilin-moieties are known from the literature, mainly obtained by modifications at the glycolic ester residue (position C-22) [8]. These modifications have shifted the antibacterial effect of these drugs to a broader spectrum, and the activities against Gram-negative bacterial strains were enhanced. Recently, novel pleuromutilin derivatives with substituted thiadiazole [17] and triazole moieties [18] at position C-22 have been published as promising antibacterial agents.

**Figure 2:**
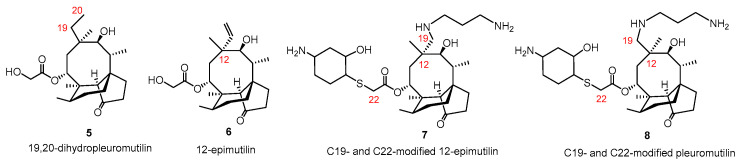
Structural modifications of pleuromutilin and C12-*epi*-mutilin at position C19-C20.

Some examples for modified pleuromutilin at position C-19 and C-20 are known, namely, 19,20-dihydropleuromutilin **5**, as well as chain elongated derivatives (e.g., **7**, **8**) of pleuromutilin and its epimeric compound 12-epimutilin (**6**, Figure 2) [19]. The antibacterial evaluation of these compounds revealed that saturation of the C19 double bond had no influence on the antimicrobial effect, indicating that the C19–C20 alkene is not essential for activity [8]. Moreover, while the introduction of a basic side chain at the C19-C20 position of 12-epimutilin provided derivatives (e.g., **7**) with activity against drug-resistant Gram-negative pathogens, the same modification of the parent pleuromutilin (**8**) led to a slight decrease in the antibacterial effect [18].

While synthetic modifications of pleuromutilin at the C22 position have extensively been studied leading to the discovery of several clinical and advanced preclinical agents, modifications at other positions, including the C19–C20 alkene moiety, are quite unexplored [8]. We decided to perform a systematic study to investigate how lipophilic, hydrophilic, and perfluorous substituents at position C20 modify the antibacterial activity of the parent compound. Considering the literature data, we focused on the introduction of side chains of a non-basic character. We assumed that radical addition reactions are ideally suited to the diverse functionalization of pleuromutilin at the C20 position under mild conditions, and the most successful derivatizations could be applied in the case of lefamulin.

The photoinduced radical-mediated addition of thiols to terminal alkenes could provide an easy and efficient method for the regioselective introduction of either hydrophilic or lipophilic groups at position C20. Our previous works demonstrated the wide range and useful application of hydrothiolation [20,21,22,23,24] in the field of carbohydrate, nucleoside, and antibiotic chemistry. Moreover, the light-promoted radical addition reaction [25] of commercially available perfluoroalkyl iodides onto the C19-C20 double bond allows the introduction of fluorous side chains into the pleuromutilin skeleton. Importantly, perfluoroalkylated derivatives of teicoplanin and vancomycin have recently been synthesized [26] by our group, and the introduced perfluorous side chains having a dual hydrophobic and lipophobic character [27] have remarkably improved the antimicrobial properties of the parent glycopeptide antibiotics.

It is important to note that, to the best of our knowledge, only one example can be found for the modification of pleuromutilin by radical addition reactions at alkene position C19–C20. Bacqué and et. al. have modified the pleuromutilin by thermally induced radical additions of various xanthates [28]. Unfortunately, the biological activity of these pleuromutilin derivatives has not been reported.

## 2. Materials and Methods

### 2.1. General Methods

Optical rotations were measured at room temperature, with a Perkin-Elmer 241 automatic polarimeter (Perkin-Elmer, Waltham, MA, USA). TLC analysis was performed on Kieselgel 60 F_254_ (Merck KGaA, Darmstadt, Germany) silica gel plates with visualization, by immersion in a sulfuric-acid solution (5% in EtOH) followed by heating. Flash column chromatography was performed on silica gel 60 (Merck KGaA, Darmstadt, Germany) 0.040–0.063 mm. The organic solutions were dried over Na_2_SO_4_ and concentrated under vacuum. The melting points were measured by a Büchi B-540 melting-point apparatus (Merck KGaA, Darmstadt, Germany). The ^1^H (360, 400 and 500 MHz) and ^13^C NMR (90, 100.28, 125.76 MHz) spectra were recorded with Bruker DRX-360, DRX-400 and Bruker Avance II 500 spectrometers. Chemical shifts are referenced to Me_4_Si or DSS (0.00 ppm for ^1^H) and to solvent signals (CDCl_3_: 77.00 ppm, CD_3_OD: 49.15 ppm, DMSO-*d*_6_: 39.52 ppm for ^13^C). NMR spectra of all compounds are given as Supplementary Information. ESI-QTOF MS measurements were carried out on a maXis II UHR ESI-QTOF MS instrument (Bruker, Billerica, MA, USA), in positive ionization mode. The following parameters were applied for the electrospray ion source: capillary voltage: 3.5 kV; end plate offset: 500 V; nebulizer pressure: 0.8 bar; dry gas temperature: 200 °C; dry gas flow rate: 4.5 L/min. Constant background correction was applied for each spectrum; the background was recorded before each sample by injecting the blank sample matrix (solvent). Na-formate calibrant was injected after each sample, which enabled internal calibration during data evaluation. Mass spectra were recorded by OTOF Control version 4.1 (build: 3.5, Bruker, Billerica, MA, USA) and processed by Compass DataAnalysis version 4.4 (build: 200.55.2969, Bruker, Billerica, MA, USA). MALDI-TOF MS measurements were carried out with a Bruker Autoflex Speed mass spectrometer, equipped with a time-of-flight (TOF) mass analyzer. In all cases, 19 kV (ion source voltage 1) and 16.65 kV (ion source voltage 2) were used. For reflectron mode, 21 kV and 9.55 kV were applied as reflector voltage 1 and reflector voltage 2, respectively. A solid-phase laser, (355 nm, ≥100 μJ/pulse) operating at 500 Hz, was applied to produce laser desorption, and 3000 shots were summed. Additionally, 2,5-Dihydroxybenzoic acid (DHB) was used as matrix, and F_3_CCOONa as the cationising agent in DMF.

The photoinitiated reactions were carried out in a borosilicate vessel by irradiation, with an Hg-lamp giving maximum emission at 365 nm (See Figure S28).

For the in vitro MIC measurements, we used 12 different Gram-positive bacterial strains. Some of these strains were purchased from the American Type Culture Collection (ATCC), whereas others were clinical isolates. Our bacterial collection contained wild-typed-sensitive and also multiresistant strains. According to the EUCAST (European Committee on Antimicrobial Susceptibility Testing) reading guide for broth microdilution [29], compounds were two-fold serially diluted from 256 to 0.5 mg/L in Müller–Hinton broth. Then, 100 µL of each dilution was inoculated with 10 µL of 0.5 McFarland bacterial suspension. Incubation was performed at 37 °C for 24 h without shaking, and determination of MIC was made with the naked eye.

### 2.2. General Method for Photoinitiated Thiol-Ene Addition

To a solution of pleuromutilin (0.50 mmol) in the specified solvent (5 mL), thiol (1-2 equiv.) and 2,2-dimethoxy-2-phenylacetophenone (DPAP, 12 mg, 0.050 mmol) were added. The solution was irradiated with UV light at room temperature, or under cooling, for 1–3 × 15 min (the addition of 0.1 equiv. of DPAP and thiol (1-2 equiv.) were repeated before each irradiation cycle) at the given temperature. Then, the mixture was concentrated, and the residue was purified using column chromatography.

### 2.3. Synthesis

#### 2.3.1. Compound **10a**

Pleuromutilin (95 mg, 0.25 mmol) and thiol **9a** (153 mg, 0.5 mol, 2 × 1 equiv.) were reacted in CH_3_CN at room temperature according to the general method, using two irradiating cycles. The crude product was purified by flash column chromatography (CH_2_Cl_2_/acetone 99/1) to result in compound **10a** as white powder (140 mg, 81%). R_f_: 0.31 (CH_2_Cl_2_/acetone 9/1), [α]^24^_D_ +43.0 (*c* 0.1, MeOH), m.p. 192–193 °C. ^1^H NMR (400 MHz, Chloroform-*d*) δ 5.62 (d, *J* = 8.2 Hz, 1H), 5.24 − 5.20 (m, 1H), 5.12 (t, *J* = 9.9 Hz, 1H), 5.03 (dd, *J* = 10.0, 3.3 Hz, 1H), 4.55 (d, *J* = 9.7 Hz, 1H), 4.12 − 3.97 (m, 2H), 3.96 − 3.86 (m, 1H), 3.38 (d, *J* = 5.7 Hz, 1H), 3.20 (s, 1H), 2.60 (dt, *J* = 11.9, 6.0 Hz, 1H), 2.45 (td, *J* = 12.0, 5.0 Hz, 1H), 2.30 (t, *J* = 6.7 Hz, 1H), 2.11 (d, *J* = 1.4 Hz, 9H), 2.03 (s, 4H), 1.92 (s, 3H), 1.86 − 1.67 (m, 2H), 1.65 − 1.39 (m, 3H), 1.35 (s, 4H), 1.21 (d, *J* = 5.2 Hz, 1H), 1.16 (d, *J* = 6.4 Hz, 3H), 1.14 − 1.01 (m, 1H), 0.99 (s, 3H), 0.90 (d, *J* = 7.0 Hz, 3H), 0.63 (d, *J* = 6.9 Hz, 3H). ^13^C NMR (101 MHz, Chloroform-*d*) δ 216.9, 172.2, 170.6, 170.2, 170.1, 82.9, 75.9, 72.8, 72.3, 70.6, 69.5, 67.7, 61.3, 58.2, 45.5, 42.0, 41.8, 41.2, 36.5, 34.5, 34.4, 30.9, 30.1, 29.6, 26.8, 26.6, 24.9, 24.8, 20.9, 20.6, 16.5, 16.4, 14.7, 11. HRMS (ESI): *m*/*z* calcd. for C_34_H_52_NaO_12_S: 707.3077 [M+Na]^+^; found: 707.3070.

#### 2.3.2. Compound **10b**

Pleuromutilin (125 mg, 0.33 mmol) and thiol **9b** (119 mg, 0.66 mmol, 2 × 1 equiv.) were reacted in EtOH at room temperature according to the general method, using two irradiating cycles. The crude product was purified by flash column chromatography (CH_2_Cl_2_/MeOH 95/5) to result in compound **10b** as white powder (121 mg, 66%). R_f_: 0.31 (CH_2_Cl_2_/MeOH 9/1), [α]^24^_D_ +12.22 (*c* 0.09, MeOH), m.p. 91–93 °C. ^1^H NMR (400 MHz, Methanol-*d*_4_) δ 5.78 (d, *J* = 8.2 Hz, 1H), 4.57 − 4.43 (m, 1H), 4.14 (d, *J* = 17.3 Hz, 1H), 4.02 (d, *J* = 17.1 Hz, 1H), 3.88 − 3.77 (m, 1H), 3.69 (d, *J* = 3.2 Hz, 1H), 3.62 − 3.51 (m, 2H), 3.47 (d, *J* = 5.8 Hz, 1H), 3.33 (t, *J* = 1.7 Hz, 1H), 2.65 (td, *J* = 12.3, 4.2 Hz, 1H), 2.52 (td, *J* = 12.6, 4.9 Hz, 1H), 2.38 (dd, *J* = 14.6, 4.6 Hz, 2H), 2.33 − 2.23 (m, 1H), 2.19 (t, *J* = 9.4 Hz, 2H), 2.10 (ddd, *J* = 17.7, 11.4, 5.3 Hz, 2H), 2.00 − 1.80 (m, 4H), 1.77 − 1.54 (m, 4H), 1.45 (s, 3H), 1.38 (d, *J* = 21.9 Hz, 1H), 1.30 (t, *J* = 5.9 Hz, 4H), 1.23 − 1.11 (m, 1H), 1.04 (d, *J* = 2.9 Hz, 3H), 0.97 (d, *J* = 6.9 Hz, 3H), 0.74 (d, *J* = 6.1 Hz, 3H). ^13^C NMR (101 MHz, Methanol-*d*_4_) δ 217.0 172.5, 85.3, 75.1, 74.9, 74.3, 71.9, 69.6, 69.0, 60.5, 57.9, 45.4, 41.7, 41.4, 41.0, 36.7, 34.8, 33.9, 30.1, 30.0, 26.7, 25.9, 24.7, 24.3, 15.8, 15.6, 13.9, 10.4. HRMS (ESI): *m*/*z* calcd for C_28_H_46_NaO_9_S: 581.2760 [M + Na]^+^; found: 581.2754.

#### 2.3.3. Compound **10c**

The reaction was carried out using the general method, starting from pleuromutilin (190 mg, 0.5 mmol) and thiol **9c** (365 mg, 1.0 mmol, 2 × 1 equiv.) in CH_3_CN at room temperature, irradiating two times. The crude product was purified by flash column chromatography (CH_2_Cl_2_/acetone 95/5) to result in compound **10c** as white powder (219 mg, 59%). R_f_: 0.38 (CH_2_Cl_2_/acetone 9/1), [α]^24^_D_ +107.0 (*c* 0.1, CHCl_3_), m.p. 127–130 °C. ^1^H NMR (400 MHz, Methanol-*d*_4_) δ 5.71 (d, *J* = 8.3 Hz, 1H), 5.48 (d, *J* = 1.5 Hz, 1H), 5.36 (dd, *J* = 3.2, 1.5 Hz, 1H), 5.31 (t, *J* = 9.9 Hz, 1H), 5.25 (dd, *J* = 10.1, 3.3 Hz, 1H), 4.46 (ddd, *J* = 9.6, 4.8, 2.4 Hz, 1H), 4.31 (dd, *J* = 12.3, 4.8 Hz, 1H), 4.15 (dd, *J* = 12.3, 2.5 Hz, 1H), 4.11 − 3.97 (m, 2H), 3.48 (d, *J* = 5.8 Hz, 1H), 2.72 − 2.48 (m, 2H), 2.36 (d, *J* = 7.1 Hz, 2H), 2.31 − 2.19 (m, 1H), 2.18 (d, *J* = 1.6 Hz, 5H), 2.08 (d, *J* = 5.8 Hz, 6H), 2.03 (dd, *J* = 9.9, 7.3 Hz, 2H), 1.99 (s, 1H), 1.97 − 1.79 (m, 4H), 1.76 − 1.54 (m, 4H), 1.45 (s, 3H), 1.30 (dd, *J* = 16.0, 6.9 Hz, 2H), 1.23 − 1.11 (m, 1H), 1.05 (s, 3H), 0.97 (d, *J* = 7.0 Hz, 3H), 0.75 (d, *J* = 6.3 Hz, 3H). ^13^C NMR (101 MHz, Methanol-*d*_4_) δ 217.0, 172.1, 171.0, 170.3, 170.2, 170.1, 81.5, 74.9, 70.9, 69.8, 69.0, 68.7, 66.1, 62.4, 60.6, 57.8, 45.4, 41.8, 41.7, 41.0, 36.6, 34.8, 33.9, 30.0, 29.7, 26.7, 26.0, 25.9, 24.3, 19.4, 19.4, 19.3, 19.2, 15.8, 13.9, 10.4. HRMS (ESI): *m*/*z* calcd. for C_36_H_54_NaO_14_S: 765.3132 [M + Na]^+^; found: 765.3125.

#### 2.3.4. Compound **10d**

The reaction was carried out using the general method, starting from pleuromutilin (190 mg, 0.5 mmol) and thiol **9d** (364 mg, 1.0 mmol, 2 × 1 equiv.) in CH_3_CN at room temperature, irradiating two times. The crude product was purified by flash column chromatography (CH_2_Cl_2_/acetone 95/5) to result in compound **10d** as white powder (255 mg, 69%). R_f_: 0.11 (CH_2_Cl_2_/acetone 9/1), [α]^24^_D_ +13.0 (*c* 0.1, CHCl_3_), m.p. 97–98 °C. ^1^H NMR (400 MHz, Chloroform-*d*) δ 6.79 (d, *J* = 9.3 Hz, 1H), 5.70 (d, *J* = 8.2 Hz, 1H), 5.41 − 5.24 (m, 1H), 5.13 (t, *J* = 9.7 Hz, 1H), 4.68 (d, *J* = 10.4 Hz, 1H), 4.25 (dd, *J* = 12.3, 4.7 Hz, 1H), 4.19 − 4.10 (m, 3H), 4.10 − 3.97 (m, 1H), 3.76 (ddd, *J* = 9.9, 4.6, 2.4 Hz, 1H), 3.42 (d, *J* = 5.7 Hz, 1H), 2.80 (td, *J* = 12.3, 4.3 Hz, 1H), 2.37 (tq, *J* = 12.8, 7.8, 6.2 Hz, 3H), 2.29 − 2.21 (m, 1H), 2.18 (s, 1H), 2.14 − 2.06 (m, 4H), 2.08 − 1.95 (m, 10H), 1.95 − 1.74 (m, 3H), 1.73 − 1.52 (m, 2H), 1.53 − 1.44 (m, 2H), 1.42 (s, 4H), 1.33 − 1.20 (m, 2H), 1.14 (td, *J* = 14.0, 4.5 Hz, 1H), 1.03 (s, 3H), 0.96 (d, *J* = 6.9 Hz, 3H), 0.70 (d, *J* = 7.0 Hz, 3H). ^13^C NMR (101 MHz, Chloroform-*d*) δ 217.0, 172.7, 171.1, 171.1, 170.9, 169.4, 86.1, 76.0, 75.8, 73.8, 69.6, 68.4, 62.5, 61.4, 58.2, 53.5, 45.5, 42.3, 41.8, 41.3, 36.5, 34.7, 34.4, 30.8, 30.2, 27.7, 26.9, 26.7, 24.8, 23.2, 20.8, 20.8, 20.7, 16.6, 14.7, 11.1. HRMS (ESI): *m*/*z* calcd. for C_36_H_55_NNaO_13_S: 764.3292 [M + Na]^+^; found: 764.3287.

#### 2.3.5. Compound **10e**

The reaction was carried out using the general method, starting from pleuromutilin (190 mg, 0.5 mmol) and thiol **9e** (142 mg, 0.6 mmol, 1.2 equiv.) in CH_3_CN/MeOH 4/1 at room temperature, irradiating once. The crude product was purified by flash column chromatography (CH_2_Cl_2_/MeOH 8/2) to result in compound **10e** as white amorphous solid (290 mg, 94%). R_f_: 0.42 (CH_2_Cl_2_/MeOH 8/2), [α]^24^_D_ +3.85 (c 0.13, CHCl_3_). ^1^H NMR (400 MHz, DMSO-*d*_6_) δ 7.74 (d, *J* = 9.4 Hz, 1H), 5.55 (d, *J* = 8.2 Hz, 1H), 5.39 (t, *J* = 6.6 Hz, 1H), 5.12 − 4.86 (m, 2H), 4.56 (d, *J* = 6.0 Hz, 1H), 4.50 (t, *J* = 6.0 Hz, 1H), 4.37 (d, *J* = 10.3 Hz, 1H), 4.21 − 4.11 (m, 1H), 4.03 − 3.79 (m, 2H), 3.75 − 3.63 (m, 1H), 3.22 − 3.05 (m, 3H), 2.64 − 2.48 (m, 2H), 2.46 − 2.30 (m, 2H), 2.18 (td, *J* = 12.7, 11.2, 6.8 Hz, 2H), 2.06 (dt, *J* = 18.7, 9.2 Hz, 1H), 1.94 − 1.77 (m, 5H), 1.67 (ttd, *J* = 22.0, 11.7, 7.8 Hz, 3H), 1.55 − 1.39 (m, 2H), 1.38 − 1.18 (m, 6H), 1.18 − 0.95 (m, 2H), 0.92 (s, 3H), 0.84 (d, *J* = 6.9 Hz, 3H), 0.62 (d, *J* = 6.2 Hz, 3H). ^13^C NMR (101 MHz, DMSO-*d*_6_) δ 217.7, 172.1, 169.6, 86.0, 81.2, 75.9, 74.1, 70.9, 68.3, 61.8, 60.8, 57.7, 55.2, 49.0, 45.4, 42.3, 41.8, 41.2, 36.8, 35.1, 34.4, 31.1, 30.6, 27.3, 26.7, 24.8, 23.5, 16.7, 15.0, 12.0. HRMS (ESI): *m*/*z* calcd for C_30_H_49_NNaO_10_S: 638.2975 [M + Na]^+^; found: 638.2969.

#### 2.3.6. Compound **10f**

The reaction was carried out using the general method, starting from pleuromutilin (190 mg, 0.5 mmol) and thiol **9f** (118 mg, 0.6 mmol, 1.2 equiv.) in MeOH at room temperature, irradiating once. The crude product was purified by flash column chromatography (CH_2_Cl_2_/MeOH 8/2) and resulted in compound **10f** as white amorphous solid (216 mg, 75%). R_f_: 0.71 (CH_2_Cl_2_/MeOH 7/3), [α]^24^_D_= +17.8 (c 0.09, CHCl_3_). ^1^H NMR (400 MHz, DMSO-*d*_6_) δ 5.75 (d, *J* = 2.7 Hz, 1H), 5.55 (d, *J* = 8.1 Hz, 1H), 5.33 (s, 1H), 4.98 (d, *J* = 40.6 Hz, 2H), 4.59 (d, *J* = 5.7 Hz, 1H), 4.46 (s, 1H), 4.27 (dd, *J* = 9.6, 2.7 Hz, 1H), 3.90 (q, *J* = 17.0 Hz, 2H), 3.67 (d, *J* = 11.8 Hz, 1H), 3.15 (dd, *J* = 10.1, 6.6 Hz, 2H), 3.08 (d, *J* = 9.2 Hz, 1H), 2.96 (t, *J* = 9.3 Hz, 1H), 2.63 − 2.39 (m, 5H), 2.35 (s, 1H), 2.18 (t, *J* = 8.4 Hz, 2H), 2.15 − 2.00 (m, 1H), 2.00 − 1.79 (m, 2H), 1.80 − 1.56 (m, 2H), 1.58 − 1.40 (m, 2H), 1.38 − 1.20 (m, 6H), 1.17 − 0.97 (m, 1H), 0.94 (d, *J* = 2.7 Hz, 3H), 0.85 (d, *J* = 6.8 Hz, 3H), 0.63 (d, *J* = 6.2 Hz, 3H). ^13^C NMR (101 MHz, DMSO-*d*_6_) δ 217.6, 172.0, 86.3, 81.1, 78.6, 74.0, 73.8, 70.5, 68.3, 61.8, 60.9, 57.7, 45.4, 42.6, 41.8, 41.2, 36.8, 35.1, 34.4, 31.6, 30.6, 27.3, 27.1, 25.9, 24.8, 16.7, 15.0, 12.0. HRMS (ESI): *m*/*z* calcd. for C_28_H_46_NaO_10_S: 597.2709 [M + Na]^+^; found: 597.2704.

#### 2.3.7. Compound **10g**

The reaction was carried out using the general method, starting from pleuromutilin (95 mg, 0.25 mmol) and thiol **9g** (109 mg, 0.3 mmol, 1.2 equiv.) in EtOAc at room temperature, irradiating once. The crude product was purified by flash column chromatography (CH_2_Cl_2_/acetone 95/5) to result in compound **10g** as white amorphous solid (114 mg, 61%). R_f_: 0.33 (CH_2_Cl_2_/acetone 9/1), [α]^24^_D_ +19.3 (c 0.14, CHCl_3_). ^1^H NMR (360 MHz, Chloroform-*d*) δ 5.66 (d, *J* = 8.3 Hz, 1H), 5.43 (d, *J* = 3.3 Hz, 1H), 5.36 − 5.18 (m, 3H), 5.12 (dd, *J* = 10.0, 3.3 Hz, 1H), 4.66 (d, *J* = 9.9 Hz, 1H), 4.16 − 3.97 (m, 4H), 3.42 (d, *J* = 5.6 Hz, 1H), 2.81 (t, *J* = 5.7 Hz, 1H), 2.57 (t, *J* = 8.5 Hz, 2H), 2.40 − 2.11 (m, 6H), 2.05 (s, 4H), 1.99 (s, 3H), 1.86 (ddd, *J* = 32.2, 16.3, 8.6 Hz, 5H), 1.70 − 1.43 (m, 6H), 1.42 (s, 3H), 1.32 − 1.22 (m, 1H), 1.21 − 1.02 (m, 5H), 0.97 (d, *J* = 6.9 Hz, 3H), 0.70 (d, *J* = 6.8 Hz, 3H). ^13^C NMR (91 MHz, Chloroform-*d*) δ 216.6, 172.3, 170.6, 170.2, 169.8, 84.1, 76.1, 74.0, 71.9, 69.7, 67.4, 67.3, 61.4, 61.3, 58.1, 45.4, 41.9, 41.8, 41.3, 36.4, 34.4, 34.3, 30.5, 30.0, 26.8, 25.5, 24.8, 20.9, 20.6, 16.6, 14.7, 10.9. HRMS (ESI): *m*/*z* calcd. for C_36_H_54_NaO_14_S: 765.3132 [M + Na]^+^; found: 765.3126.

#### 2.3.8. Compound **10h**

The reaction was carried out using the general method, starting from pleuromutilin (48 mg, 0.125 mmol) and thiol **9h** (191 mg, 0.375 mmol, 3 × 1 equiv.) in toluene/MeOH 1/1 at −40 °C, irradiating three times. The crude product was purified by flash column chromatography (CH_2_Cl_2_/acetone 9/1) to result in compound **10h** as white amorphous solid (69 mg, 62%). R_f_: 0.20 (CH_2_Cl_2_/acetone 8/2), [α]^24^_D_ +46.7 (c 0.12, MeOH). ^1^H NMR (400 MHz, Chloroform-*d*) δ 5.59 (d, *J* = 8.1 Hz, 1H), 5.46 (d, *J* = 10.1 Hz, 1H), 5.31 (q, *J* = 4.9, 3.6 Hz, 2H), 4.95 − 4.76 (m, 1H), 4.37 − 4.22 (m, 1H), 4.16 − 3.94 (m, 4H), 3.94 − 3.66 (m, 4H), 3.38 (d, *J* = 5.5 Hz, 1H), 2.71 (dd, *J* = 12.7, 4.6 Hz, 1H), 2.62 (s, 1H), 2.57 (dd, *J* = 12.2, 4.1 Hz, 1H), 2.43 (td, *J* = 11.7, 5.2 Hz, 1H), 2.30 (dt, *J* = 10.0, 4.8 Hz, 1H), 2.22 − 2.06 (m, 6H), 2.02 (d, *J* = 1.8 Hz, 8H), 1.86 (s, 4H), 1.75 (dt, *J* = 9.8, 5.8 Hz, 2H), 1.68 − 1.50 (m, 2H), 1.50 − 1.42 (m, 1H), 1.41 (s, 4H), 1.38 − 1.29 (m, 1H), 1.24 (s, 5H), 1.10 (td, *J* = 14.1, 3.0 Hz, 1H), 1.03 (s, 3H), 0.91 (d, *J* = 6.8 Hz, 3H), 0.67 (d, *J* = 6.9 Hz, 3H). ^13^C NMR (101 MHz, Chloroform-*d*) δ 218.0, 172.4, 170.9, 170.8, 170.3, 170.2, 168.4, 83.3, 76.0, 73.9, 69.8, 69.7, 68.3, 67.2, 62.4, 61.3, 58.3, 53.8, 52.9, 49.3, 45.4, 41.7, 41.7, 41.4, 38.0, 36.5, 34.4, 30.1, 29.6, 29.3, 28.0, 26.8, 26.1, 24.8, 24.5, 23.1, 21.2, 20.9, 20.8, 16.6, 14.8, 11.0. HRMS (ESI): *m*/*z* calcd. for C_42_H_63_NNaO_17_S: 908.3714 [M+Na]^+^; found: 908.3709.

#### 2.3.9. Compound **10i**

The reaction was carried out using the general method, starting from pleuromutilin (190 mg, 0.5 mmol) and thiol **9i** (390 mg, 1.5 mmol, 3 × 1 equiv.) in MeOH/DMF 9/1 at −80 °C, irradiating three times. The crude product was purified by flash column chromatography (CH_2_Cl_2_/MeOH 95/5 to 9:1) to result in compound **10i** as white amorphous solid (204 mg, 64%). R_f_: 0.51 (CH_2_Cl_2_/MeOH 8/2), [α]^24^_D_ +14.0 (*c* 0.1, MeOH). ^1^H NMR (400 MHz, Methanol-*d*_4_) δ 7.80 (d, *J* = 8.0 Hz, 1H), 5.88 (d, *J* = 4.8 Hz, 1H), 5.74 (t, *J* = 8.1 Hz, 2H), 4.23 (t, *J* = 4.7 Hz, 1H), 4.13 (q, *J* = 5.3, 4.6 Hz, 3H), 4.07 (s, 1H), 4.02 (s, 1H), 3.44 (d, *J* = 5.8 Hz, 1H), 3.35 (s, 1H), 3.31 (p, *J* = 1.6 Hz, 1H), 3.01 − 2.90 (m, 2H), 2.59 (td, *J* = 11.9, 4.9 Hz, 1H), 2.46 (td, *J* = 12.1, 5.4 Hz, 1H), 2.34 (qd, *J* = 11.6, 9.9, 4.3 Hz, 3H), 2.25 (dd, *J* = 10.3, 7.9 Hz, 1H), 2.14 (dt, *J* = 19.1, 9.3 Hz, 1H), 2.02 − 1.78 (m, 4H), 1.72 − 1.52 (m, 3H), 1.48 − 1.32 (m, 5H), 1.26 (d, *J* = 16.3 Hz, 2H), 1.20 − 1.09 (m, 1H), 1.01 (s, 3H), 0.95 (d, *J* = 6.9 Hz, 3H), 0.72 (d, *J* = 6.2 Hz, 3H). ^13^C NMR (101 MHz, Methanol-*d*_4_) δ 173.5, 166.1, 152.3, 142.7, 103.0, 90.9, 85.0, 76.3, 75.0, 73.4, 70.3, 61.9, 59.3, 48.9, 46.8, 43.3, 43.1, 42.3, 38.0, 36.2, 35.3, 35.1, 31.8, 31.4, 29.4, 28.1, 27.4, 25.7, 17.0, 15.3, 11.8. HRMS (ESI): *m*/*z* calcd. for C_31_H_46_N_2_NaO_10_S: 661.2873 [M + Na]^+^; found: 661.2765.

#### 2.3.10. Compound **10j**

The reaction was carried out using the general method, starting from pleuromutilin (95 mg, 0.25 mmol) and thiol **9j** (39 mg, 35µL, 0.5 mmol, 2 equiv.) in CH_3_CN/MeOH 2/1 at −40 °C, irradiating once. The crude product was purified by flash column chromatography (*n*-hexane/acetone 8/2) to result in compound **10j** as white powder (89 mg, 78%). R_f_: 0.10 (*n*-hexane/acetone 7/3), [α]^24^_D_ +10.3 (*c* 0.35, MeOH), m.p. 189 °C. ^1^H NMR (400 MHz, Methanol-*d*_4_) *δ* 5.75 (d, *J* = 8.3 Hz, 1H, H-14), 4.03 (q, *J* = 17.1 Hz, 2H, H-22ab), 3.67 (t, *J* = 6.9 Hz, 2H, H-24ab), 3.44 (d, *J* = 5.9 Hz, 1H, H-11), 2.74 (dt, *J* = 13.8, 6.9 Hz, 1H, H-23a), 2.66 (dt, *J* = 13.6, 6.9 Hz, 1H, H-23b), 2.48 (td, *J* = 12.2, 4.7 Hz, 1H, H-19a*), 2.44 − 2.34 (m, 2H, H-10, H-19b*), 2.33 (s, 1H, H-4), 2.26 (dd, *J* = 19.2, 10.7 Hz, 1H, H-2a), 2.20 − 2.08 (m, 1H, H-2b), 2.03 − 1.93 (m, 1H, H-20a*), 1.92 − 1.80 (m, 3H, H-8a, H-13a, H-20b*), 1.73 − 1.65 (m, 1H, H-1a), 1.64 − 1.53 (m, 2H, H-6, H-7a), 1.45 (dd, *J* = 8.3, 6.2 Hz, 1H, H-1b), 1.42 (s, 3H, H-15abc), 1.37 (d, *J* = 10.8 Hz, 1H, H-7b), 1.32 − 1.25 (m, 1H, H-13b), 1.20 − 1.10 (m, 1H, H-8b), 1.01 (s, 3H, H-18abc), 0.95 (d, *J* = 7.0 Hz, 3H, H-17abc), 0.72 (d, *J* = 6.1 Hz, 3H, H-16abc). * H-19a↔H-20a and H-19b↔H-20b are interchangeable. ^13^C NMR (101 MHz, Methanol-*d*_4_) *δ* 219.6 (1C, C-3), 173.5 (1C, C-21), 76.4 (1C, C-11), 70.3 (1C, C-14), 62.8, 61.9 (2C, C-22, C-24), 59.3 (1C, C-4), 46.8 (1C, C-9), 43.1 (1C, C-13), 43.1 (1C, C-12), 42.2 (1C, C-5), 38.1 (1C, C-6), 36.2 (1C, C-10), 35.3, 34.9 (2C, C-2, C-23), 31.6, 31.4, 28.2, 28.1 (4C, C-7, C-19, C-8, C-20), 27.4 (1C, C-18), 25.7 (1C, C-1), 17.0 (1C, C-16), 15.3 (1C, C-15), 11.8 (1C, C-17). HRMS (ESI): *m*/*z* calcd. for C_20_H_40_NaO_6_S: 479.2443 [M + Na]^+^; found: 479.2438.

#### 2.3.11. Compound **10k**

The reaction was carried out using the general method, starting from pleuromutilin (95 mg, 0.25 mmol) and thiol **9k** (82 mg, 0.5 mmol, 2 × 1 equiv.) in MeOH at −80 °C, irradiating two times. The crude product was purified by flash column chromatography (CH_2_Cl_2_/MeOH 9/1) to result in compound **10k** as white-yellow powder (125 mg, 92%). R_f_: 0.31 (CH_2_Cl_2_/MeOH 7/3), [α]^24^_D_ +34.3 (*c* 0.14, MeOH), m.p. 73–74 °C. ^1^H NMR (400 MHz, DMSO-*d*_6_) *δ* 7.85 (d, *J* = 6.2 Hz, 1H, N*H*), 5.56 (d, *J* = 7.8 Hz, 1H, H-14), 4.22 (s, 1H, H-24), 4.01 (d, *J* = 17.1 Hz, 1H, H-22a), 3.82 (d, *J* = 17.1 Hz, 1H, H-22b), 3.34 (d, *J* = 5.1 Hz, 1H, H-11), 2.91 (d, *J* = 9.7 Hz, 1H, H-23a), 2.74 (dd, *J* = 12.4, 5.8 Hz, 1H, H-23b), 2.46 − 2.25 (m, 3H, H-4, H-19ab*), 2.18 (t, *J* = 14.3 Hz, 2H, H-10, H-2a), 2.11 − 1.99 (m, 1H, H-2b), 1.86 (s, 3H, AcC*H*_3_), 1.81 (d, *J* = 8.0 Hz, 1H, H-13a), 1.64 (dd, *J* = 29.7, 9.9 Hz, 4H, H-8a, H-7a, H-20ab*), 1.44 (dd, *J* = 23.1, 9.1 Hz, 2H, H-6, H-1a), 1.32 (s, 3H, H-15abc), 1.29 − 1.22 (m, 2H, H-1b, H-7b), 1.09 (d, *J* = 16.0 Hz, 1H, H-13b), 1.00 (d, *J* = 12.7 Hz, 1H, H-8b), 0.91 (s, 3H, H-18abc), 0.82 (d, *J* = 6.6 Hz, 3H, H-17abc), 0.61 (d, *J* = 5.9 Hz, 3H, H-16abc). *H-19a↔H-20a, H-19b↔H-20b and C19↔20 are interchangeable signals. ^13^C NMR (101 MHz, DMSO-*d*_6_) *δ* 217.2 (1C, C-3), 172.3, 168.9 (3C, Ac*C*O, *C*OOH, C-21), 73.6 (1C, C-11), 67.7 (1C, C-14), 60.4 (1C, C-22), 57.3 (1C, C-4), 53.3 (1C, C-24), 55.0, 45.0, 41.4, 40.7 (4C, C-5, C-12, C-13, C-9), 36.4 (1C, C-6), 34.7 (1C, C-10), 34.5, 34.0 (2C, C-23, C-2), 30.2, 29.7 (2C, C-8, C-20*), 27.6 (1C, C-19*), 26.9 (1C, C-18), 26.7 (1C, C-1), 24.4 (1C, C-7), 22.8 (1C, Ac*C*H_3_), 16.2 (1C, C-16), 14.56 (1C, C-15), 11.6 (1C, C-17). HRMS (MALDI): *m*/*z* calcd. for C_27_H_43_NNaO_8_S: 564.2607 [M + Na]^+^; found: 564.2616.

#### 2.3.12. Compound **10l**

The reaction was carried out using the general method, starting from pleuromutilin (95 mg, 0.25 mmol) and thiol **9l** (172 mg, 0.5 mmol, 2 × 1 equiv.) in toluene/MeOH 1/1 at −40 °C, irradiating two times. The crude product was purified by flash column chromatography (toluene/MeOH 95/5) to result in compound **10l** as yellow powder (169 mg, 93%). R_f_: 0.11 (toluene/MeOH 8/2), [α]^24^_D_ +15.3 (*c* 0.15, MeOH), m.p. 143–147 °C. ^1^H NMR (500 MHz, DMSO-*d*_6_) δ 7.89 (d, *J* = 7.6 Hz, 2H), 7.74 (t, *J* = 6.9 Hz, 2H), 7.42 (t, *J* = 7.5 Hz, 2H), 7.34 (t, *J* = 7.5 Hz, 2H), 5.60 (d, *J* = 8.0 Hz, 1H), 4.32 − 4.23 (m, 2H), 4.22 (q, *J* = 6.7, 6.1 Hz, 2H), 4.06 (d, *J* = 17.1 Hz, 1H), 3.82 (d, *J* = 17.1 Hz, 1H), 3.00 (dd, *J* = 13.9, 4.5 Hz, 1H), 2.79 (dd, *J* = 13.5, 6.8 Hz, 1H), 2.37 (d, *J* = 21.5 Hz, 3H), 2.24 − 2.14 (m, 2H), 2.12 − 1.96 (m, 1H), 1.83 (dd, *J* = 16.2, 8.0 Hz, 1H), 1.67 (ddt, *J* = 37.8, 21.7, 10.0 Hz, 4H), 1.49 (d, *J* = 7.9 Hz, 2H), 1.40 − 1.28 (m, 5H), 1.30 − 1.21 (m, 5H), 1.11 (d, *J* = 15.8 Hz, 2H), 0.92 (s, 3H), 0.89 − 0.80 (m, 3H), 0.63 (d, *J* = 5.7 Hz, 3H). ^13^C NMR (126 MHz, DMSO-*d*_6_) δ 217.2, 172.20, 155.5, 143.9, 140.7, 127.6, 127.1, 125.4, 125.3, 120.1, 73.6, 67.5, 65.6, 60.3, 57.3, 46.7, 45.0, 41.3, 40.6, 36.3, 34.6, 33.9, 31.1, 30.2, 29.8, 29.0, 27.6, 26.9, 26.6, 24.4, 16.1, 14.5, 11.5. HRMS (MALDI): *m*/*z* calcd. for C_40_H_51_NNaO_9_S: 744.3182 [M + Na]^+^; found: 744.3163.

#### 2.3.13. Compound **10m**

The reaction was carried out using the general method, starting from pleuromutilin (95 mg, 0.25 mmol) and thiol **9m** (165 mg, 1.0 mmol, 2 × 2 equiv.) in MeOH at 0 °C, irradiating two times. The crude product was purified by flash column chromatography (CH_2_Cl_2_/MeOH 95/5) to result in compound **10m** as white powder (58 mg, 42%). R_f_: 0.69 (CH_2_Cl_2_/MeOH 7/3), [α]^24^_D_ +25.0 (*c* 0.12, MeOH), m.p. 113–114 °C. ^1^H NMR (400 MHz, Methanol-*d*_4_) δ 5.77 (d, *J* = 8.3 Hz, 1H), 4.19 − 3.93 (m, 2H), 3.47 (d, *J* = 5.9 Hz, 1H), 3.33 (p, *J* = 1.6 Hz, 1H), 3.09 (ddd, *J* = 9.1, 7.0, 2.2 Hz, 2H), 3.02 − 2.84 (m, 2H), 2.58 (td, *J* = 12.0, 4.7 Hz, 1H), 2.50 − 2.32 (m, 3H), 2.33 − 2.23 (m, 1H), 2.18 (d, *J* = 2.5 Hz, 1H), 1.91 (tdd, *J* = 25.3, 12.9, 4.1 Hz, 4H), 1.78 − 1.54 (m, 4H), 1.44 (s, 4H), 1.33 (d, *J* = 16.5 Hz, 2H), 1.27 − 1.08 (m, 1H), 1.05 (s, 3H), 0.97 (d, *J* = 7.0 Hz, 3H), 0.74 (d, *J* = 6.1 Hz, 3H). ^13^C NMR (101 MHz, Methanol-*d*_4_) δ 217.0, 172.0, 75.0, 69.0, 60.5, 57.9, 51.9, 45.5, 42.3, 41.7, 40.9, 36.7, 34.8, 33.9, 30.1, 30.0, 27.2, 26.7, 26.5, 25.9, 24.3, 15.6, 13.9, 10.4. HRMS (ESI): *m*/*z* calcd. for C_24_H_39_Na_2_O_8_S_2_: 565.1882 [M + Na]^+^; found: 565.1876.

#### 2.3.14. Compound **10n**

To the mixture of pleuromutilin (95 mg, 0.25 mmol) and thiol **9n** (57 mg, 54 µL, 0.75 mmol, 3 equiv.) in EtOAc at room temperature, 0.1 equivalent of DPAP (2,2-dimethoxy-2-phenylacetophenone) (0.025 mmol, 6 mg) and 0.1 equivalent of MAP (4-methoxyacetophenone) (0.025mmol, 4 mg) were added. It was irradiated once for one hour. The crude product was purified by flash column chromatography (*n*-hexane/acetone 8/2) to result in compound **10n** as white powder (91 mg, 80%). R_f_: 0.36 (*n*-hexane/acetone 6/4), [α]^24^_D_ +54.7 (*c* 0.15, MeOH), m.p. 188–189 °C. ^1^H NMR (400 MHz, Methanol-*d*_4_) δ 5.69 (d, *J* = 8.2 Hz, 1H), 4.14 − 3.87 (m, 2H), 3.44 (d, *J* = 6.1 Hz, 1H), 3.03 − 2.89 (m, 1H), 2.66 (td, *J* = 12.4, 5.0 Hz, 1H), 2.33 (d, *J* = 3.1 Hz, 2H), 2.30 (s, 4H), 2.28 − 2.17 (m, 1H), 2.18 − 2.05 (m, 1H), 1.97 − 1.77 (m, 5H), 1.73 − 1.51 (m, 3H), 1.42 (s, 4H), 1.31 (d, *J* = 16.4 Hz, 1H), 1.14 (td, *J* = 13.7, 4.4 Hz, 1H), 1.07 (s, 3H), 0.94 (d, *J* = 7.0 Hz, 3H), 0.71 (d, *J* = 6.6 Hz, 3H). ^13^C NMR (101 MHz, Methanol-*d*_4_) δ 217.0, 194.0, 168.0, 73.3, 67.3, 59.0, 56.4, 43.9, 40.2, 40.2, 39.6, 35.1, 33.4, 32.4, 28.6, 28.2, 27.6, 25.2, 24.2, 22.8, 14.1, 12.4, 8.9. HRMS (ESI): *m*/*z* calcd. for C_24_H_38_NaO_6_S: 477.2287 [M + Na]^+^; found: 477.2281.

#### 2.3.15. Compound **10o**

The reaction was carried out using the general method, starting from pleuromutilin (95 mg, 0.25 mmol) and thiol **9o** (45 mg, 60 µL, 0.5 mmol, 2 × 1 equiv.) in CH_3_CN/MeOH 2/1 at −40 °C, irradiating two times. The crude product was purified by flash column chromatography (CH_2_Cl_2_/acetone 95/5) to result in compound **10o** as white powder (97 mg, 82%). R_f_: 0.56 (CH_2_Cl_2_/acetone 9/1), [α]^24^_D_ +52.2 (*c* 0.18, CHCl_3_), m.p. 177–180 °C. ^1^H NMR (400 MHz, Chloroform-*d*) δ 5.65 (d, *J* = 8.3 Hz, 1H), 4.01 (q, *J* = 17.1 Hz, 2H), 3.38 (d, *J* = 5.8 Hz, 1H), 2.51 (dd, *J* = 7.9, 6.8 Hz, 2H), 2.41 (t, *J* = 8.3 Hz, 2H), 2.37 − 2.27 (m, 2H), 2.24 − 2.15 (m, 1H), 2.13 (s, 2H), 1.91 (t, *J* = 8.4 Hz, 2H), 1.83 − 1.68 (m, 2H), 1.62 − 1.41 (m, 7H), 1.36 (s, 5H), 1.30 − 1.18 (m, 2H), 1.08 (td, *J* = 13.8, 4.3 Hz, 1H), 0.99 (s, 3H), 0.92 (d, *J* = 7.0 Hz, 3H), 0.88 (t, *J* = 7.3 Hz, 3H), 0.64 (d, *J* = 6.8 Hz, 3H). ^13^C NMR (101 MHz, Chloroform-*d*) δ 217.1, 172.3, 76.0, 69.9, 61.3, 58.3, 45.5, 42.4, 41.8, 41.2, 36.5, 34.5, 34.4, 31.9, 31.8, 30.1, 30.1, 27.4, 26.8, 26.8, 24.8, 22.0, 16.5, 14.7, 13.7, 11.1. HRMS (ESI): *m*/*z* calcd. for C_26_H_44_NaO_5_S: 491.2807 [M + Na]^+^; found: 491.2801.

#### 2.3.16. Compound **10p**

The reaction was carried out using the general method, starting from pleuromutilin (95 mg, 0.25 mmol) and thiol **9p** (73 mg, 87 µL, 0.5 mmol, 2 equiv.) in CH_3_CN/MeOH 2/1 at −40 °C, irradiating once. The crude product was purified by flash column chromatography (CH_2_Cl_2_/acetone 95/5) to result in compound **10p** as white amorphous solid (96 mg, 73%). R_f_: 0.66 (CH_2_Cl_2_/acetone 9/1), [α]^24^_D_ +20.0 (*c* 0.04, CHCl_3_). ^1^H NMR (400 MHz, Chloroform-*d*) δ 5.68 (d, *J* = 8.3 Hz, 1H), 4.04 (qd, *J* = 17.0, 4.1 Hz, 2H), 3.41 (d, *J* = 5.8 Hz, 1H), 2.54 (t, *J* = 7.4 Hz, 2H), 2.45 (dt, *J* = 9.3, 3.0 Hz, 2H), 2.41 − 2.28 (m, 2H), 2.28 − 2.14 (m, 2H), 2.09 (d, *J* = 2.7 Hz, 1H), 1.94 (ddd, *J* = 9.9, 6.2, 2.4 Hz, 2H), 1.81 − 1.74 (m, 2H), 1.68 − 1.43 (m, 7H), 1.40 (s, 5H), 1.28 (dt, *J* = 10.0, 5.1 Hz, 10H), 1.11 (td, *J* = 13.8, 4.4 Hz, 1H), 1.03 (s, 3H), 0.96 (d, *J* = 7.0 Hz, 3H), 0.91 − 0.82 (m, 3H), 0.67 (d, *J* = 6.9 Hz, 3H). ^13^C NMR (101 MHz, Chloroform-*d*) δ 216.7, 172.3, 76.1, 70.0, 61.3, 58.3, 45.5, 42.4, 41.8, 41.2, 36.5, 34.5, 34.4, 32.3, 31.8, 30.1, 30.1, 29.8, 29.3, 29.2, 29.0, 27.4, 26.8, 26.8, 24.9, 22.6, 16.6, 14.7, 14.1, 11.1. HRMS (ESI): *m*/*z* calcd. for C_30_H_52_NaO_5_S: 547.3433 [M + Na]^+^; found: 547.3427.

#### 2.3.17. Compound **10q**

The reaction was carried out using the general method, starting from pleuromutilin (95 mg, 0.25 mmol) and thiol **9q** (202 mg, 240 µL, 1.0 mmol, 2 × 2 equiv.) in EtOH at 0 °C, irradiating two times. The crude product was purified by flash column chromatography (CH_2_Cl_2_/acetone 95/5) to result in compound **10q** as white amorphous solid (61 mg, 42%). R_f_: 0.66 (CH_2_Cl_2_/acetone 9/1), [α]^24^_D_ +47.4 (*c* 0.19, CHCl_3_). ^1^H NMR (400 MHz, Chloroform-*d*) δ 5.72 (d, *J* = 8.3 Hz, 1H), 4.06 (q, *J* = 17.0 Hz, 2H), 3.43 (d, *J* = 6.0 Hz, 1H), 2.57 (t, *J* = 7.4 Hz, 2H), 2.48 (td, *J* = 10.6, 9.7, 6.1 Hz, 2H), 2.40 (dd, *J* = 13.8, 6.1 Hz, 2H), 2.31 − 2.16 (m, 2H), 2.11 (s, 1H), 1.97 (dt, *J* = 10.8, 4.8 Hz, 2H), 1.82 (td, *J* = 15.5, 14.2, 5.8 Hz, 2H), 1.73 − 1.48 (m, 7H), 1.42 (d, *J* = 9.2 Hz, 5H), 1.28 (d, *J* = 5.3 Hz, 18H), 1.14 (td, *J* = 13.8, 4.3 Hz, 1H), 1.06 (s, 3H), 0.99 (d, *J* = 7.0 Hz, 3H), 0.89 (t, *J* = 6.6 Hz, 3H), 0.70 (d, *J* = 6.9 Hz, 3H). ^13^C NMR (101 MHz, Chloroform-*d*) δ 216.7, 172.3, 76.2, 70.1, 61.3, 58.3, 45.5, 42.5, 41.9, 41.2, 36.5, 34.5, 34.4, 32.4, 31.9, 30.1, 30.1, 29.8, 29.7, 29.6, 29.6, 29.3, 29.3, 29.0, 27.4, 26.8, 26.8, 24.9, 22.7, 16.5, 14.7, 14.1, 11.0. HRMS (ESI): *m*/*z* calcd. for C_34_H_60_NaO_5_S: 603.4059 [M + Na]^+^; found: 603.4052.

#### 2.3.18. Compound **10r**

The reaction was carried out using the general method, starting from pleuromutilin (95 mg, 0.25 mmol) and thiol **9r** (186 mg, 177 µL, 1.5 mmol, 3 × 2 equiv.) in toluene at −40 °C, it was irradiated three times. The crude product was purified by flash column chromatography (CH_2_Cl_2_/acetone 97/3) to result in compound **10r** as white powder (100 mg, 79%). R_f_: 0.60 (CH_2_Cl_2_/acetone 9/1), [α]^24^_D_ +67.1 (*c* 0.21, CHCl_3_), m.p. 150–151 °C. ^1^H NMR (400 MHz, Methanol-*d*_4_) δ 7.45 − 7.40 (m, 2H), 7.30 (td, *J* = 7.1, 6.1, 1.3 Hz, 2H), 7.25 − 7.19 (m, 1H), 5.74 (d, *J* = 8.3 Hz, 1H), 4.13 − 3.95 (m, 2H), 3.88 (d, *J* = 13.5 Hz, 1H), 3.74 (d, *J* = 13.5 Hz, 1H), 3.41 (d, *J* = 6.0 Hz, 1H), 2.37 (ddd, *J* = 9.7, 5.8, 2.5 Hz, 2H), 2.33 − 2.29 (m, 1H), 2.29 − 2.21 (m, 1H), 2.17 (s, 2H), 2.04 (ddd, *J* = 13.7, 10.3, 6.5 Hz, 1H), 1.95 − 1.77 (m, 4H), 1.64 (dt, *J* = 11.4, 6.7 Hz, 4H), 1.44 (s, 4H), 1.23 (d, *J* = 16.2 Hz, 1H), 1.15 (td, *J* = 13.4, 4.3 Hz, 1H), 0.98 (s, 3H), 0.83 (d, *J* = 6.9 Hz, 3H), 0.75 (d, *J* = 6.0 Hz, 3H). ^13^C NMR (101 MHz, Methanol-*d*_4_) δ 217.0, 171.9, 139.2, 128.7, 128.0, 126.4, 74.9, 68.9, 60.6, 57.9, 45.5, 42.5, 41.7, 40.8, 36.7, 35.1, 34.8, 33.9, 30.8, 30.0, 26.8, 26.3, 26.1, 24.4, 15.7, 14.0, 10.5. HRMS (ESI): *m*/*z* calcd. for C_29_H_42_NaO_5_S: 525.2651 [M + Na]^+^; found: 525.2643.

#### 2.3.19. Compound **11g**

A catalytic amount of NaOMe (pH ~ 9) was added to a stirred solution of compound **10g** (65 mg, 0.09 mmol) in dry MeOH (5 mL) and stirred for one hour at room temperature. The reaction mixture was neutralized with Amberlite IR-120 H^+^ ion-exchange resin, filtered and evaporated. Then, the crude product was purified by flash column chromatography (CH_2_Cl_2_/MeOH 8/2) to result in compound **11g** as white amorphous solid (42 mg, 80%). R_f_: 0.50 (CH_2_Cl_2_/MeOH 8/2), [α]^24^_D_= +26.4 (*c* 0.14, MeOH). ^1^H NMR (400 MHz, Methanol-*d*_4_) δ 5.74 (d, *J* = 8.3 Hz, 1H), 5.51 (s, 1H), 4.53 − 4.45 (m, 1H), 4.05 (m, 2H), 3.89 (d, *J* = 0.9 Hz, 1H), 3.85 − 3.76 (m, 1H), 3.75 − 3.66 (m, 2H), 3.56 − 3.51 (m, 2H), 3.46 (d, *J* = 5.9 Hz, 1H), 3.37 (s, 1H), 2.63 (tq, *J* = 17.8, 6.4, 5.4 Hz, 2H), 2.42 − 2.05 (m, 5H), 2.02 − 1.78 (m, 3H), 1.76 − 1.53 (m, 3H), 1.52 − 1.28 (m, 7H), 1.22 − 1.11 (m, 1H), 1.05 (s, 3H), 0.96 (d, *J* = 7.0 Hz, 3H), 0.74 (d, J = 6.5 Hz, 3H). ^13^C NMR (101 MHz, Methanol-*d*_4_) δ 211.6, 173.5, 88.2, 80.4, 76.5, 76.2, 71.7, 70.7, 70.4, 63.1, 61.9, 59.3, 46.8, 43.1, 42.8, 42.3, 38.0, 36.1, 35.3, 31.7, 31.4, 28.1, 27.4, 26.9, 25.7, 17.0, 15.3, 11.7. HRMS (ESI): *m*/*z* calcd. for C_28_H_46_NaO_10_S [M + Na]^+^ 597.2709, found: 597.2704.

#### 2.3.20. Compound **11h**

To a stirred solution of compound **10h** (65 mg, 0.073 mmol) in dioxane:water = 9:1 (2 mL) 0.2 M aqueous solution of KOH (1.83 mL, 0.36 mmol, 5 equiv.) was added, and the reaction mixture was stirred for half an hour at 0 °C, then for 3 h at room temperature. The reaction mixture was neutralized with Amberlite IR-120 H^+^ ion-exchange resin, filtered and evaporated to obtain compound **11h** as yellow amorphous solid (50 mg, 97%). R_f_: 0.56 (CH_3_CN/H_2_O 9/1), [α]^24^_D_ + 28.9 (*c* 0.09, MeOH). ^1^H NMR (400 MHz, DMSO-*d*_6_) *δ* 5.55 (d, *J* = 7.6 Hz, 1H, H-14), 4.09 (d, *J* = 5.7 Hz, 2H, H-22ab), 3.75 (dd, *J* = 19.5, 10.4 Hz, 4H), 3.63 − 3.56 (m, 2H), 3.51 (d, *J* = 9.7 Hz, 2H), 3.43 (d, *J* = 4.9 Hz, 1H, H-11), 3.27 (d, *J* = 1.2 Hz, 14H), 2.73 (dd, *J* = 12.4, 4.0 Hz, 1H), 2.65 − 2.56 (m, 1H), 2.47 (dd, *J* = 11.5, 3.7 Hz, 1H, H-19*a), 2.41 (s, 1H, H-19*b), 2.29 − 2.10 (m, 4H), 1.95 (s, 1H, H-20*a), 1.85 (s, 5H, H-20*b), 1.75 (d, *J* = 14.4 Hz, 1H, H-1a), 1.66 (d, *J* = 11.9 Hz, 2H), 1.59 − 1.50 (m, 1H), 1.49 − 1.39 (m, 4H, H-1b), 1.30 (s, 5H), 1.21 (dd, *J* = 29.0, 9.0 Hz, 6H), 1.09 − 0.97 (m, 2H), 0.91 (s, 3H, H18abc), 0.83 (d, *J* = 6.2 Hz, 3H, H-17abc), 0.60 (d, *J* = 5.3 Hz, 3H, H-16abc). * Interchangeable signals. ^13^C NMR (101 MHz, DMSO-*d*_6_) *δ* 174.3, 172.6, 171.1 (3C, C-1′, C-21, Ac*C*O), 85.1 (1C, C-2′), 75.2, 73.9, 71.1, 69.2, 67.4, 66.9 53.6, 51.9 (8C, skeletal carbons, C-11, C-14), 63.4, 60.4, (2C, C-22, C-9′), 57.5 (1C, C-4), 45.0, 41.3, 40.8 (4C, C-12, C-13, C-5, C-9), 36.4 (1C, C-6), 34.0, 29.0, 27.4 (3C, C-19, C-20, C-8), 26.9 (1C, C-18), 25.5 (1C, C-1), 24.5 (1C, C-7), 22.3 (1C, Ac*C*H_3_), 16.1 (1C, C-16), 14.6 (1C, C-15), 11.6 (1C, C-17). HRMS (MALDI): *m*/*z* calcd. for C_33_H_53_NNaO_13_S [M + Na]^+^ 726.3135, found: 726.3159.

#### 2.3.21. Compound **11l**

Compound **10l** (100 mg, 0.138 mmol) was dissolved in 20% piperidine solution in *N*,*N*-dimethylformamide (5 mL) and stirred for two hours at room temperature. The reaction mixture was evaporated, and the crude product was purified by flash column chromatography (CH_3_CN/H_2_O 9/1) to result in compound **11l** as yellow powder (46 mg, 66%). R_f_: 0.10 (CH_3_CN/H_2_O 9/1), [α]^24^_D_ +41.7 (*c* 0.06, MeOH), m.p. 197–199 °C. ^1^H NMR (400 MHz, Methanol-*d*_4_) δ 5.69 (t, *J* = 8.6 Hz, 1H), 4.22 (dd, *J* = 25.1, 17.2 Hz, 1H), 4.04 (dd, *J* = 17.2, 13.4 Hz, 1H), 3.79 (dt, *J* = 10.9, 5.2 Hz, 1H), 3.47 (t, *J* = 5.0 Hz, 1H), 3.37 (s, 2H), 3.33 (p, *J* = 1.7 Hz, 1H), 3.22 (dd, *J* = 15.2, 4.8 Hz, 1H), 3.19 − 3.07 (m, 1H), 2.52 (tq, *J* = 10.8, 5.2, 4.8 Hz, 2H), 2.41 − 2.31 (m, 2H), 2.31 − 2.24 (m, 1H), 2.17 (q, *J* = 9.6 Hz, 1H), 2.13 − 1.98 (m, 1H), 1.98 − 1.48 (m, 5H), 1.45 (d, *J* = 3.7 Hz, 5H), 1.39 − 1.27 (m, 2H), 1.17 (td, *J* = 13.8, 4.3 Hz, 1H), 1.03 (d, *J* = 5.0 Hz, 3H), 0.95 (dd, *J* = 7.1, 1.8 Hz, 3H), 0.74 (dd, *J* = 7.0, 2.0 Hz, 3H). ^13^C NMR (101 MHz, Methanol-*d*_4_) δ 217.0, 190.0, 173.3, 74.9, 69.3, 60.5, 57.8, 53.8, 45.4, 41.6, 41.0, 36.6, 34.6, 33.9, 32.8, 30.0, 28.4, 28.1, 27.2, 26.8, 25.8, 24.2, 15.8, 13.9, 10.3. HRMS (ESI): *m*/*z* calcd. for C_25_H_41_NNaO_7_S [M + Na]^+^ 522.2501, found: 522.2496.

#### 2.3.22. Compound **13a**

Nonafluoro-1-iodobutane (103 μL, 0.6 mmol, 1.2 equiv.) and benzophenone (5 mg, 0.023 mmol) were added to the solution of pleuromutilin 1 (189 mg, 0.5 mmol) in methanol (5 mL). Argon gas was bubbled through the solution, and then irradiation occurred with UV light for 10 min. The solvent was evaporated, and the product was purified by flash column chromatography (CH_2_Cl_2_/acetone 95:5) to yield **12a** (163 mg, 45%) as a colorless liquid. R_f_ = 0.51 (CH_2_Cl_2_/MeOH 95:5). To the solution of **12a** (130 mg, 0.18 mmol), methanol (5 mL), 10% palladium on activated charcoal (25 mg) and NaHCO_3_ (53 mg, 2.5 equiv.) were added. The reaction mixture was stirred overnight under H_2_ atmosphere, then filtered through a pad of Celite, and the solvent was evaporated. The residue was dissolved in dichloromethane (50 mL) and the solution was washed with distilled water (10 mL) two times, and dried over anhydrous Na_2_SO_4_. The solvent was then evaporated in vacuum. The product was purified by flash column chromatography (CH_2_Cl_2_/acetone 97:3 then 95:5) to yield **13a** (36 mg, 34%) as a colorless liquid. Rf = 0.25 (CH_2_Cl_2_/acetone 97:3), [α]^24^_D_ +11.8 (*c* 0.22, CHCl_3_). ^1^H NMR (400 MHz, Chloroform-*d*) δ 5.63 (d, *J* = 8.2 Hz, 1H, H-14), 4.77 (dd, *J* = 9.3, 6.5 Hz, 1H), 4.20 − 4.12 (m, 1H, H-22a), 4.06 (dd, *J* = 18.3, 5.8 Hz, 1H, H-22b), 3.62 (s, 1H, H-11), 3.21 (d, *J* = 6.0 Hz, 1H), 2.62 (t, *J* = 5.3 Hz, 1H), 2.44 − 2.37 (m, 1H), 2.24 (dd, *J* = 17.1, 8.2 Hz, 3H), 2.16 (s, 1H, H-2b), 1.92 (s, 3H), 1.82 (d, *J* = 16.2 Hz, 2H), 1.77 − 1.70 (m, 2H), 1.64 (dd, *J* = 16.2, 8.3 Hz, 1H), 1.51 (t, *J* = 8.6 Hz, 2H), 1.44 (s, 3H, H-15abc), 1.18 (d, *J* = 6.8 Hz, 1H), 1.16 − 1.09 (m, 1H), 1.05 (s, 3H, H-18abc), 0.94 (d, *J* = 7.0 Hz, 3H, H-17abc), 0.69 (t, *J* = 6.0 Hz, 3H, H-16abc). ^13^C NMR (101 MHz, Chloroform-*d*) δ 174.9 (1C, C-21), 74.7, 71.1 (2C, C-11, C-14), 63.3, 61.5 (1C, C-22), 58.6, 46.4, 45.8, 42.0, 40.8 (4C, C-5, C-12, C-13, C-9), 36.3, 34.9 (2C, C-6, C-10), 34.4, 34.2 (2C, C-2, C-23), 29.8, 27.1 (2C, C-7, C-19), 24.7, 20.5, 17.0 (1C, C-16), 14.6 (1C, C-15), 10.5 (1C, C-17). HRMS (ESI): *m*/*z* calcd. for C_26_H_35_F_9_KO_5_ [M + K]^+^ 637.2188, found: 637.2182.

#### 2.3.23. Compound **13b**

Heptadecafluoro-1-iodooctane (0.160 mL, 327 mg, 0.6 mmol, 1.2 equiv.) and benzophenone (5 mg, 0.023 mmol) were added to the solution of pleuromutilin 1 (0.189 g, 0.5 mmol) in methanol (5 mL). Argon gas was bubbled through the solution and then irradiation occurred with UV light for 10 min. The solvent was evaporated, and the product was purified by flash column chromatography (CH_2_Cl_2_/acetone 97:3) to yield **12b** (247 mg, 53%) as a colorless liquid. R_f_ = 0.55 (CH_2_Cl_2_/acetone 95:5). To the solution of **12b** (0.120 g, 0.25 mmol) in methanol (5 mL) 10% palladium on activated charcoal (25 mg) and NaHCO_3_ (53 mg, 2.5 equiv.) were added. The reaction mixture was stirred overnight under H_2_ atmosphere, then filtered through a pad of Celite, and the solvent was evaporated. The residue was dissolved in dichloromethane (50 mL) and the solution was washed with distilled water (10 mL) two times, and dried with anhydrous Na_2_SO_4_. Then, the solvent was evaporated in vacuum. The product was purified by flash column chromatography (CH_2_Cl_2_/acetone 97:3) to yield **13b** (49 mg, 48%) as a colorless liquid. Rf=0.22 (CH_2_Cl_2_/acetone 95:5), [α]^24^_D_ +4.71 (*c* 0.34, CHCl_3_). ^1^H NMR (400 MHz, Chloroform-*d*) δ 5.64 (d, *J* = 8.2 Hz, 1H), 4.78 (dd, *J* = 9.0, 4.9 Hz, 1H), 4.20 − 4.00 (m, 2H), 3.62 (d, *J* = 4.0 Hz, 1H), 3.19 (s, 1H), 2.59 (s, 7H), 2.47 − 2.36 (m, 1H), 2.24 (dd, *J* = 17.1, 8.4 Hz, 3H), 1.88 (s, 1H), 1.82 (d, *J* = 16.2 Hz, 2H), 1.78 − 1.59 (m, 4H), 1.51 (dd, *J* = 11.5, 5.9 Hz, 2H), 1.44 (s, 3H, H-15abc), 1.17 (s, 1H), 1.16 − 1.09 (m, 1H), 1.05 (s, 3H, H-18abc), 0.94 (d, *J* = 7.0 Hz, 3H, H-17abc), 0.70 (d, *J* = 7.0 Hz, 3H, H-16abc). ^13^C NMR (101 MHz, Chloroform-*d*) δ 174.9 (1C, C-21), 74.8, 71.1 (2C, C-11, C-14), 63.4, 61.6 (1C, C-22), 58.6 (1C, C-4), 46.4, 45.9, 42.0, 40.9 (4C, C-5, C-12, C-13, C-9), 36.3, 34.4, 34.2, 29.9, 27.1, 24.7, 20.5, 16.9 (1C, C-16), 14.7 (1C, C-15), 10.5 (1C, C-17). HRMS (ESI): *m*/*z* calcd. for C_30_H_35_F_17_KO_5_ [M + K]^+^ 837.2060, found: 837.2054.

#### 2.3.24. Compound **14h**

The reaction was carried out using the general method, starting from lefamulin (51 mg, 0.1 mmol), trifluoroacetic acid (15 µL, 0.2 mmol, 2 equiv.) and thiol **9h** (203 mg, 0.4 mmol, 2 × 2 equiv.) in methanol at −80 °C. It was irradiated two times. The reaction mixture was co-evaporated with toluene three times and the crude product was purified by flash column chromatography (CH_2_Cl_2_/MeOH 85/15) to result in compound **14h** as white amorphous solid (79 mg, 78%). R_f_: 0.56 (CH_2_Cl_2_/MeOH 7/3), [α]^24^_D_ +24.4 (*c* 0.09, DMSO). ^1^H NMR (400 MHz, DMSO-*d*_6_) *δ* 7.77 (d, *J* = 6.5 Hz, 1H, N*H*), 5.42 (d, *J* = 6.8 Hz, 1H, H-14), 5.20 (d, *J* = 2.3 Hz, 1H, H-8″), 5.16 (d, *J* = 7.1 Hz, 1H), 4.69 (s, 1H, H-4″), 4.65 (s, 1H, O*H*), 4.21 (d, *J* = 12.1 Hz, 1H, H-9″a), 4.04 (dd, *J* = 11.6, 4.5 Hz, 1H, H-9″b), 3.84 (s, 1H, H-5″), 3.80 (d, *J* = 1.6 Hz, 1H), 3.54 (d, *J* = 14.5 Hz, 1H), 3.42 (s, 5H), 3.33 (d, *J* = 12.8 Hz, 3H, H-11, H-2′), 3.02 (s, 1H, H-4′), 2.64 (d, *J* = 8.7 Hz, 1H, H-3″a), 2.56 (s, 1H, H-1′), 2.51 (d, *J* = 1.4 Hz, 2H), 2.35 (s, 1H, H-4), 2.16 (d, *J* = 10.9 Hz, 2H, H-5′a, H-10), 2.07 (d, *J* = 1.7 Hz, 3H, AcC*H*_3_), 2.01 (s, 3H, AcC*H*_3_), 1.96 (s, 3H, AcC*H*_3_), 1.92 (d, *J* = 1.7 Hz, 3H, AcC*H*_3_), 1.83 (d, *J* = 16.5 Hz, 1H, H-13a), 1.75 (d, *J* = 12.3 Hz, 1H, H-3″b), 1.65 (s, 5H, AcC*H*_3_, H-7a, H-8a), 1.49 (s, 2H, H-6), 1.37 (s, 3H, H-15abc), 1.23 (s, 6H, H-7b), 1.13 (d, *J* = 16.1 Hz, 2H, H-13b), 1.08 − 0.95 (m, 2H, H-8b), 0.90 (s, 3H, H-18abc), 0.80 (d, *J* = 5.4 Hz, 3H, H-17abc), 0.64 (d, *J* = 5.7 Hz, 3H, H-16abc). ^13^C NMR (101 MHz, DMSO-*d*_6_) *δ* 217.1 (1C, C-3), 170.1, 169.7, 169.4, 169.3, 169.3, 169.1, 168.4 (7C, 7×*C*O), 83.1 (1C, C-2″), 73.7, 73.3, 72.3, 67.3 (4C, C-11, C-2′, C-7″, C-6″), 69.6 (1C, C-4″), 68.9 (1C, C-14), 68.4 (1C, C-8″), 61.8 (1C, C-9″), 57.2 (1C, C-4), 54.9, 53.0 (1C, COO*C*H_3_), 49.7 (1C, C-1′), 47.8 (1C, C-5″), 47.2 (1C, C-4′), 44.9 (1C, C-9), 41.2, 40.6 (3C, C-13, C-12, C-5), 36.4 (1C, C-6), 34.4 (1C, C-10), 33.9 (1C, C-5′), 33.7 (1C, C-22), 30.1 (1C, C-8), 29.8, 29.6, 28.2, 26.8, 23.7 (6C, C-19, C-20, C-3′, C-6′, C-1, C-2), 26.2 (1C, C-18), 24.3 (1C, C-7), 22.6 (1C, NAc*C*H_3_), 20.9, 20.7, 20.6, 20.6 (4C, 4xOAc*C*H_3_), 16.8 (1C, C-16), 14.6 (1C, C-15), 11.5 (1C, C-17). HRMS (ESI): *m*/*z* calcd. for C_48_H_75_N_2_O_17_S_2_ [M + H]^+^ 1015.4507, found: 1015.4502.

#### 2.3.25. Compound **14j**

The reaction was carried out using the general method, starting from lefamulin (51 mg, 0.1 mmol), trifluoroacetic acid (15.3 µL, 0.2 mmol, 2 equiv.) and thiol **9j** (31 mg, 28 µL, 0.4 mmol, 2 × 2 equiv.) in methanol at −80 °C. It was irradiated two times. The reaction mixture was co-evaporated with toluene three times and the crude product was purified by flash column chromatography (CH_3_CN/H_2_O 9/1) to result in compound **14j** as white-yellow amorphous solid (35 mg, 60%). R_f_: 0.19 (CH_3_CN/H_2_O 9/1), [α]^24^_D_ +50.0 (*c* 0.07, H_2_O). ^1^H NMR (400 MHz, DMSO-*d*_6_) *δ* 5.51 (d, *J* = 7.6 Hz, 1H, H-14), 4.61 (s, 1H, O*H*), 3.63 − 3.46 (m, 3H, H-24ab), 3.40 − 3.29 (m, 3H, H-11, H-2′), 2.98 (s, 1H, H-4′), 2.57 (t, *J* = 7.0 Hz, 2H, H-23ab), 2.51 (s, 2H), 2.37 (t, *J* = 13.0 Hz, 3H, H-4), 2.23 − 2.02 (m, 3H, H-10, H-3′a), 1.96 (s, 3H), 1.91 − 1.78 (m, 2H, H-13a, H-5′a), 1.67 (t, *J* = 16.9 Hz, 3H, H-7a, H-8a), 1.49 (d, *J* = 5.9 Hz, 1H, H-6), 1.42 (d, *J* = 15.5 Hz, 1H), 1.37 (s, 3H, H-15abc), 1.33 − 1.18 (m, 7H, H-7b, H-3′a, H-5′b, H-6′ab), 1.12 (d, *J* = 16.0 Hz, 1H, H-13b), 1.07 − 0.98 (m, 1H, H-8b), 0.93 (s, 3H, H-18abc), 0.83 (d, *J* = 6.3 Hz, 3H, H-17abc), 0.64 (d, *J* = 6.2 Hz, 3H, H-16abc). ^13^C NMR (101 MHz, DMSO-*d*_6_) *δ* 217.1 (1C, C-3), 169.3 (1C, C-21), 73.5, 72.3 (2C, C-11, C-2′), 68.7 (1C, C-14), 61.0 (1C, C-24), 57.2 (1C, C-4), 49.5 (1C, C-1′), 47.3 (1C, C-4′), 45.0, 41.3, 40.6, 40.3 (4C, C-5, C-13, C-12, C-9), 36.4 (1C, C-6), 34.6 (1C, C-10), 33.9, 33.5, 30.5, 30.1, 28.3, 27.0, 26.7 (8C, C-3′, C-5′, C-6′, C-1, C-2, C-19, C-20, C-23), 26.9 (1C, C-18), 24.3 (1C, C-7), 16.5 (1C, C-16), 14.6 (1C, C-15), 11.5 (1C, C-17). HRMS (ESI): *m*/*z* calcd. for C_30_H_52_NO_6_S_2_ [M + H]^+^ 586.3236, found: 586.3230.

#### 2.3.26. Compound **14k**

The reaction was carried out using the general method, starting from lefamulin (50.8 mg, 0.1 mmol), trifluoroacetic acid (15.3 µL, 0.2 mmol, 2 equiv.) and thiol **9k** (33 mg, 0.2 mmol, 2 × 1 equiv.) in methanol at −80 °C. It was irradiated two times. The reaction mixture was evaporated with added toluene three times and the crude product was purified by flash column chromatography (CH_3_CN/H_2_O 9/1) resulted in compound **14k** as white-yellow amorphous solid (57.4 mg, 86%). R_f_: 0.46 (CH_3_CN/H_2_O 85/15), [α]^24^_D_ +36.0 (*c* 0.2, H_2_O). ^1^H NMR (400 MHz, DMSO-*d*_6_) *δ* 7.42 (d, *J* = 7.1 Hz, 1H, N*H*), 5.50 (d, *J* = 8.0 Hz, 1H, H-14), 4.57 (d, *J* = 4.4 Hz, 1H, O*H*), 4.08 (dd, *J* = 12.1, 5.4 Hz, 1H, H-24), 3.68 (d, *J* = 16.2 Hz, 2H, H-22a), 3.35 (dd, *J* = 21.8, 11.0 Hz, 2H, 10H, H-2′, H-11), 3.19 − 3.13 (m, 2H, H-22b), 2.95 (dt, *J* = 19.3, 9.7 Hz, 2H, H-23a, H-4′), 2.74 − 2.60 (m, 2H, H-23b, H-1′), 2.53 − 2.49 (m, 2H), 2.42 − 2.32 (m, 2H, H-4), 2.32 − 2.22 (m, 1H), 2.18 (dd, *J* = 12.1, 6.1 Hz, 2H, H-10, H-5′a), 2.03 (ddd, *J* = 21.3, 17.6, 5.7 Hz, 4H, H-5′b), 1.84 (s, 3H, AcCH_3_), 1.79 (dd, *J* = 13.9, 9.4 Hz, 3H, H-13a), 1.72 − 1.60 (m, 6H, H-7a, H-8a), 1.48 (dd, *J* = 11.9, 9.0 Hz, 1H, H-6), 1.41 (d, *J* = 13.3 Hz, 1H, H-6′a), 1.33 (s, 3H, H-15abc), 1.31 − 1.23 (m, 3H, H-7b), 1.18 (dd, *J* = 9.7, 6.4 Hz, 2H, H-13b), 1.07 − 0.97 (m, 1H, H-8b), 0.91 (s, 3H, H-18abc), 0.83 (d, *J* = 6.8 Hz, 3H, H-17abc), 0.60 (d, *J* = 6.6 Hz, 3H, H-16abc). ^13^C NMR (101 MHz, DMSO-*d*_6_) *δ* 217.3 (1C, C-3), 173.0 (1C, C-21), 169.0, 168.4 (2C, Ac*C*O, *C*OOH), 73.6 (2C, C-2′, C-11), 72.8, 68.5 (1C, C-14), 57.2 (1C, C-4), 53.9 (1C, C-24), 47.4, 47.2 (1C, C-4′, C-1′), 41.2 (1C, C-13), 45.0, 40.7 (3C, C-5, C-12, C-9), 36.4 (1C, C-6), 35.9 (1C, C-23), 34.5 (1C, C-10), 34.0 (1C, C-5′), 33.0 (1C, C-22), 30.1 (1C, C-8), 31.1, 29.2, 28.2, 27.7 (4C, C-19, C-20, C-3′, C-6′), 26.8 (1C, C-18), 24.3 (1C, C-7), 22.9 (1C, Ac*C*H_3_), 16.4 (1C, C-16), 14.6 (1C, C-15), 11.5 (1C, C-17). HRMS (MALDI): *m*/*z* calcd. for C_33_H_54_N_2_NaO_8_S_2_ [M + Na]^+^ 693.3219, found: 693.3219.

#### 2.3.27. Compound **15h**

To a stirred solution of compound **14h** (58 mg, 0.06 mmol) in dioxane: water = 9:1 (2 mL) and 1.5mL of 0.2 M aqueous solution of KOH (1.5 mL, 0.3 mmol, 5 equiv.) was added (pH = 12). The reaction mixture was stirred for half an hour at 0 °C, then stirred for 3 h at room temperature. The reaction mixture was neutralized with Amberlite IR-120 H^+^ ion-exchange resin, filtered, evaporated, and purified by flash column chromatography (CH_3_CN/H_2_O 9/1) to obtain compound **15h** as white amorphous solid (25 mg, 53%). R_f_: 0.4 (CH_3_CN/H_2_O 8/2), [α]^24^_D_ -3.33 (c 0.06, DMSO). ^1^H NMR (400 MHz, DMSO-*d*_6_) *δ* 8.05 (d, *J* = 7.9 Hz, 1H, N*H*), 5.49 (d, *J* = 7.8 Hz, 1H, H-14), 5.17 (s, 1H, H-8″), 4.87 (s, 1H), 4.69 (s, 1H, H-4″), 4.48 (d, *J* = 5.5 Hz, 1H), 4.08 (s, 1H), 3.60 (t, *J* = 14.9 Hz, 4H, H-5″, H-9″a, H-11, H-2′), 3.26 (s, 1H, H-9″b), 3.16 (s, 2H), 2.98 (s, 1H, H-4′), 2.89 (s, 1H), 2.74 (s, H), 2.69 (dd, *J* = 8.6, 3.6 Hz, 1H, H-22a), 2.63 (d, *J* = 12.8 Hz, 1H, H-1′), 2.56 (d, *J* = 6.7 Hz, 1H, H-22b), 2.35 (s, 1H, H-4), 2.21 − 2.08 (m, 3H, H-5′a, H-10), 2.03 (d, *J* = 9.2 Hz, 3H, H-5′b), 1.89 (s, 3H), 1.77 (t, *J* = 16.3 Hz, 4H, H-13a), 1.63 (dd, *J* = 21.1, 9.8 Hz, 3H, H-7a, H-8a), 1.48 (dd, *J* = 21.3, 10.7 Hz, 5H, H-6), 1.33 (s, 3H), 1.24 (s, 3H, H-7b), 1.19 (s, 3H, H-13b), 1.03 (dd, *J* = 16.5, 4.5 Hz, 2H, H-8b), 0.94 (s, 3H, H-18abc), 0.83 (d, *J* = 6.5 Hz, 3H, H-17abc), 0.61 (d, *J* = 6.3 Hz, 3H, H-16abc). ^13^C NMR (101 MHz, DMSO-*d*_6_) *δ* 217.2 (1C, C-3), 172.4, 172.2, 168.5 (3C, Ac*C*O, C-1″, C-21), 84.8 (1C, C-2″), 75.2, 73.5, 72.0, 71.2 (3C, C-11, C-2′), 68.8, 68.6 (2C, C-8″, C-14), 67.7, 63.2 (1C, C-9″), 57.2 (1C, C-4), 54.9, 52.8, 49.8 (1C, C-1′), 47.3, 47.1 (1C, C-4′), 44.9 (1C, C-9), 42.3, 41.2, 40.9 (3C, C-12, C-13, C-5), 36.4 (1C, C-6), 35.8, 34.4 (1C, C-10), 34.0 (1C, C-5′), 33.0 (1C, C-22), 31.1, 28.9, 30.1 (1C, C-8), 26.8 (1C, C-18), 24.4 (1C, C-7), 24.2 (1C, C-22), 22.5 (1C, Ac*C*H_3_), 16.4 (1C, C-, 6), 14.6 (1C, C-15), 11.4 (1C, C-17). HRMS (ESI): *m*/*z* calcd. for C_39_H_64_N_2_NaO_13_S_2_ [M + Na]^+^ 855.3748, found: 855.3746.

## 3. Results and Discussion

**Scheme 1:**
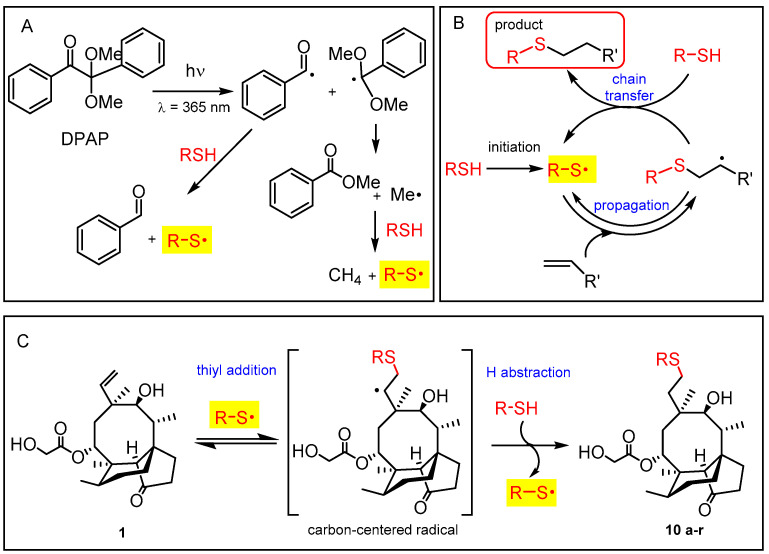
Generation of thiyl radical by the cleavable photoinitiator DPAP upon UV irradiation (**A**); free-radical chain process of hydrothiolation of alkenes (**B**); sequential thiyl-addition and hydrogen abstraction steps during the thiol-ene coupling reaction of pleuromutilin (**C**).

Firstly, the pleuromutilin skeleton was modified by the photoinitiated radical addition of a wide range of thiols to the C-C double bond. Hydrothiolation of terminal alkenes is known to occur by a free free-radical chain mechanism (Scheme 1) [20,21,22,23]. It is known from previous studies that the addition of thiols to terminal alkenes results in the novel C-S bond with complete regioselectivity, as the reaction goes under anti-Markovnikov-rule [30].

**Scheme 2:**
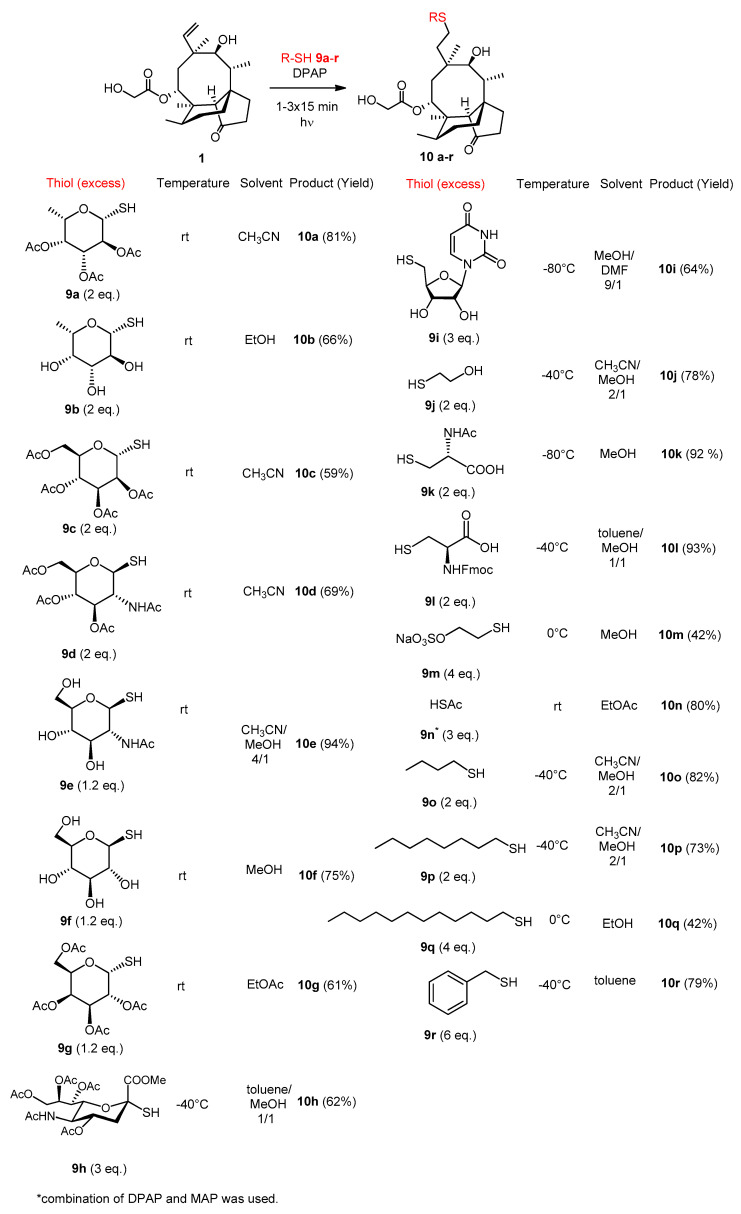
Hydrothiolation of pleuromutilin at position C19–C20 with thiols **9a**–**9r**.

For the introduction of hydrophilic substituents to position C20 per-*O*-acetylated or unprotected glycosyl thiols (α-l-fuco- **9a** and **9b**, α-d-manno- **9c**, β-d-Glc*N*Ac- **9d** and **9e**, β-d-gluco- **9f** and β-d-galactopyranose **9g**, and 5-*N*Ac-α-d-neuraminic acid **9h**), 5′-thio-thymidine **9i**, 2-mercaptoethanol **9j**, *N*-acetyl-l-cysteine **9k**, *N*-Fmoc-l-cysteine **9l**, Mesna **9m** and thioacetic acid **9n** were used. Alkyl thiols (*n*-butyl **9o**, *n*-octyl **9p**, *n*-dodecyl **9q**) and benzyl mercaptan (**9r**) were applied for the lipophilic modifications. The thiol-ene reactions were performed under the previously established conditions [19,20,21,22,23] using DPAP (2,2-dimethoxy-2-phenylacetophenone) as photoinitiator and irradiation with UV light at 365 nm (Scheme 2). We have demonstrated that for several short irradiation cycles, adding a new dose of initiator (which is consumed in the initiation step) to the reaction mixture before each cycle, is more effective than a longer-term continuous irradiation. Thus, if the conversion was not satisfactory after 15 min., the reaction mixture was subjected to UV-irradiation for further 1–2 × 15 min. The thiol-ene coupling is tolerant to water and oxygen and is compatible with any solvent [24,31,32,33]. Therefore, we performed the reactions without any caution to exclude air or moisture, and selected different solvents on the basis of the solubility of the reactants.

The desired compounds **10a**–**10r** were isolated with moderate (42%) to excellent (94%) yields and full regioselectivity. Recently, we have demonstrated that the reaction temperature influences the efficacy of the photoinitiated thiol-ene reactions in a unique way. Cooling promotes, whereas heating inhibits, the thiol-ene couplings [20,21,22,24,31,32,33]. Cooling exerts the beneficial effect by increasing the half-life of the carbon centered radical intermediate, thus shifting the equilibrium of the reversible step toward product formation, and increasing the yield [20,21,23]. Therefore, when a low conversion was observed at room temperature, the reaction was repeated by cooling, in the temperature range of 0 °C to −80 °C. The experimental set-up of the low-temperature photoinitiated thiol-ene coupling reactions is shown in Supplementary Materials (Figure S28). In the case of compound **9n**, only moderate yield was achieved by the standard DPAP-catalyzed addition reaction. In this case, a synergistic photoinitiator combination of MAP (4′-methoxyacetophenone) and DPAP was used, based on Scalan’s work on the successful addition of acyl thiols to alkenes [34]. In each case, the most effective conditions used are presented. The ^13^C NMR measurements confirmed the formation of the new C-S bond, namely, the signals of the C=C double bond disappeared (absence of signals around 140 and 115 ppm) and the characteristic peak of the new -CH_2_-S group appeared at ~30 ppm in the carbon spectra of the products.

**Scheme 3:**
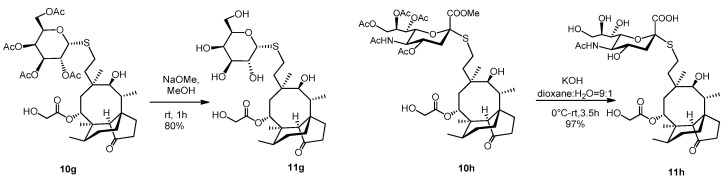
Successful deacetylation of the galactose-modified pleuromutilin **10g** and hydrolysis of the sialic-acid derivative **10h** by KOH.

The C14 glycolate side chain is essential for the antibacterial activity [8] that had to be taken into account when performing basic deprotection of the ester protecting groups of **10g** and **10h**. Zemplén-deacetylation of compound **10g** with a catalytic amount of NaOMe proceeded with excellent yield, without affecting the glycolate ester bond. Moreover, the core antibiotic survived the KOH-mediated removal of the methyl ester group of the sialic acid derivative **10h** (Scheme 3).

**Scheme 4:**
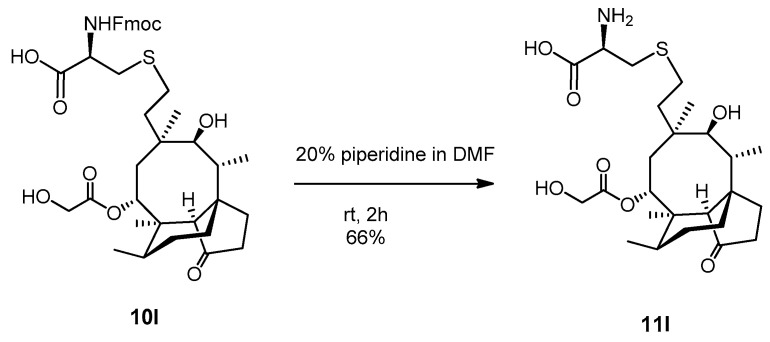
Deprotection of compound **10l**.

The Fmoc group of the cysteine-derivative **10l** was removed using piperidine, resulting in compound **11l** with a yield of 66% (Scheme 4).

**Scheme 5:**
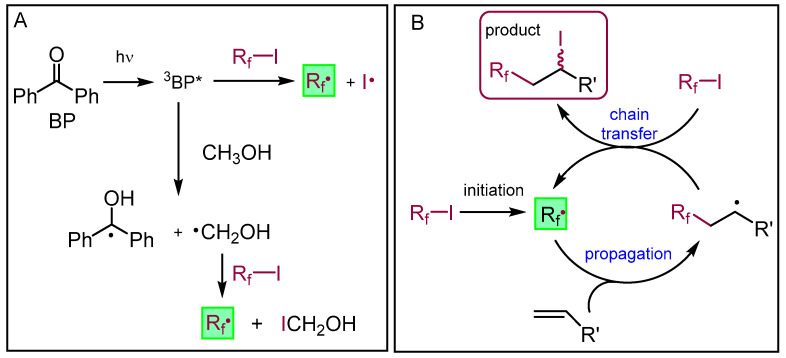
(**A**) Generation of the R_f_· radical from perfluoroalkyl iodide with benzophenone in MeOH under UV irradiation (^3^BP*: benzophenone triplet); (**B**) mechanism of ATRA of perfluoroalkyl iodide onto terminal alkenes.

Finally, we modified the pleuromutilin by the additional reaction of perfluoroalkyl iodide derivatives on the C19–C20 alkene. The atom-transfer radical addition of iodoperfluoralkanes (R_f_I) onto olefins can be very effectively activated by organocatalysts, under irradiation with visible or UV light [25,35]. A key step of the mechanism is the initiation involving radical R_f_ generation, which then enters the radical propagation chain and adds to the alkene (Scheme 5). In the radical chain process, the addition of R_f_ occurs to the terminal carbon in a regioselective way, producing a carbon centered radical which stabilizes by the abstraction of an iodine, to give the iodoperfluoralkylated product in the form of a diastereoisomeric mixture (Scheme 5B). We performed the reactions in MeOH using benzophenone (BP) as the catalyst under irradiation with UV-light at 365 nm. Under these conditions, the generation of the R_f_ radical mainly occurs through the reaction of MeOH with the excited state BP, producing a CH_2_OH radical which then abstracts an iodine from R_f_I. The R_f_ radical could also be generated through a direct energy transfer from the BP triplet excited state (3BP*) to R_f_I. The direct photolysis of R_f_I upon irradiation at 365 nm represents a minor pathway of initiation (Scheme 5A) [25].

**Scheme 6:**
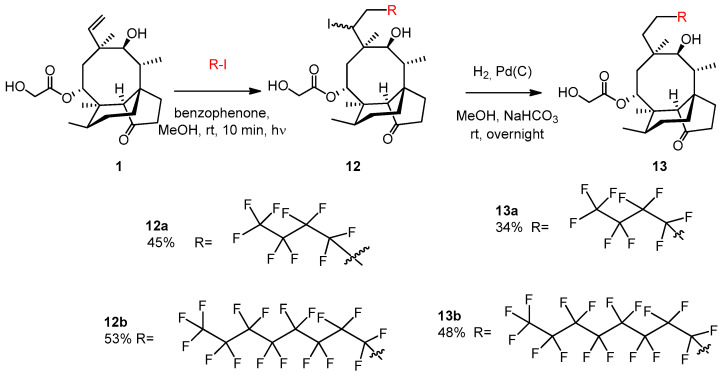
Modification of pleuromutilin with perfluoroalkylated side-chains.

The light-promoted atom-transfer radical addition of the commercially available perfluorobutyl iodide and perfluorooctyl iodide onto pleuromutilin was conducted in MeOH, at room temperature, using a BP catalyst under argon. The reaction resulted in the expected iodoperfluooralkylated **12a** and **12b** as mixtures of diastereoisomers in moderate yields. A subsequent catalytic hydrogenation of the C19-iodo derivatives gave the final products **13a** and **13b** with 34% and 48% yields, respectively (Scheme 6).

We have examined the antibacterial properties of the novel semisynthetic compounds on a panel of bacteria (Table 1). The 2-hydroxyethyl (**10j**), *N*-acetyl-l-cysteine (**10k**) and deprotected sialic acid (**11h**) derivatives were found to be potent antimicrobial compounds against biofilm-producing *S. epidermidis* and were as active against *Enterococcus faecium* VanA isolated from drain as the parent pleuromutilin. Moreover, these compounds showed excellent antibacterial properties against *MRSA* strain. Compound **10k** provided excellent activity against *Bacillus subtilis.* Unfortunately, the other semisynthetic derivatives proved to be inactive, and neither the fluorophilic nor lipophilic modifications resulted in effective compounds (MIC > 256 μg/mL).

**Scheme 7:**
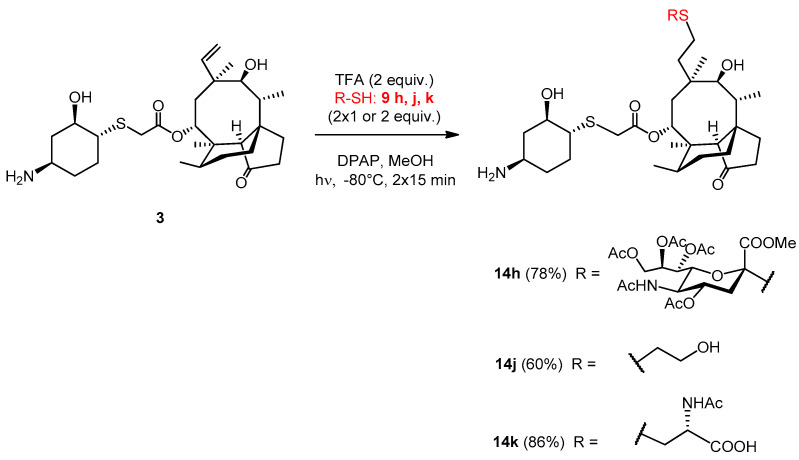
Hydrothiolation of lefamulin at position C19–C20.

Based on the promising antibacterial activity of pleuromutilin derivatives **10j**, **10k** and **11h**, modification of lefamulin by the thiol-ene coupling reaction was also performed. Lefamulin **3** was reacted with 2-mercaptoethanol **9j**, *N*-acetyl-l-cysteine **9k**, and the 5-*N*Ac-2-thio-α-d-neuraminic acid derivative **9h** (Scheme 7). In order to avoid deprotonation of the thiol reactants by the basic primary amino group, lefamulin was first converted into trifluoroacetate salt by treatment of trifluoroacetic acid, and the additional reactions were carried out under cooling, with excess amounts of thiols. Finally, lefamulin derivatives were isolated with good yields.

**Scheme 8:**
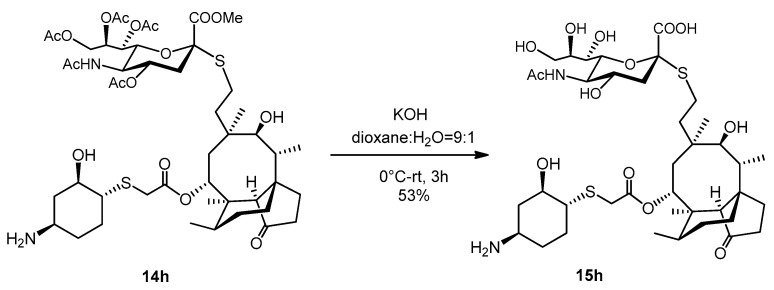
Deprotection of thiosialylated lefamulin derivative **14h**.

The acetyl protecting groups and methyl ester group of the sialic acid derivative **14h** were removed by KOH to result in the sialic acid modified lefamulin **15h** (Scheme 8).

The antibacterial properties of the novel semisynthetic lefamulin compounds were tested on the same panel of bacteria (Table 1) than in the case of pleuromutilin. The 2-hydroxyethyl (**14j**) derivative was found to show potent antimicrobial activity against VanA resistant *Enterococcus faecium* strains isolated from urine, blood culture, drain and wound. Although the 50S ribosomal subunit occurs only in prokaryotes, antibiotics that inhibit protein synthesis by binding to the 50S subunit—as pleuromutilin derivates do—may be severely toxic to eukaryotes [36]. Therefore, it is essential to perform in vitro and in vivo toxicological studies of the active derivatives in the future.

## 4. Conclusions

The modification of pleuromutilin with the addition of a series of thiols provided the development of three novel semisynthetic antibiotics effective against *B. subtilis*, sensitive and resistant staphylococci and resistant enterococci having vanA and vanB genes. The 2-mercaptoethanol, *N*-Ac-l-cystein and 2-thio-sialic acid addition could improve the antimicrobial activity of the pleuromutilin. However, neither further hydrophilic (carbohydrate side-chains) nor fluorophilic and alkyl- and aryl-type lipophilic modification resulted in effective compounds. The successful modifications were introduced to lefamulin, using photoinitiated hydrotiolation, which resulted in an ethylthio-containing novel semisynthetic lefamulin, with excellent activity against VanA resistant *Enterococcus faecium* strains. Considering the simple and efficient way to synthesize semisynthetic mutilins by the thiol-ene coupling reaction, we can conclude that we found effective semisynthetic mutilin-type antibiotics with promising antimicrobial effects.

## Figures and Tables

**Table 1:** Antibacterial effect of semisynthetic pleuromutilins based on Minimal Inhibitory Concentration [28] of active compounds (MIC: mg/L) (ATCC: American Type Culture Collection, MRSA/MSSA: methicillin resistant/sensitive *S. aureus*, VanA and VanB: vancomycin resistant phenotypes.)

Bacterial Strains	1	10j	10k	11h	3	14j	14k	15h
*Bacillus subtilis* ATCC 6633	16	32	4	32	0.5	16	256	512
*Staphylococcus aureus* MSSA ATCC 29213	4	16	8	16	0.5	16	512	512
*Staphylococcus aureus* MRSA ATCC 33591	2	4	4	4	0.5	32	512	256
*Staphylococcus epidermidis* ATCC 35984 biofilm	1	2	0.5	2	0.5	16	512	128
*Staphylococcus epidermidis* mecA	4	16	8	16	0.5	16	512	256
*Enterococcus faecalis* ATCC 29212	64	256	32	256	64	64	512	512
*Enterococcus faecalis* 15376 VanA	128	256	64	128	1	16	512	256
*Enterococcus faecium* VanA 38276 urine	128	128	128	128	1	2	256	256
*Enterococcus faecium* VanA 25192 bloodculture	128	128	128	128	2	2	512	256
*Enterococcus faecium* VanA 3452 drain	2	2	2	2	1	2	256	512
*Enterococcus faecium* VanA 24581 wound	128	128	128	128	1	1	512	512
*Enterococcus faecalis* ATCC 51299 VanB	128	256	32	256	64	64	256	256

## Data Availability

Data is contained within the article and Supplementary Materials.

## Supplementary Materials

The following materials are available online at https://www.mdpi.com/article/10.3390/pharmaceutics13122028/s1, Figure S1: ^1^H and ^13^C NMR spectrum (400 MHz, CDCl_3_) of compound **10a**. Figure S2: ^1^H and ^13^C NMR spectrum (400 MHz, MeOD) of compound **10b**. Figure S3: ^1^H and ^13^C NMR spectrum (400 MHz, MeOD) of compound **10c**. Figure S4: ^1^H and ^13^C NMR spectrum (400 MHz, CDCl_3_) of compound **10d**. Figure S5: ^1^H and ^13^C NMR spectrum (400 MHz, DMSO) of compound **10e**. Figure S6: ^1^H and ^13^C NMR spectrum (400 MHz, DMSO) of compound **10f**. Figure S7: ^1^H and ^13^C NMR spectrum (360 MHz, CDCl_3_) of compound **10g**. Figure S8: ^1^H and ^13^C NMR spectrum (400 MHz, CDCl_3_) of compound **10h**. Figure S9: ^1^H and ^13^C NMR spectrum (400 MHz, MeOD) of compound **10i**. Figure S10: ^1^H and ^13^C NMR spectrum (400 MHz, MeOD) of compound **10j**. Figure S11: ^1^H and ^13^C NMR spectrum (400 MHz, DMSO) of compound **10k**. Figure S12: ^1^H and ^13^C NMR spectrum (500 MHz, MeOD) of compound **10l**. Figure S13: ^1^H and ^13^C NMR spectrum (400 MHz, MeOD) of compound **10m**. Figure S14: ^1^H and ^13^C NMR spectrum (400 MHz, MeOD) of compound **10n**. Figure S15: ^1^H and ^13^C NMR spectrum (400 MHz, CDCl_3_) of compound **10o**. Figure S16: ^1^H and ^13^C NMR spectrum (400 MHz, CDCl_3_) of compound **10p**. Figure S17: ^1^H and ^13^C NMR spectrum (400 MHz, CDCl_3_) of compound **10q**. Figure S18: ^1^H and ^13^C NMR spectrum (400 MHz, MeOD) of compound **10r**. Figure S19: ^1^H and ^13^C NMR spectrum (400 MHz, MeOD) of compound **11g**. Figure S20: ^1^H and ^13^C NMR spectrum (400 MHz, DMSO) of compound **11h**. Figure S21: ^1^H and ^13^C NMR spectrum (400 MHz, MeOD) of compound **11l**. Figure S22: ^1^H and ^13^C NMR spectrum (400 MHz, CDCl_3_) of compound **13a**. Figure S23: ^1^H and ^13^C NMR spectrum (400 MHz, CDCl_3_) of compound **13b**. Figure S24: ^1^H and ^13^C NMR spectrum (400 MHz, DMSO) of compound **14h**. Figure S25: ^1^H and ^13^C NMR spectrum (400 MHz, DMSO) of compound **14j**. Figure S26: ^1^H and ^13^C NMR spectrum (400 MHz, DMSO) of compound **14k**. Figure S27: ^1^H and ^13^C NMR spectrum (400 MHz, DMSO) of compound **15h**. Figure S28: The experimental setup for carrying out hydrothiolation reactions at low temperature. Figure S29: ESI-MS spectrum of compound **10j**. Figure S30: MALDI-MS spectrum of compound **10k**. Figure S31: Calibrated MALDI-MS spectrum of compound **10k**. Figure S32: MALDI-MS spectrum of compound **11h**. Figure S33: Calibrated MALDI-MS spectrum of compound **11h**. Figure S34: ESI-MS spectrum of compound **14j**. Figure S35: MALDI-MS spectrum of compound **14k**. Figure S36: Calibrated MALDI-MS spectrum of compound **14k**. Figure S37: ESI-MS spectrum of compound **15h**.
