# Supplementary material for: The Very First Modification of Pleuromutilin and Lefamulin by Photoinitiated Radical Addition Reactions—Synthesis and Antibacterial Studies
Source: Pharmaceutics. 2021 Nov 28;13(12):2028. doi: 10.3390/pharmaceutics13122028 (PMC8704873; doi:10.3390/pharmaceutics13122028)
Supplement: Supplementary file 1 [file pharmaceutics-13-02028-s001.zip › pharmaceutics-1447183-supplementary.pdf]

pharmaceutics

# Supplementary Materials: The Very First Modification of Pleuromutilin and Lefamulin by Photoinitiated Radical Addition Reactions—Synthesis and Antibacterial Studies

**Son Thai Le [1,2], Dávid Páll [1], Erzsébet Rőth [1], Tuyen Tran [1], Nóra Debreczeni [1,3,4], Miklós Bege [1,4,5], Ilona Bereczki [1], Eszter Ostorházi [6], Márton Milánkovits [6], Pál Herczegh [1], Anikó Borbás [1,*] and Magdolna Csávás [5,*]**

1 Department of Pharmaceutical Chemistry, University of Debrecen, Egyetem tér 1, H-4032 Debrecen, Hungary; le.thai.son@pharm.unideb.hu (S.T.L.); Azgard-Gwendir@hotmail.com (D.P.); rothnej@gmail.com (E.R.); tuyentran1211@gmail.com (T.T.); debreczeni.nora@science.unideb.hu (N.D.); bege.miklos@euipar.unideb.hu (M.B.); bereczki.ilona@pharm.unideb.hu (I.B.); herczegh.pal@pharm.unideb.hu (P.H.)
2 Doctoral School of Pharmaceutical Sciences, University of Debrecen, Egyetem tér 1, H-4032 Debrecen, Hungary
3 Doctoral School of Chemistry, University of Debrecen, Egyetem tér 1, H-4032 Debrecen, Hungary
4 Institute of Healthcare Industry, University of Debrecen, Nagyerdei körút 98, H-4032 Debrecen, Hungary
5 Loránd Eötvös Research Network, Molecular Recognition and Interaction Research Group, University of Debrecen, Egyetem tér 1, H-4032 Debrecen, Hungary
6 Department of Medical Microbiology, Semmelweis University, Mária u. 41, H-1085 Budapest, Hungary; ostorhazi.eszter@med.semmelweis-univ.hu (E.O.); milankovits.marton@gmail.com (M.M.)
* Correspondence: borbas.aniko@pharm.unideb.hu (A.B.); csavas.magdolna@science.unideb.hu (M.C.); Tel.: +36-52-512-900 (M. Cs. and A.B.)

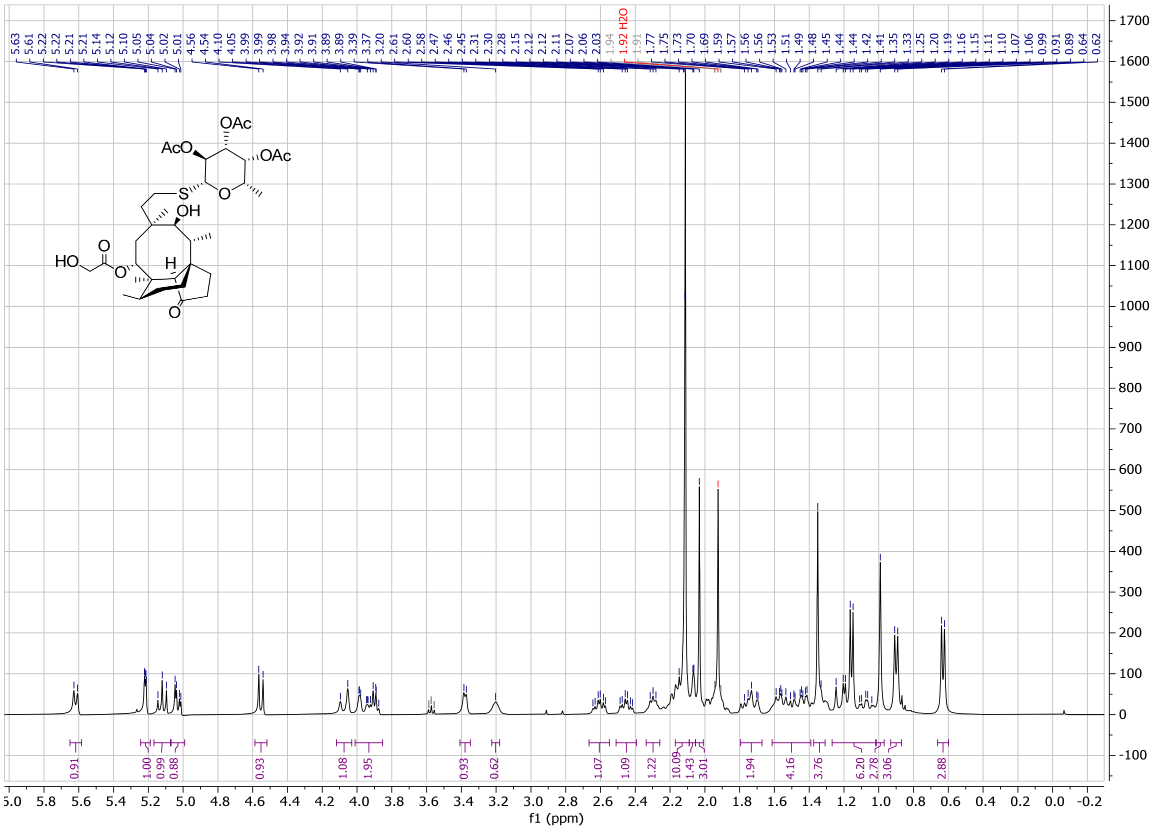

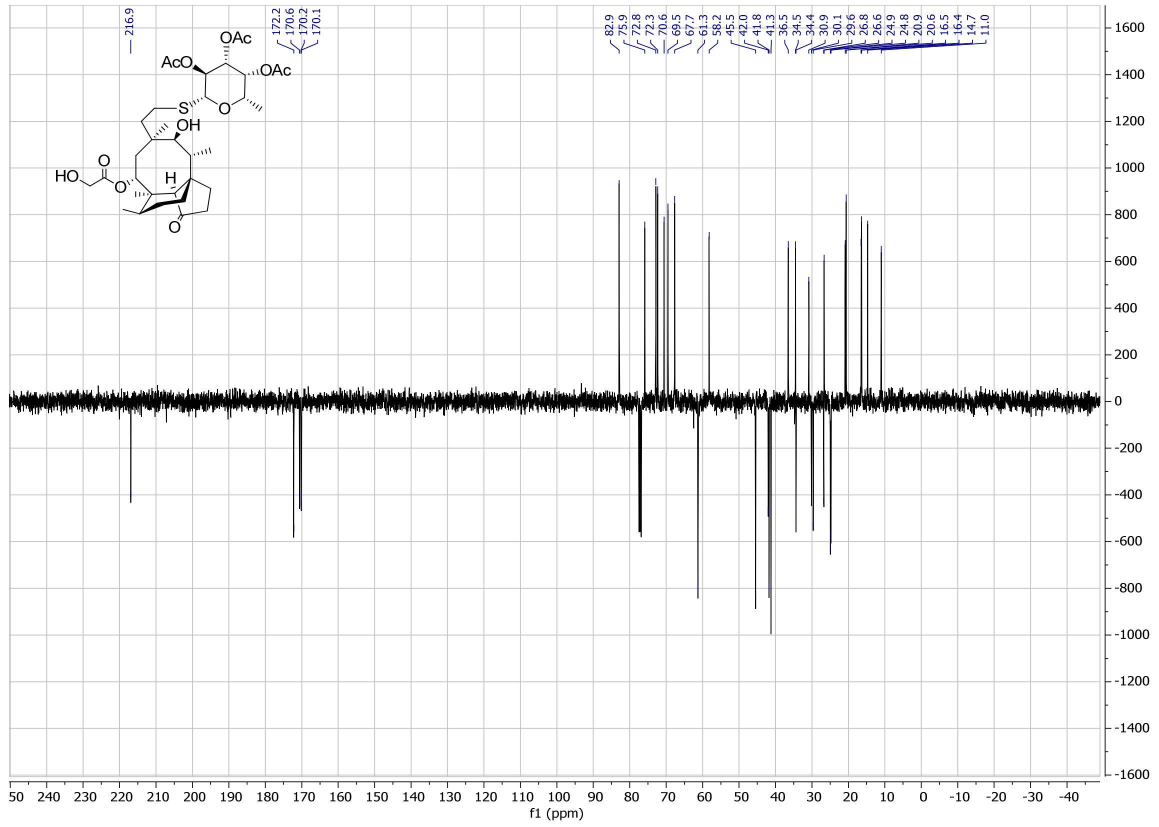

**Figure S1.** $^{1}H$ and $^{13}C$ NMR spectrum (400 MHz, $CDCl_3$) of compound **10a**.

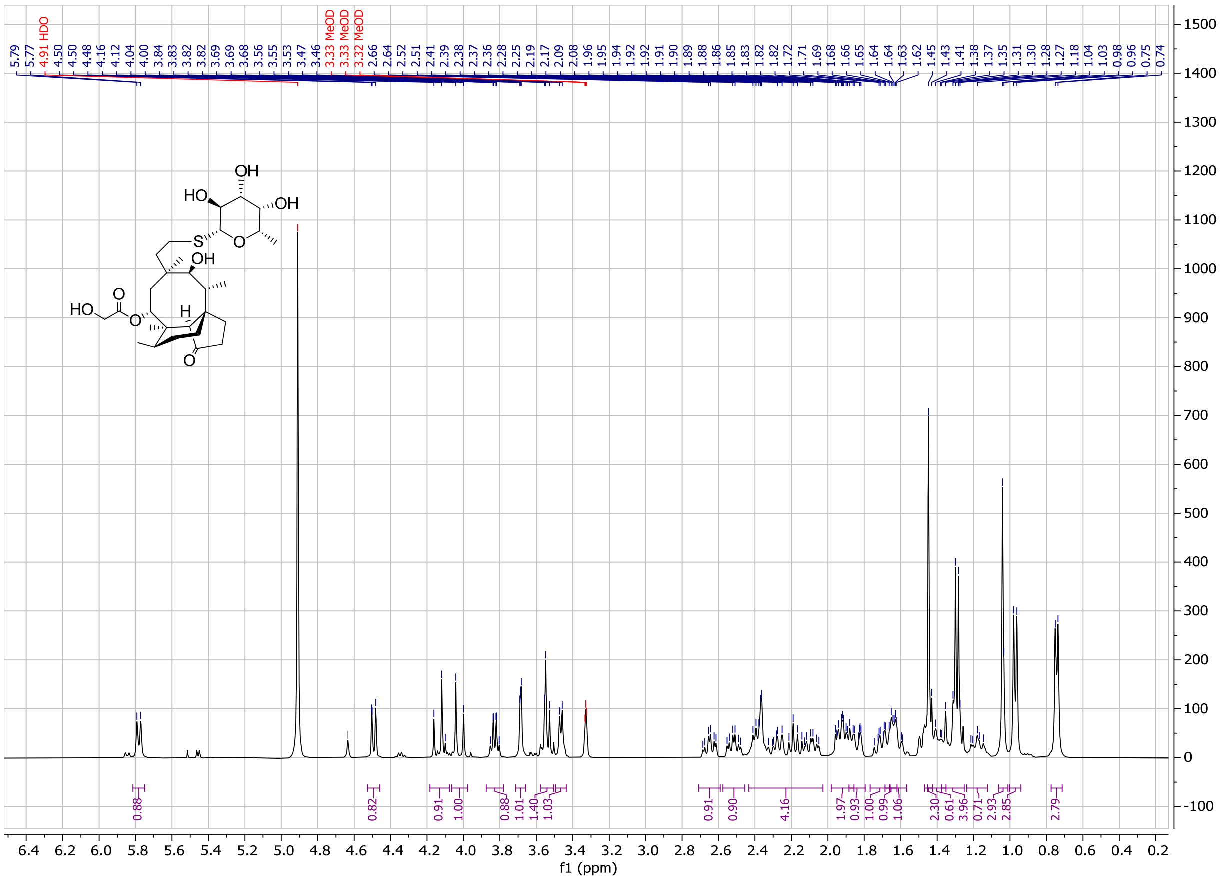

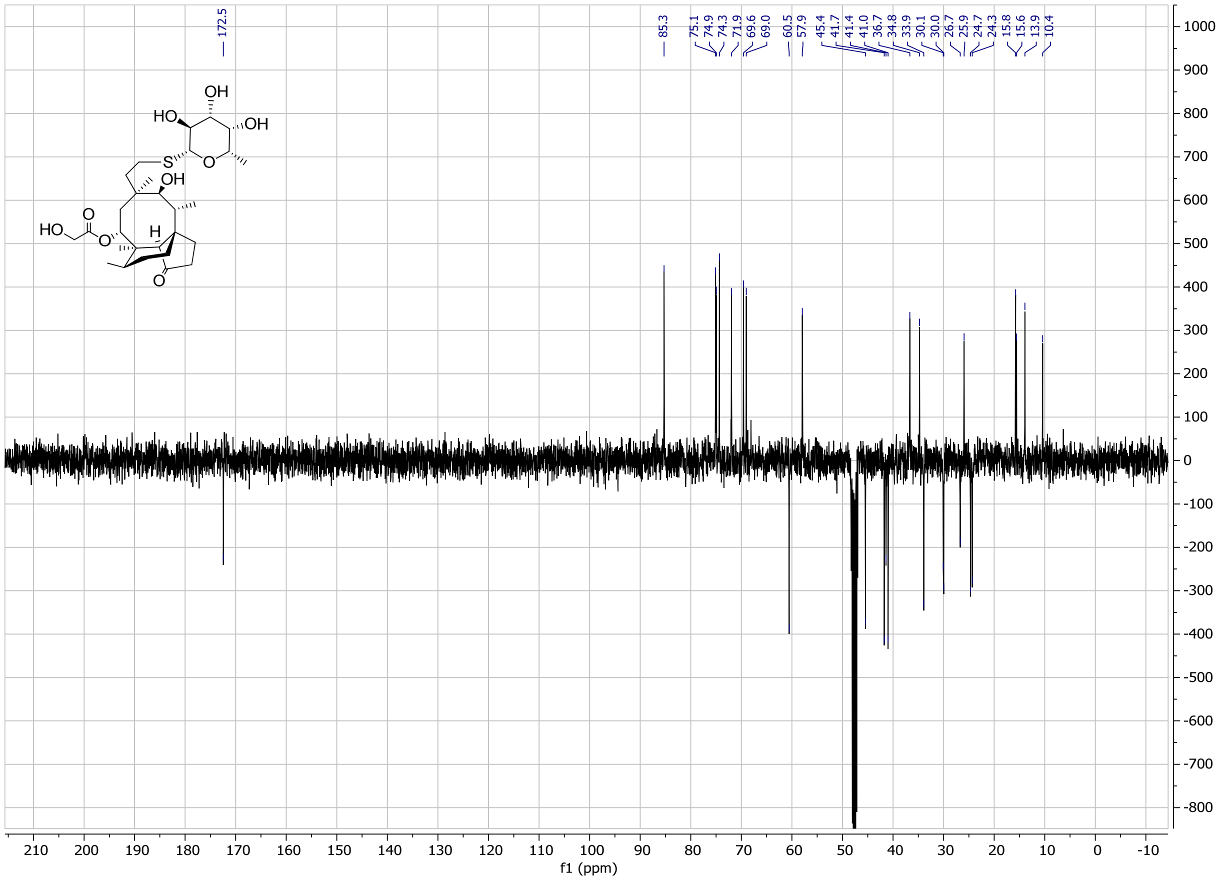

**Figure S2.** $^{1}$H and $^{13}$C NMR spectrum (400 MHz, MeOD) of compound **10b**.

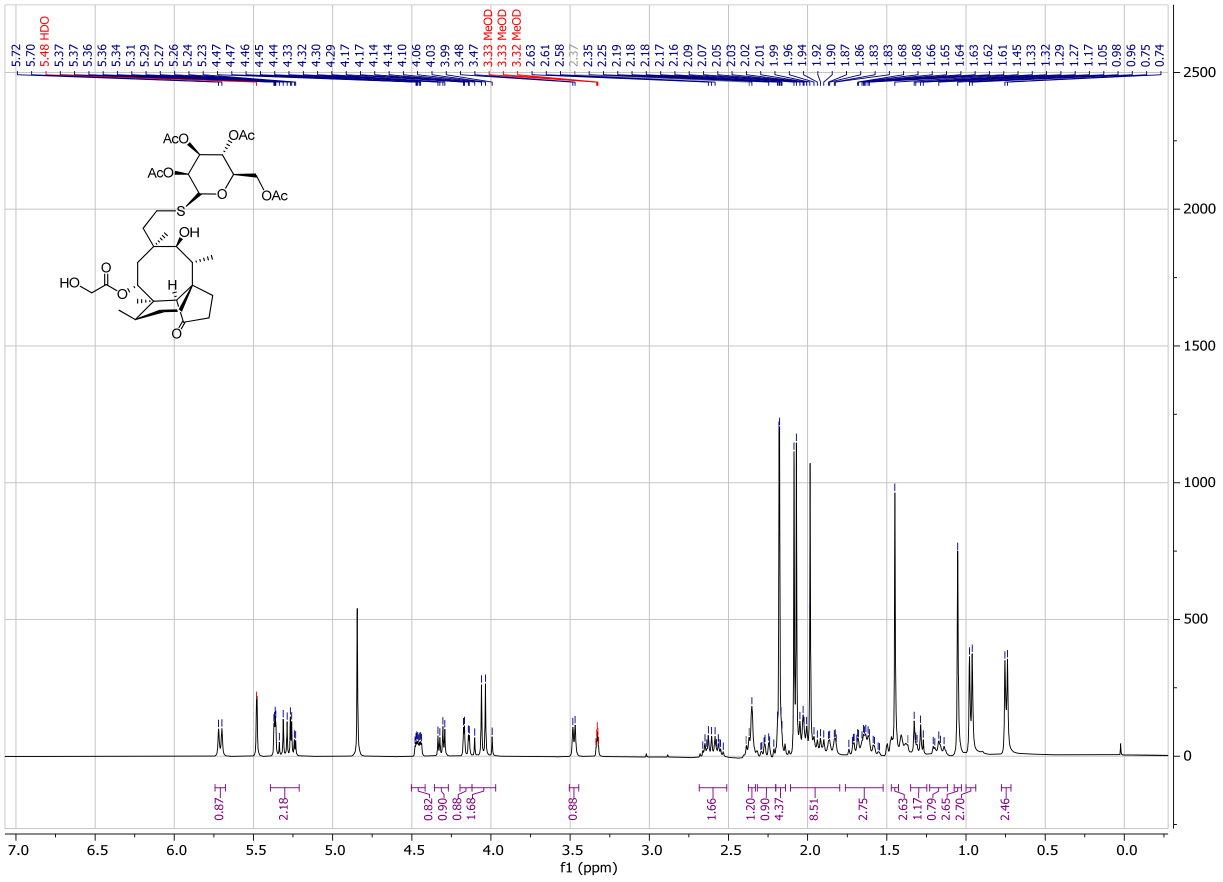

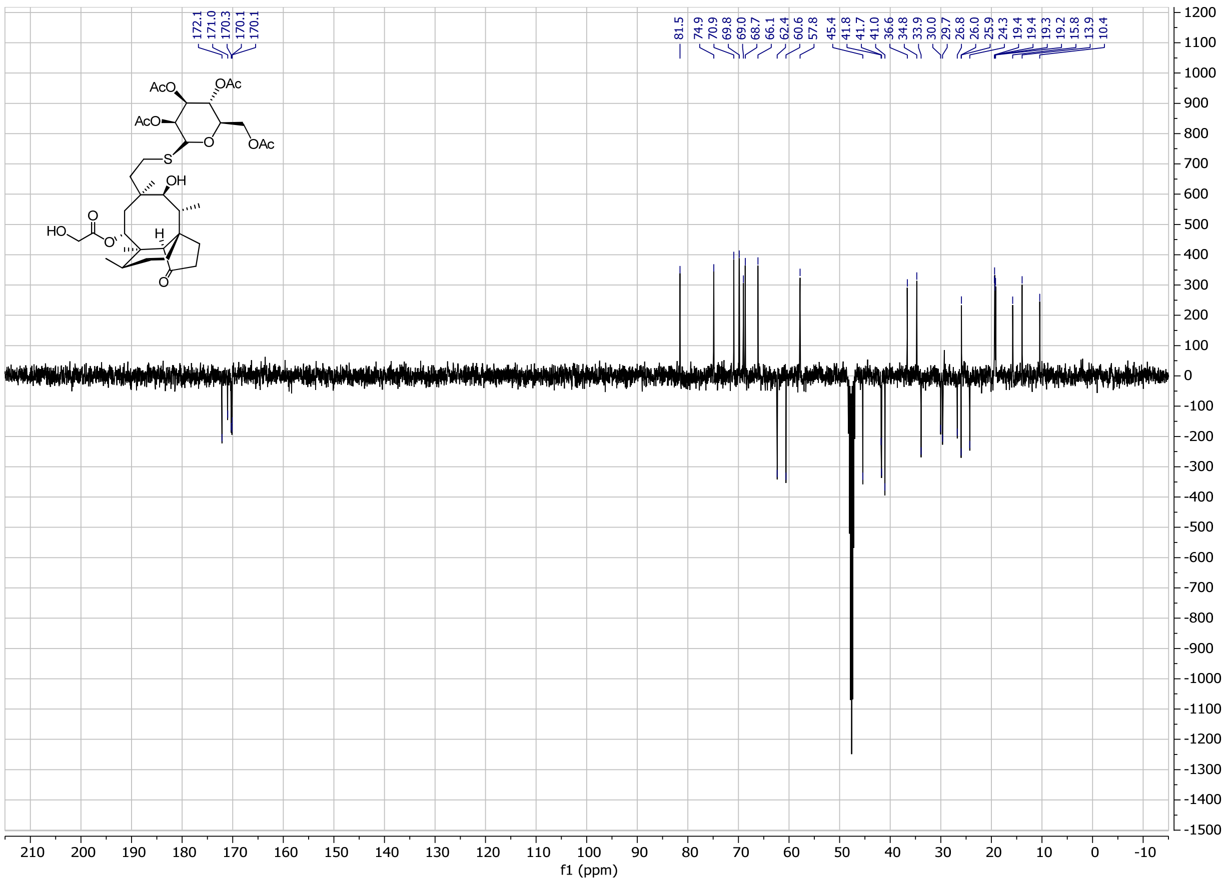

**Figure S3.** $^{1}$H and $^{13}$C NMR spectrum (400 MHz, MeOD) of compound **10c**.

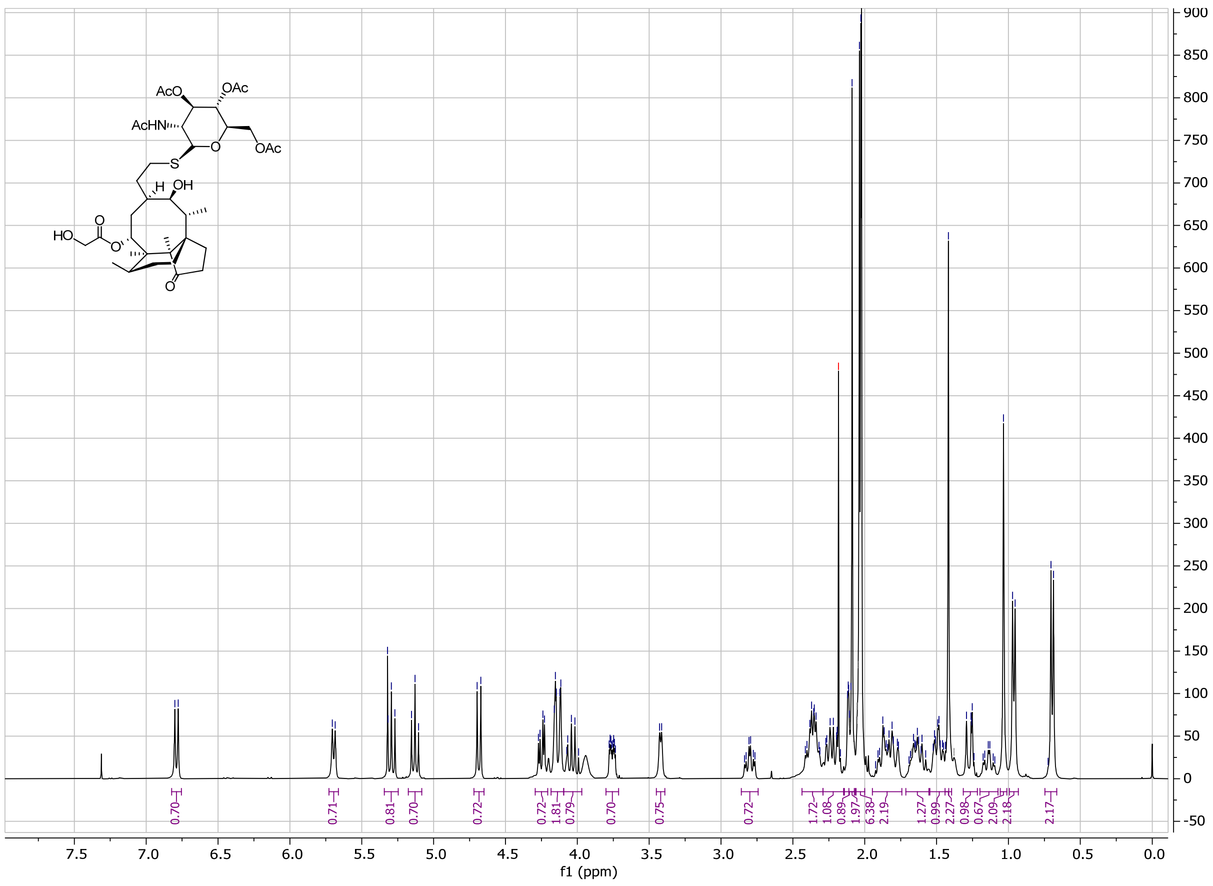

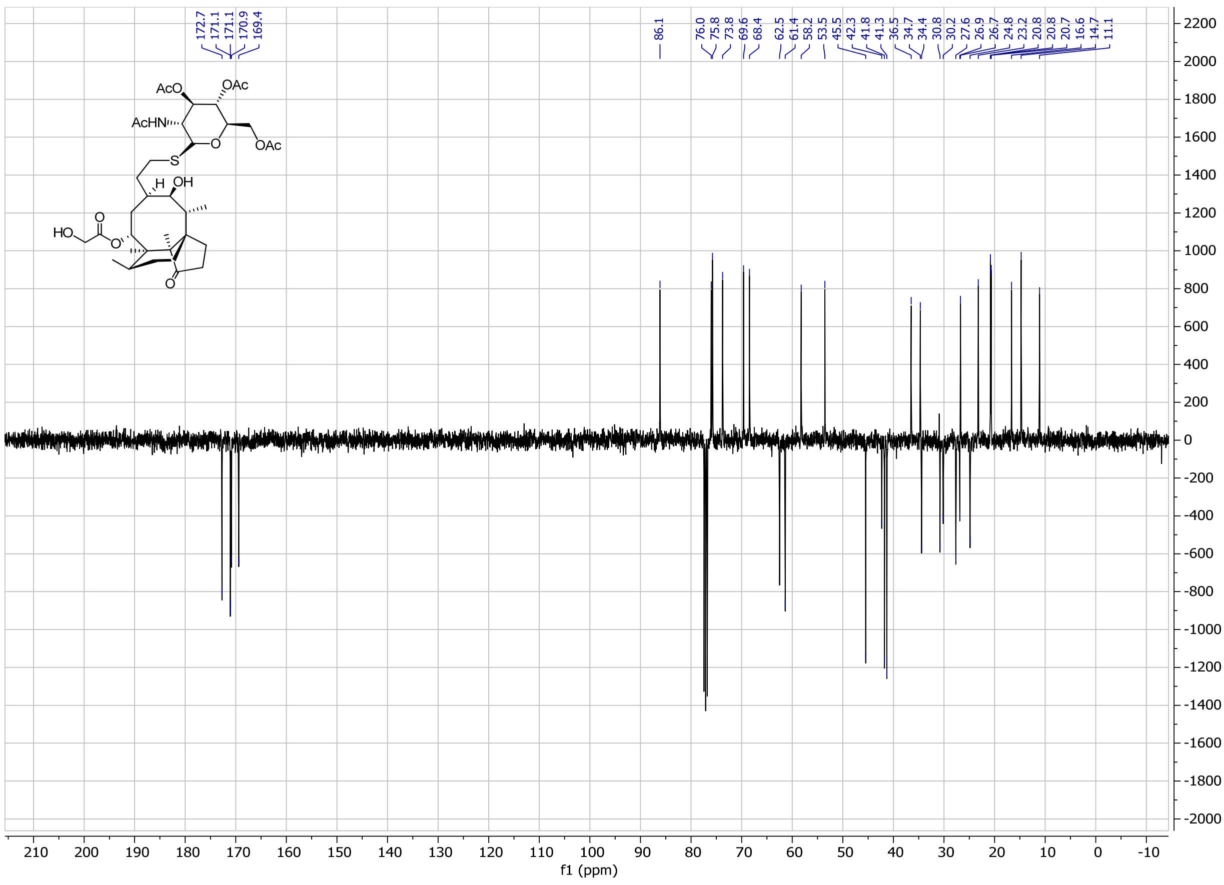

**Figure S4.** $^{1}H$ and $^{13}C$ NMR spectrum (400 MHz, $CDCl_3$) of compound **10d**.

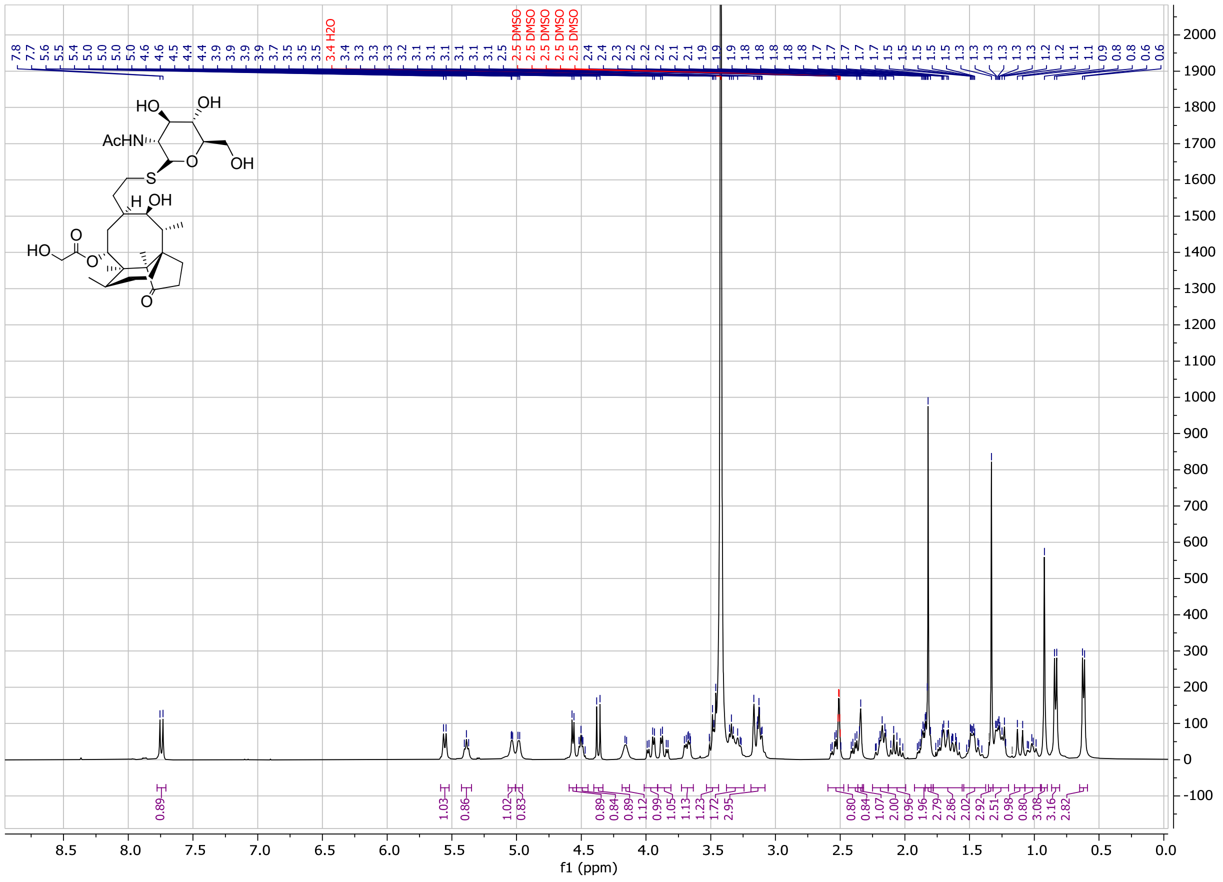

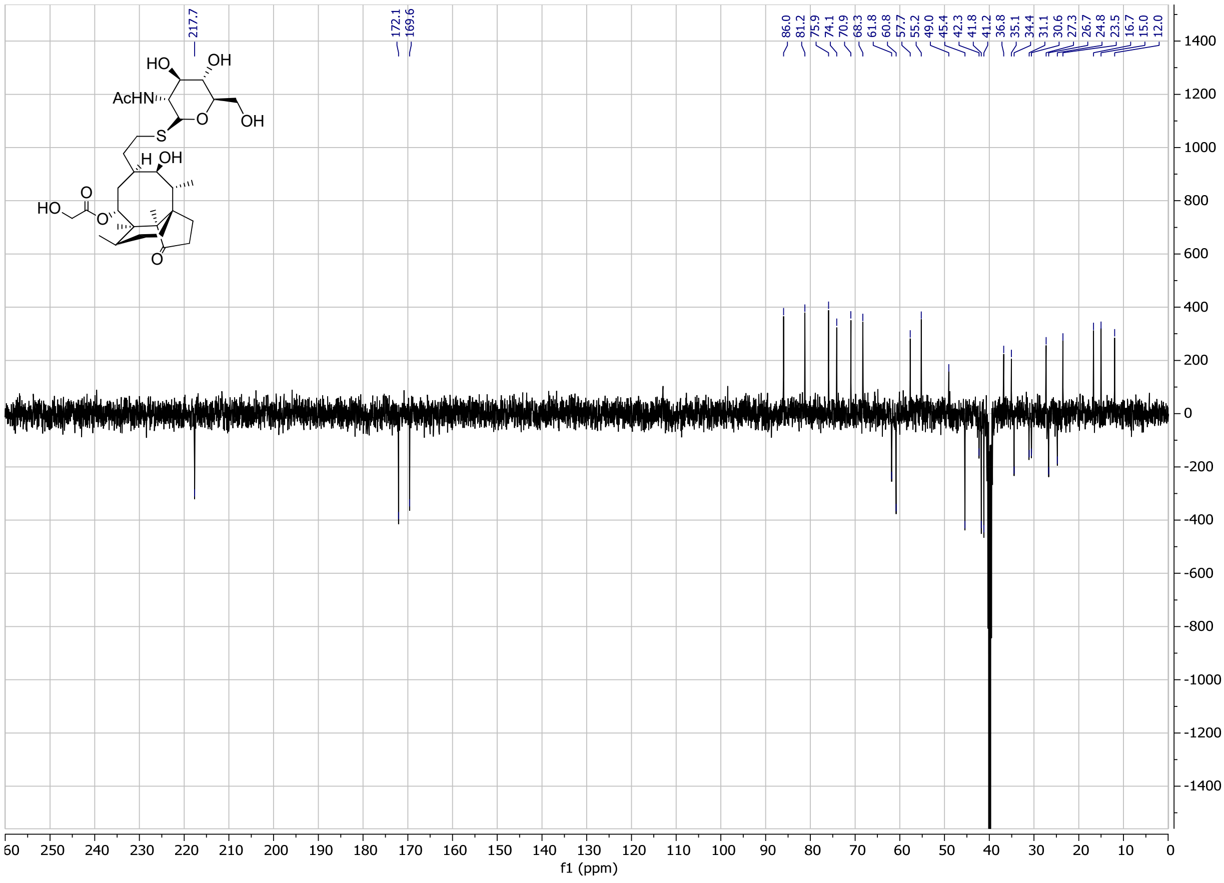

**Figure S5.** $^1$H and $^{13}$C NMR spectrum (400 MHz, DMSO) of compound **10e**.

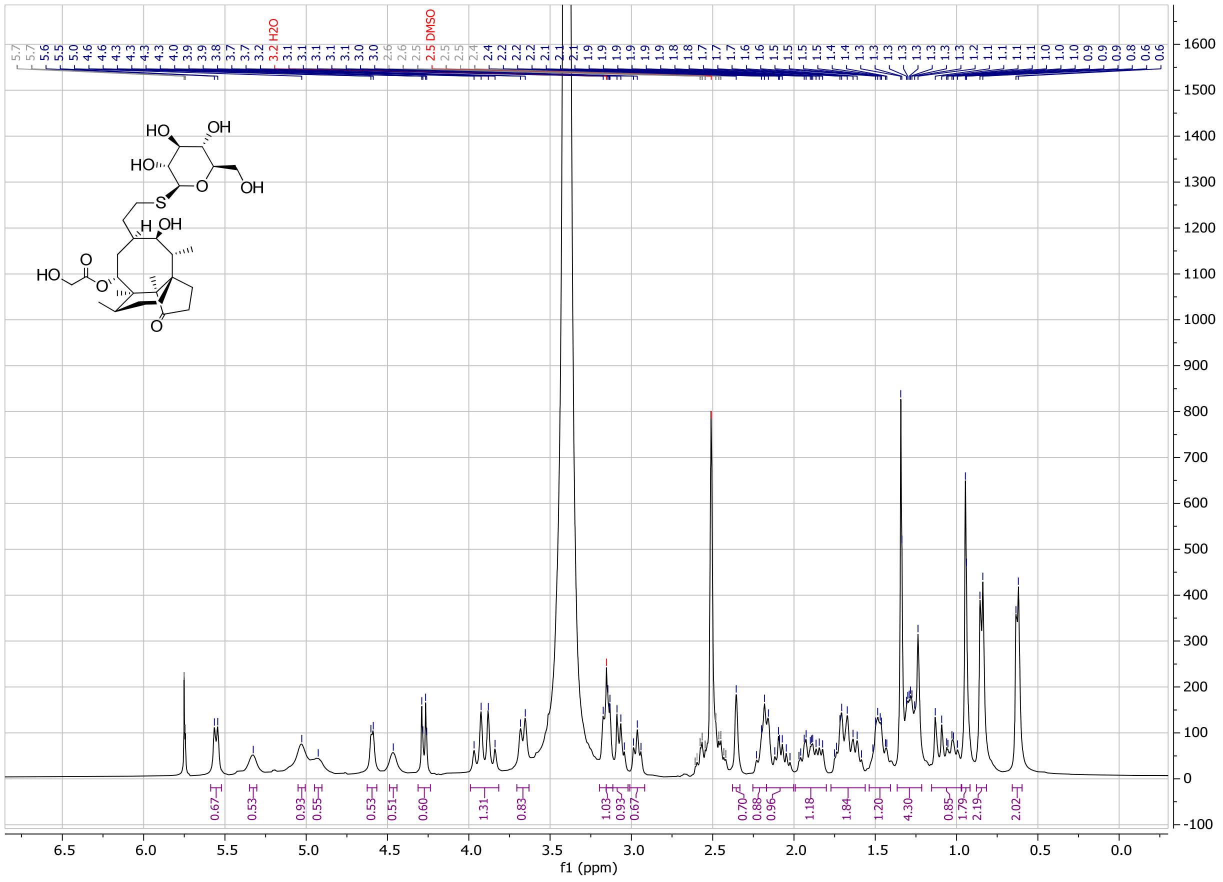

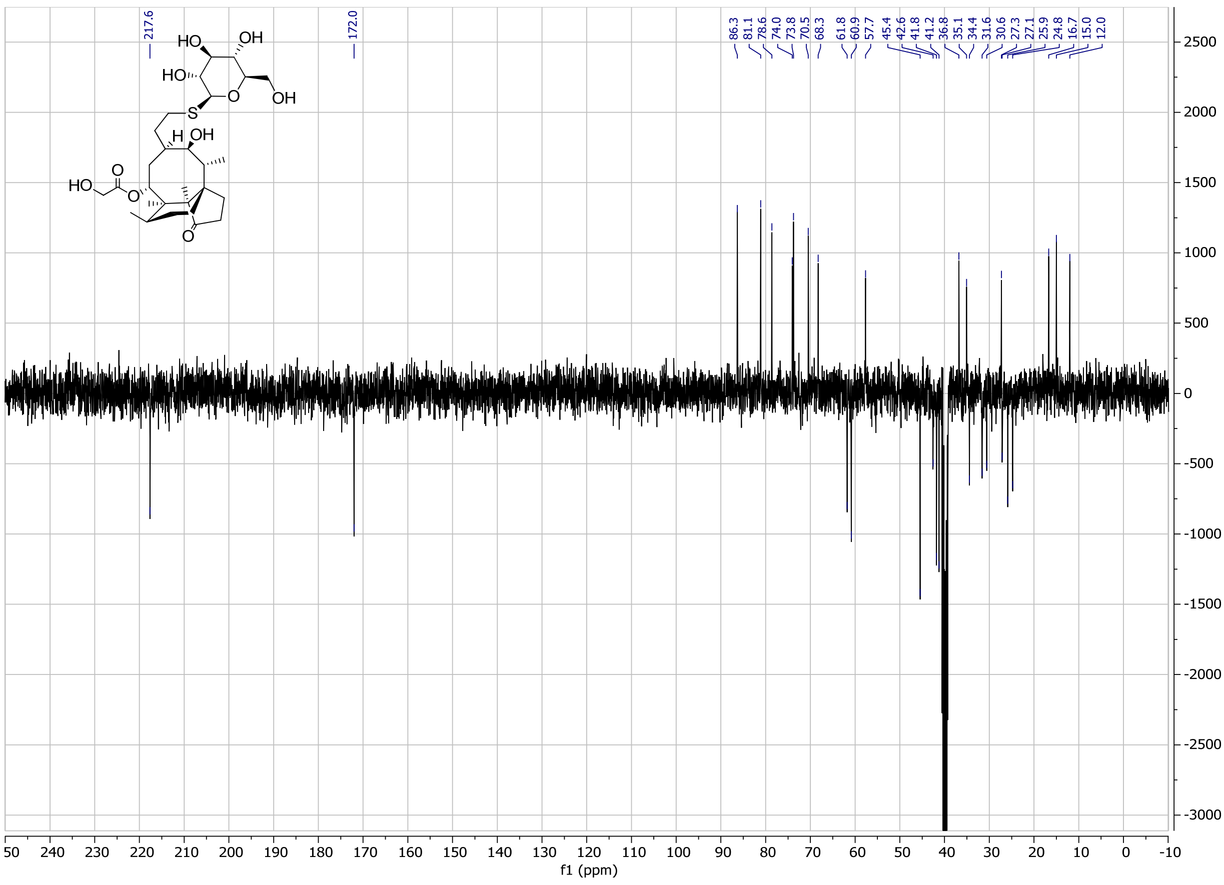

**Figure S6.** $^{1}H$ and $^{13}C$ NMR spectrum (400 MHz, DMSO) of compound **10f**.

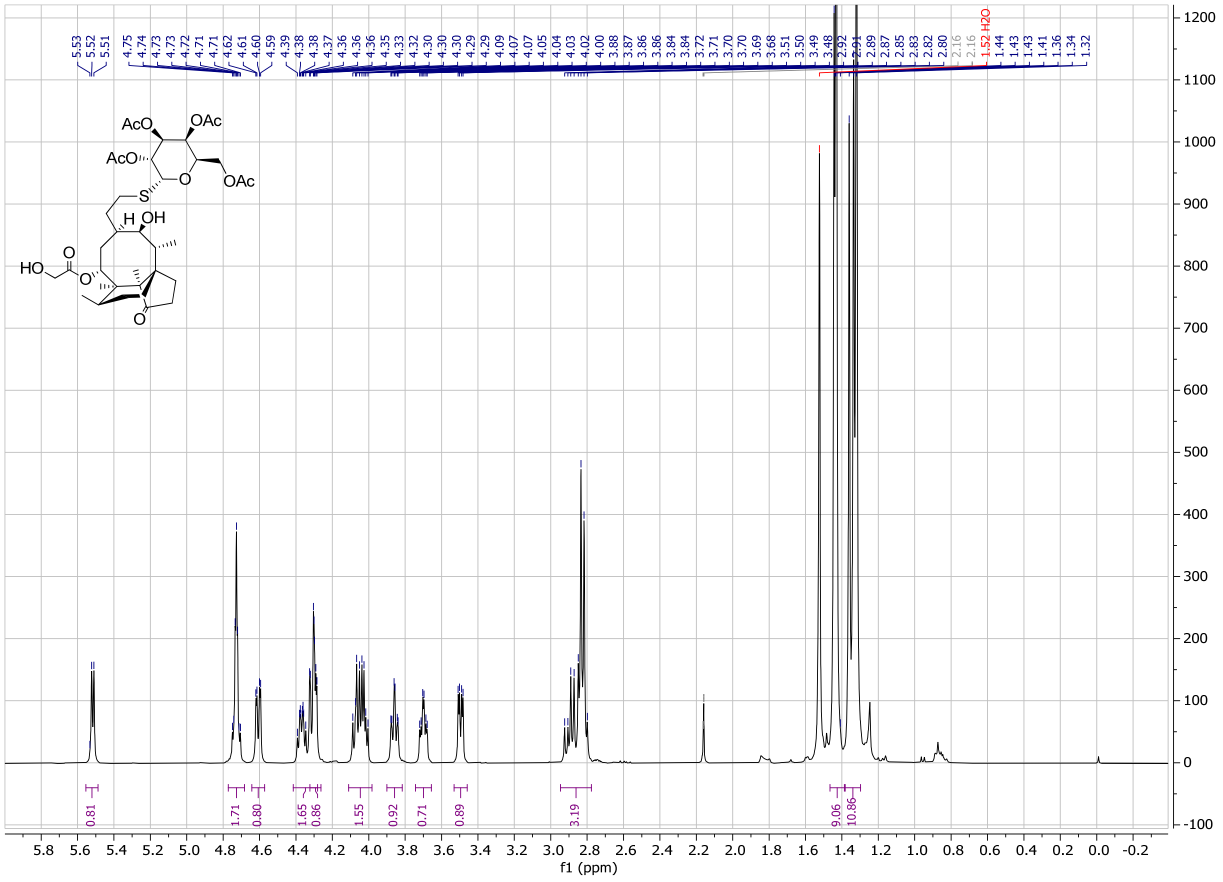

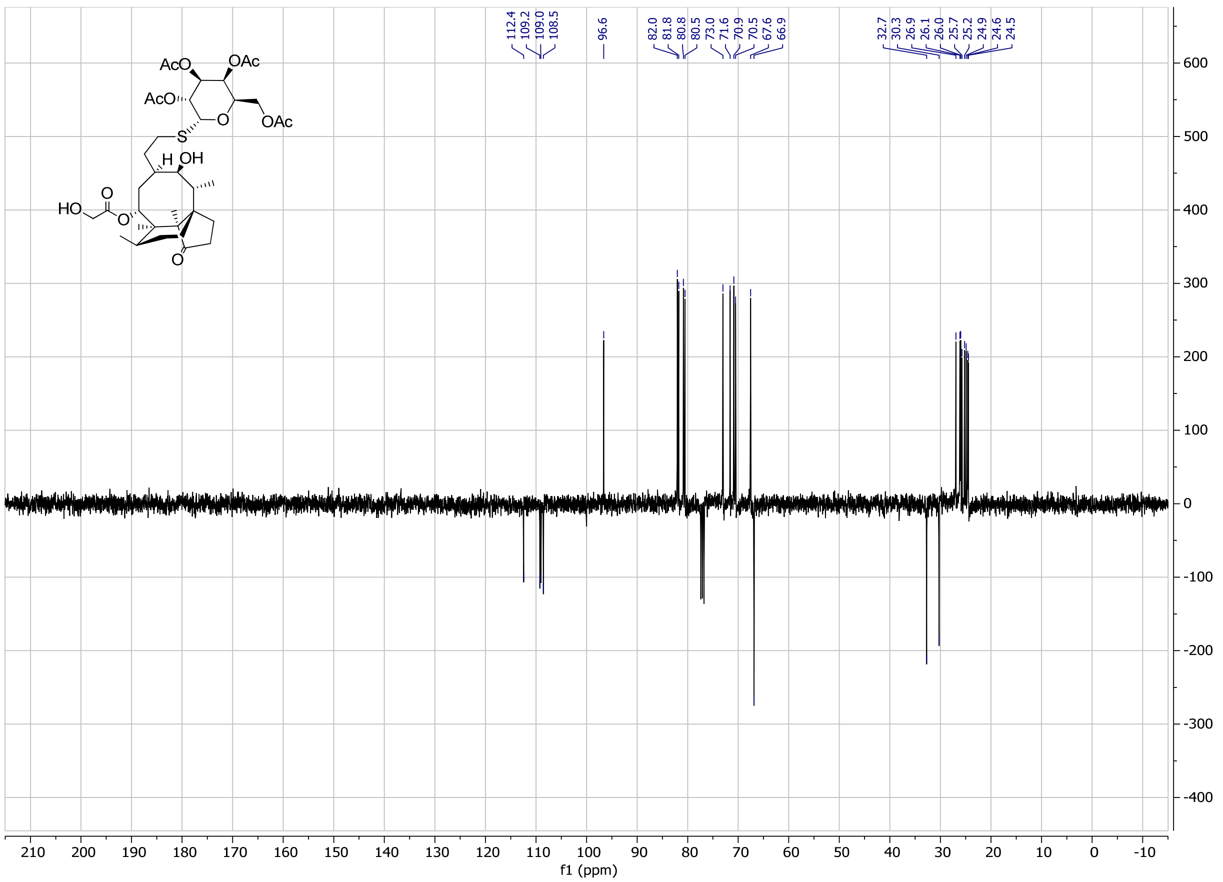

**Figure S7.** $^{1}$H and $^{13}$C NMR spectrum (360 MHz, CDCl3) of compound **10g**.

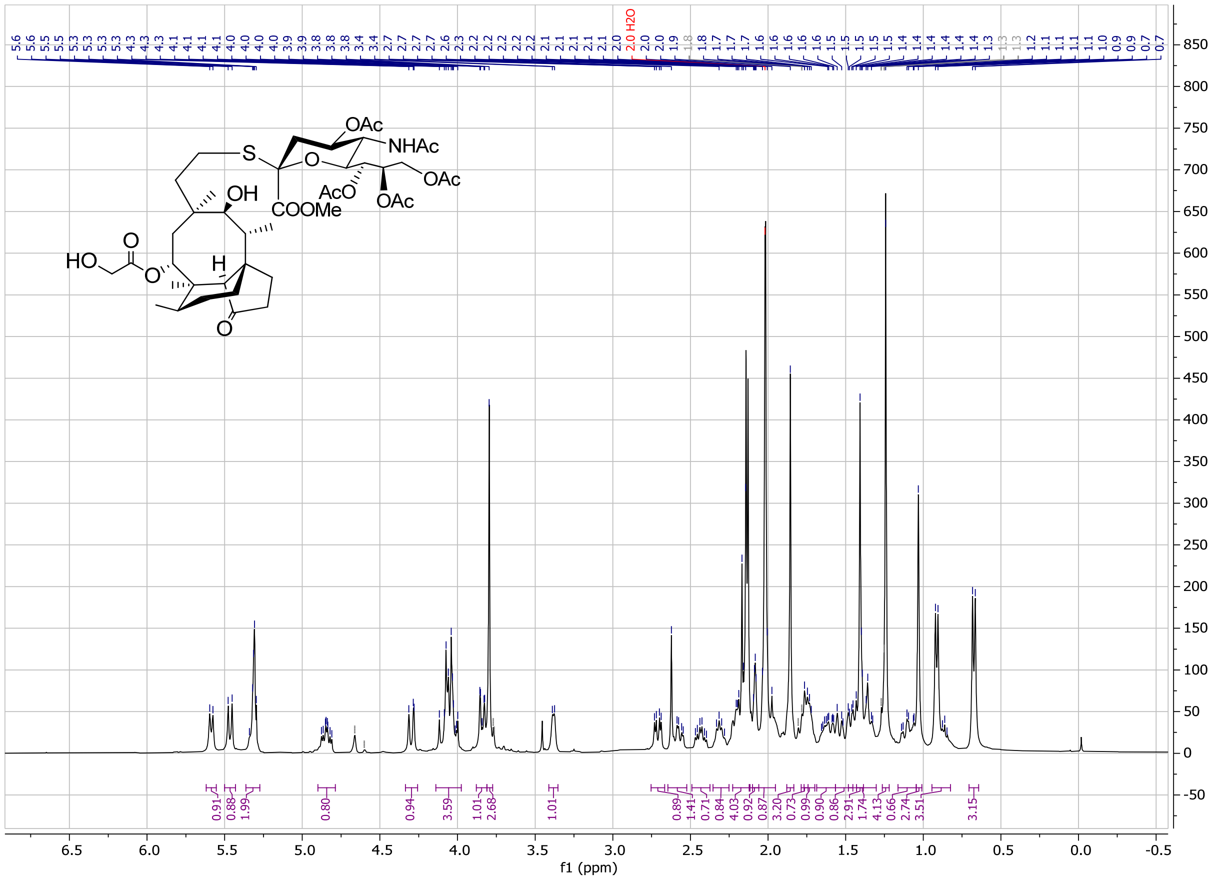


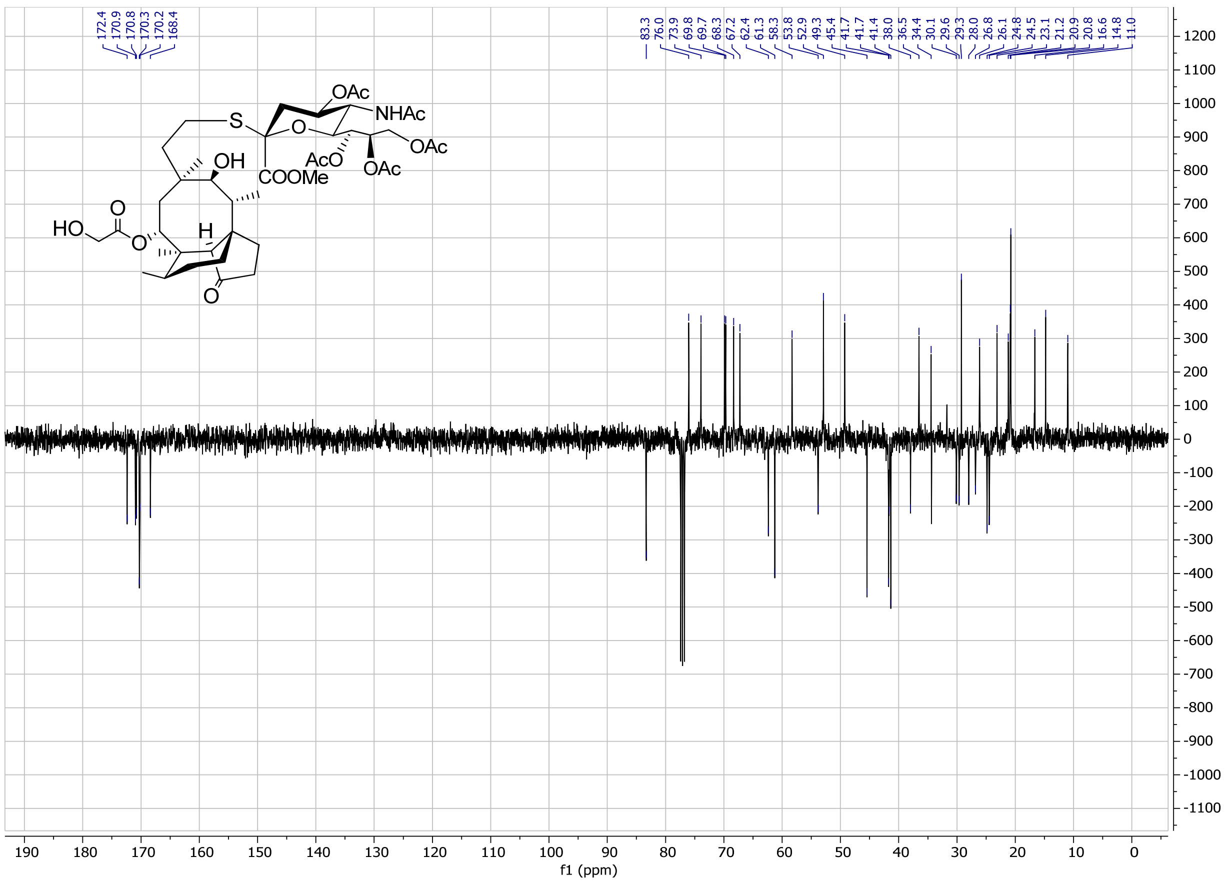


**Figure S8.** $^{1}H$ and $^{13}C$ NMR spectrum (400 MHz, $CDCl_3$) of compound **10h**.

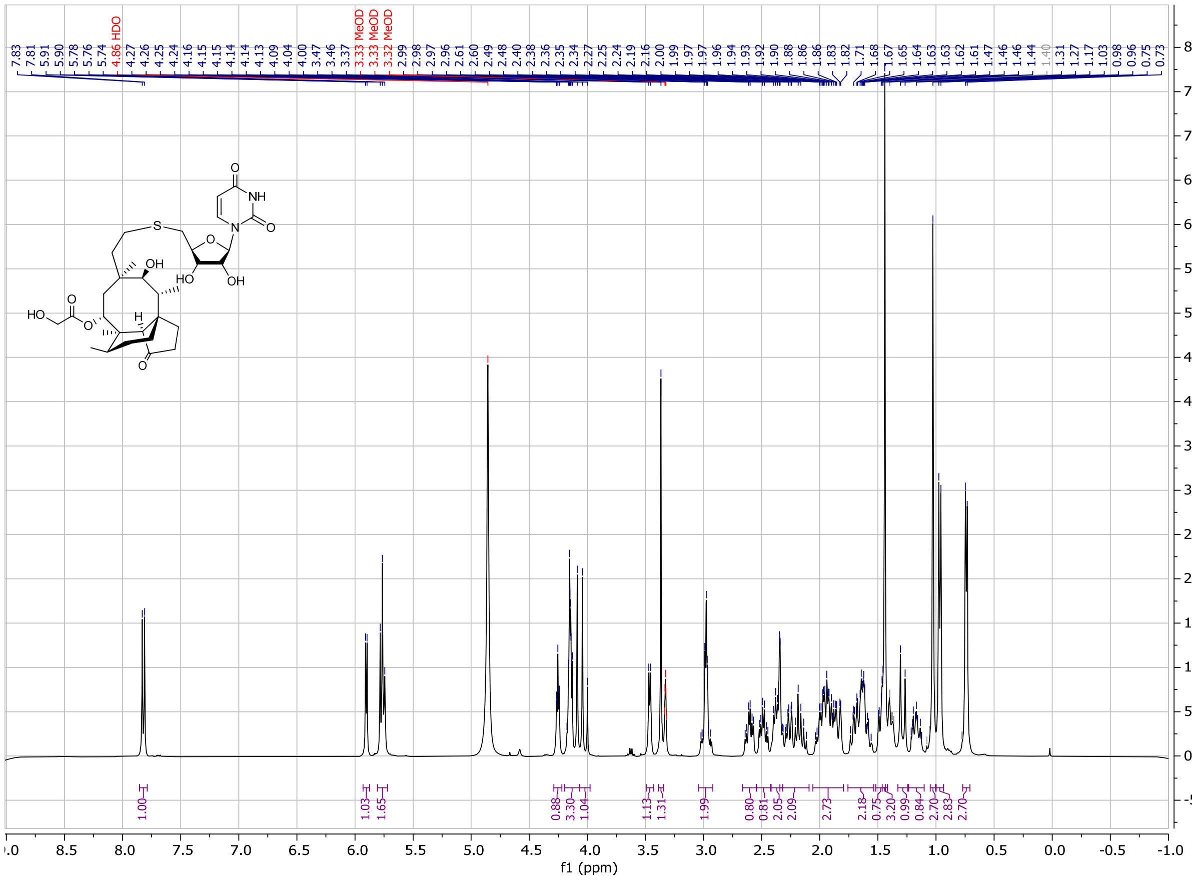

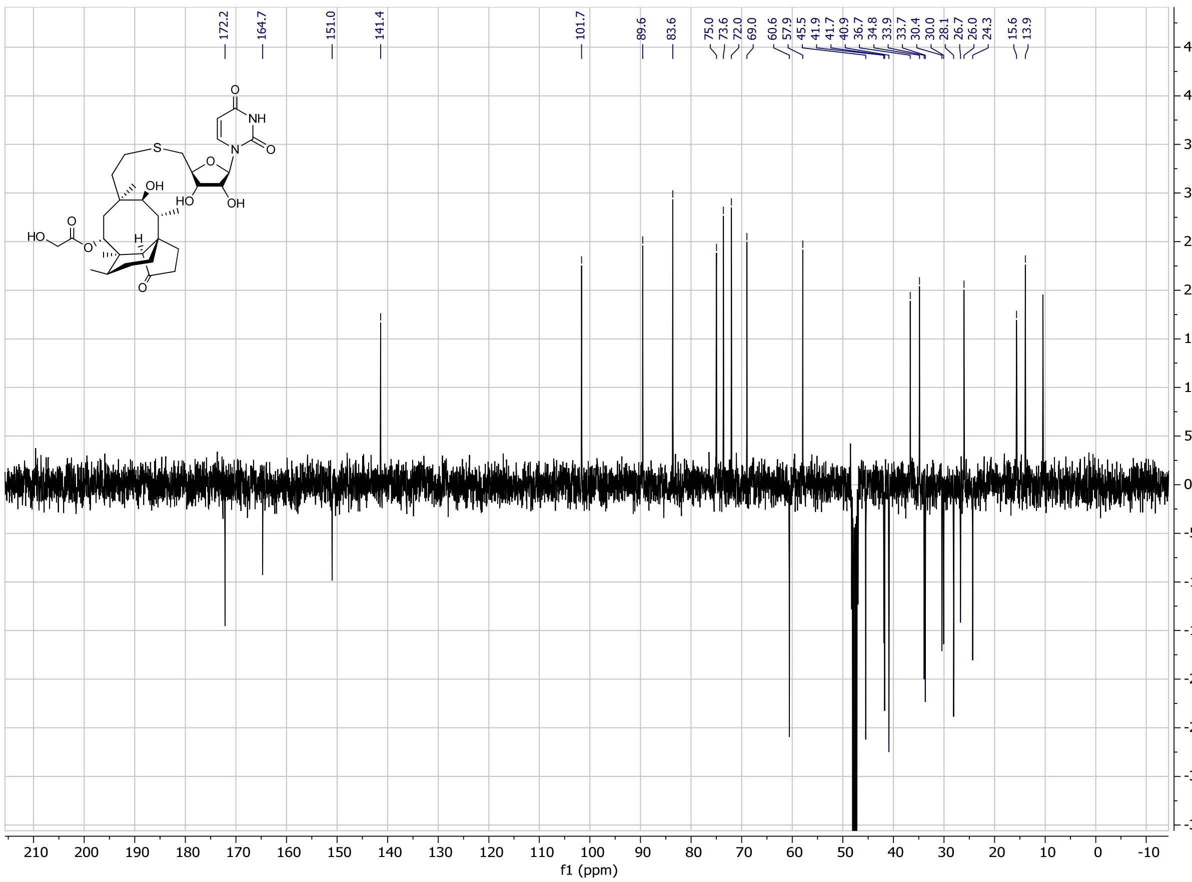

**Figure S9.** $^{1}H$ and $^{13}C$ NMR spectrum (400 MHz, MeOD) of compound **10i**.

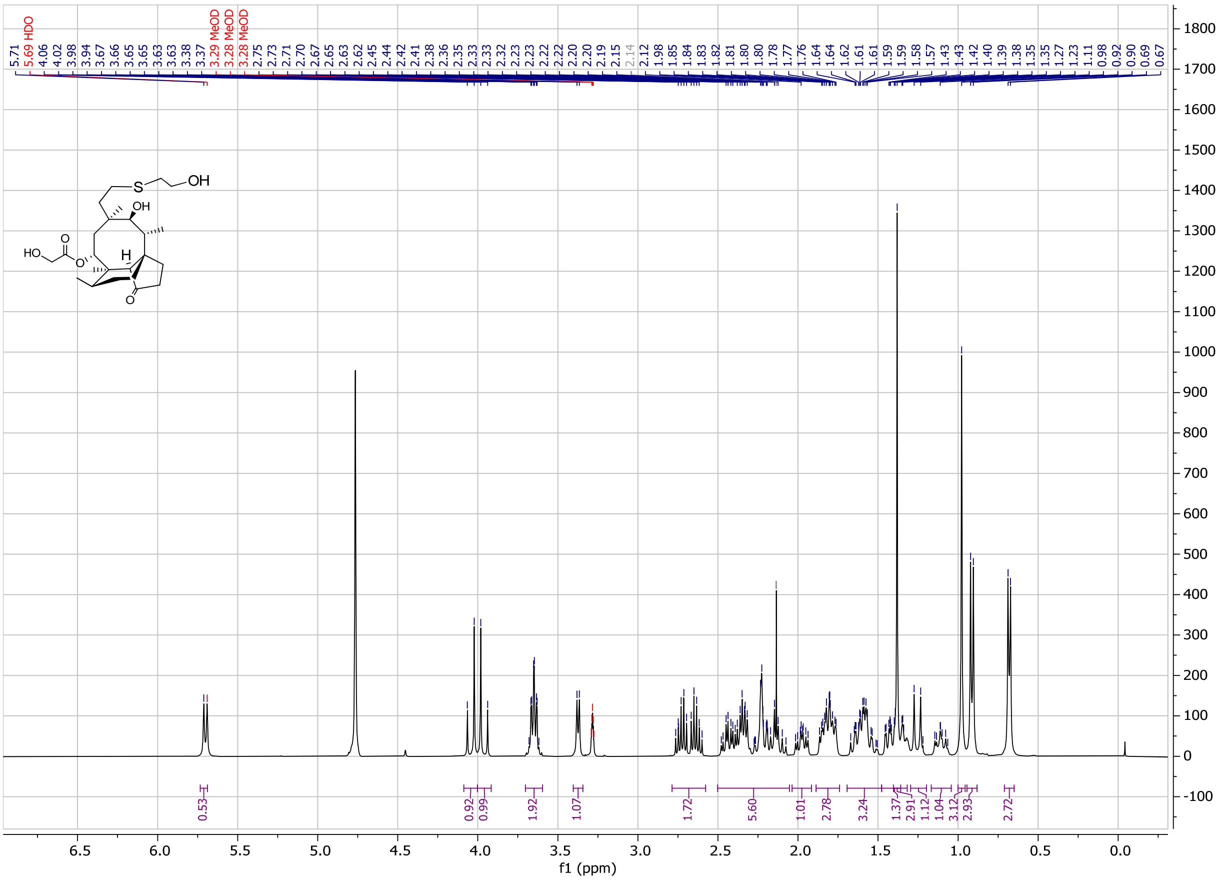


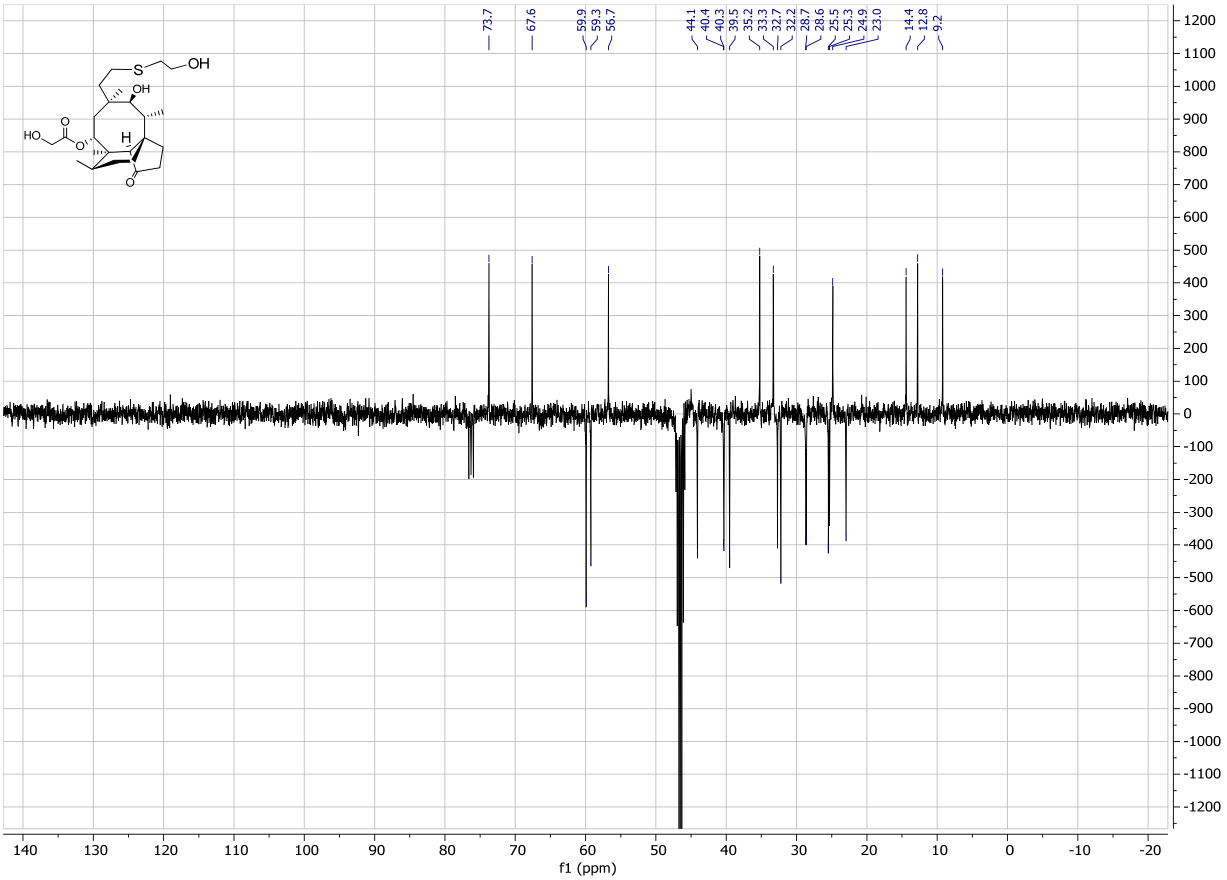


**Figure S10.** $^{1}H$ and $^{13}C$ NMR spectrum (400 MHz, MeOD) of compound **10j**.

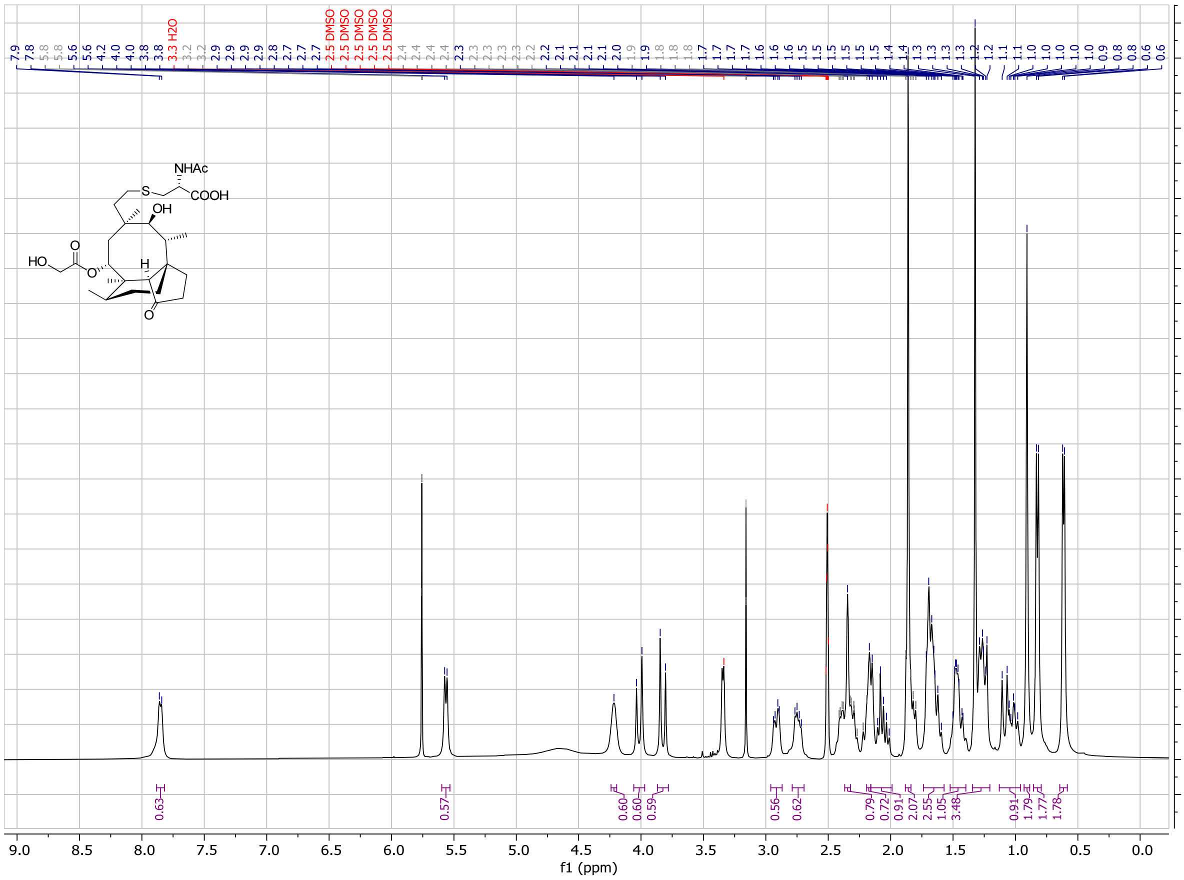


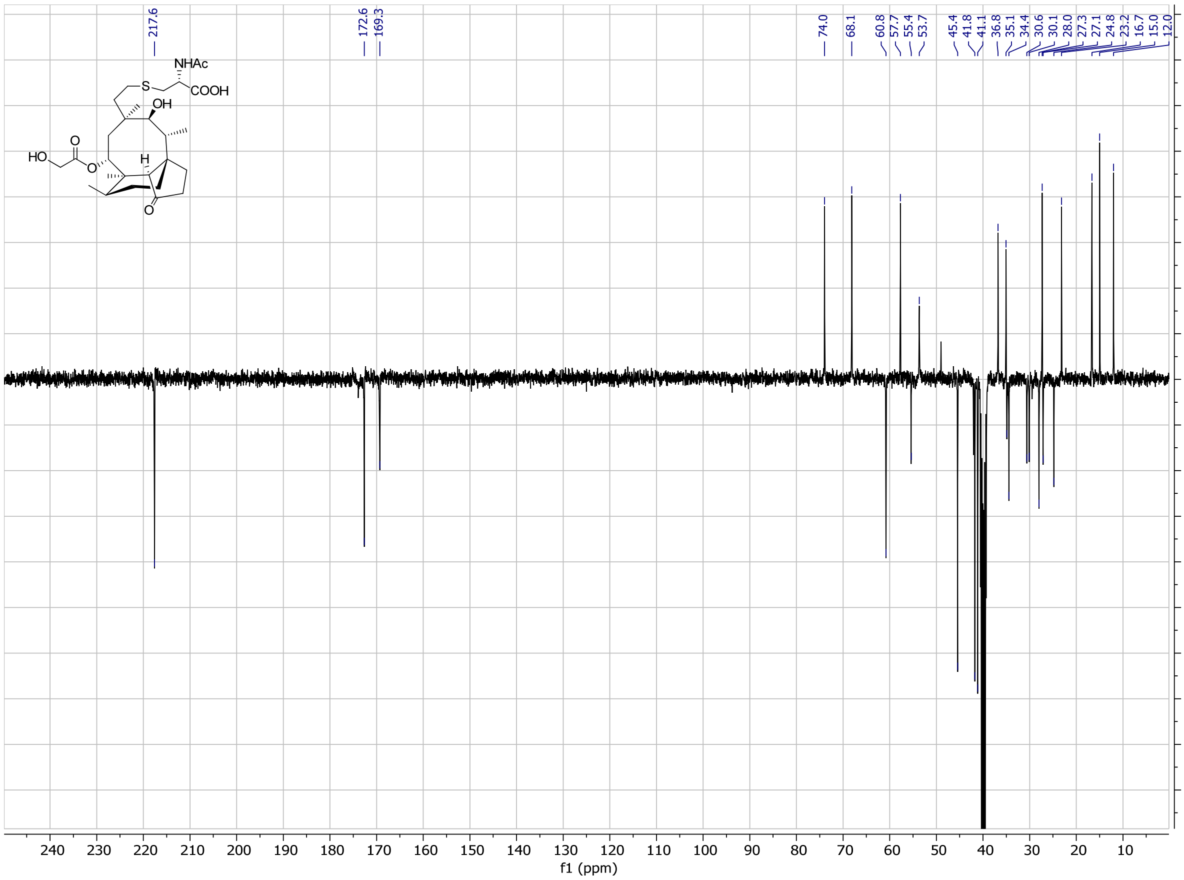


**Figure S11.** $^{1}H$ and $^{13}C$ NMR spectrum (400 MHz, DMSO) of compound **10k**.

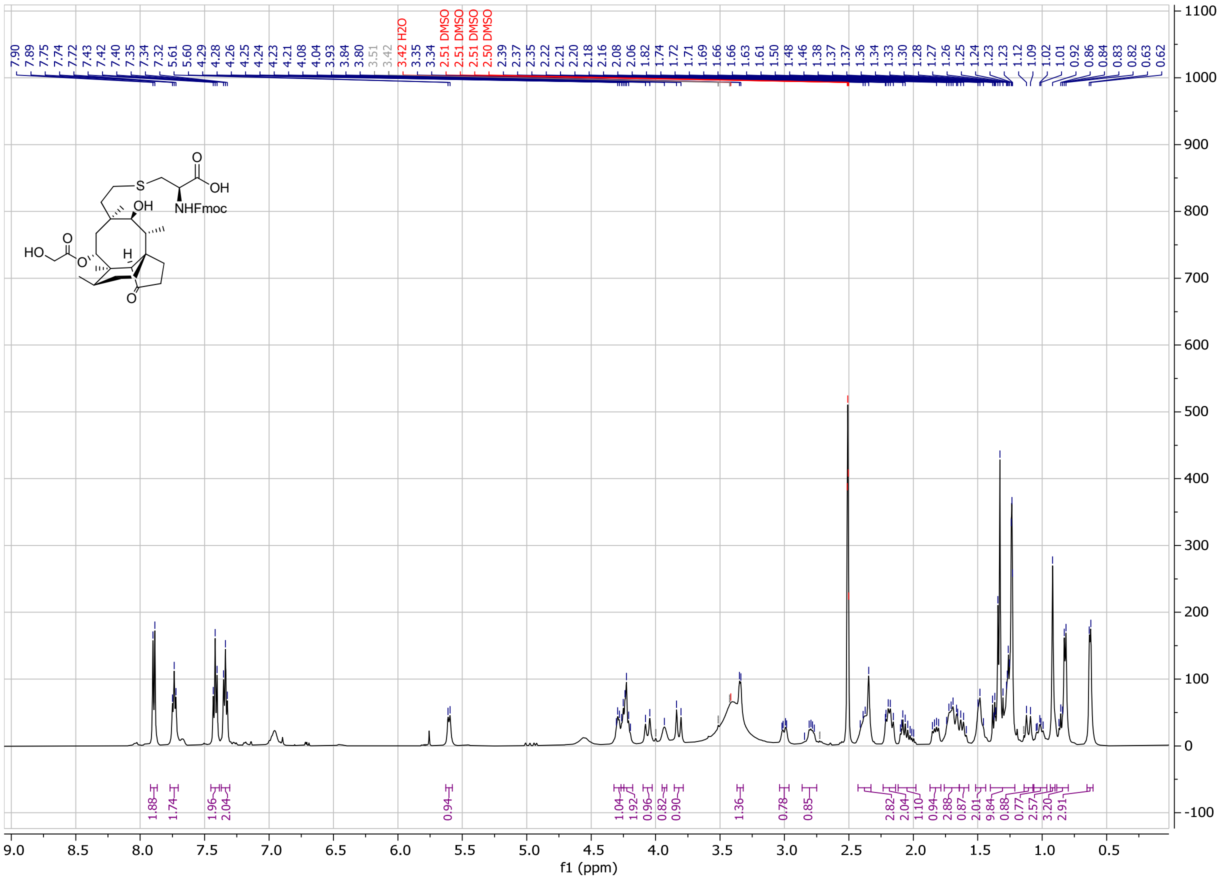

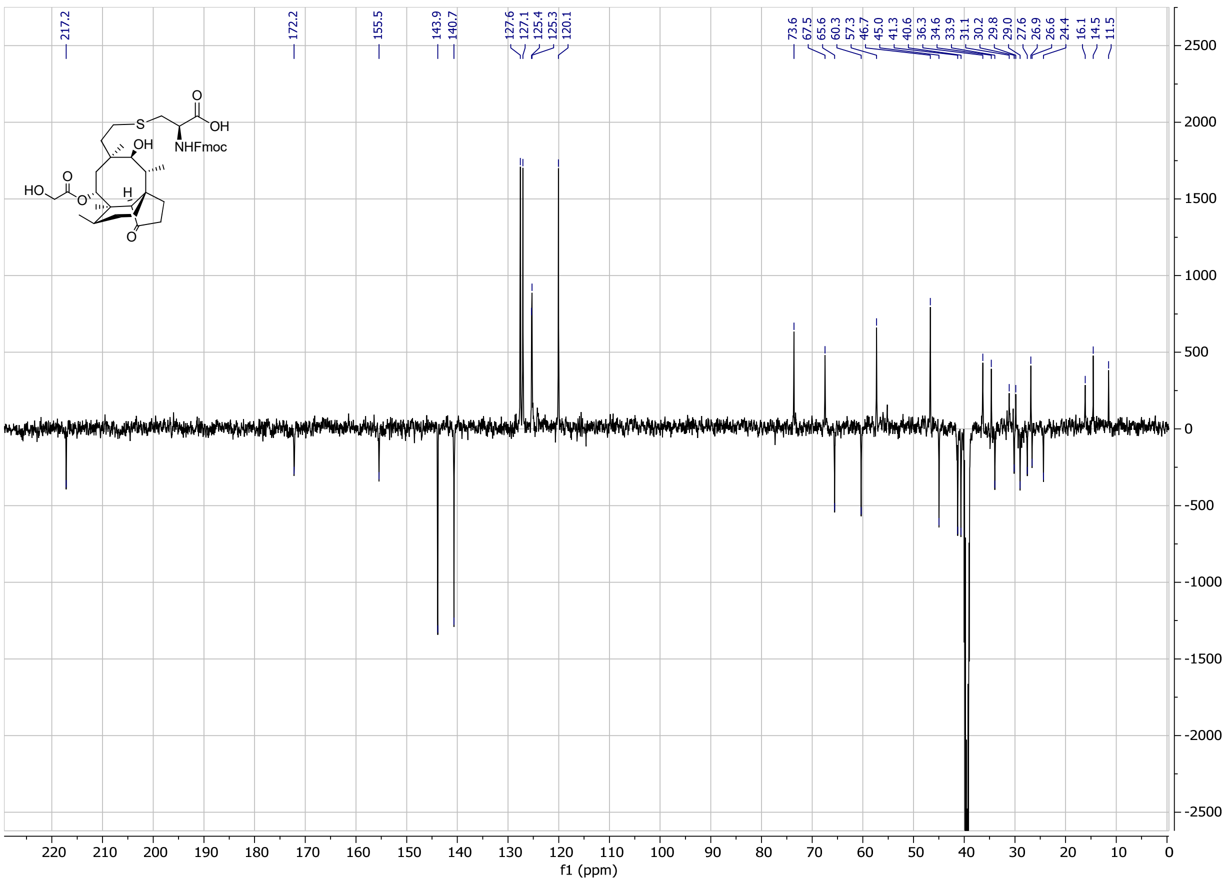

**Figure S12.** $^1H$ and $^{13}C$ NMR spectrum (500 MHz, MeOD) of compound **10l**.

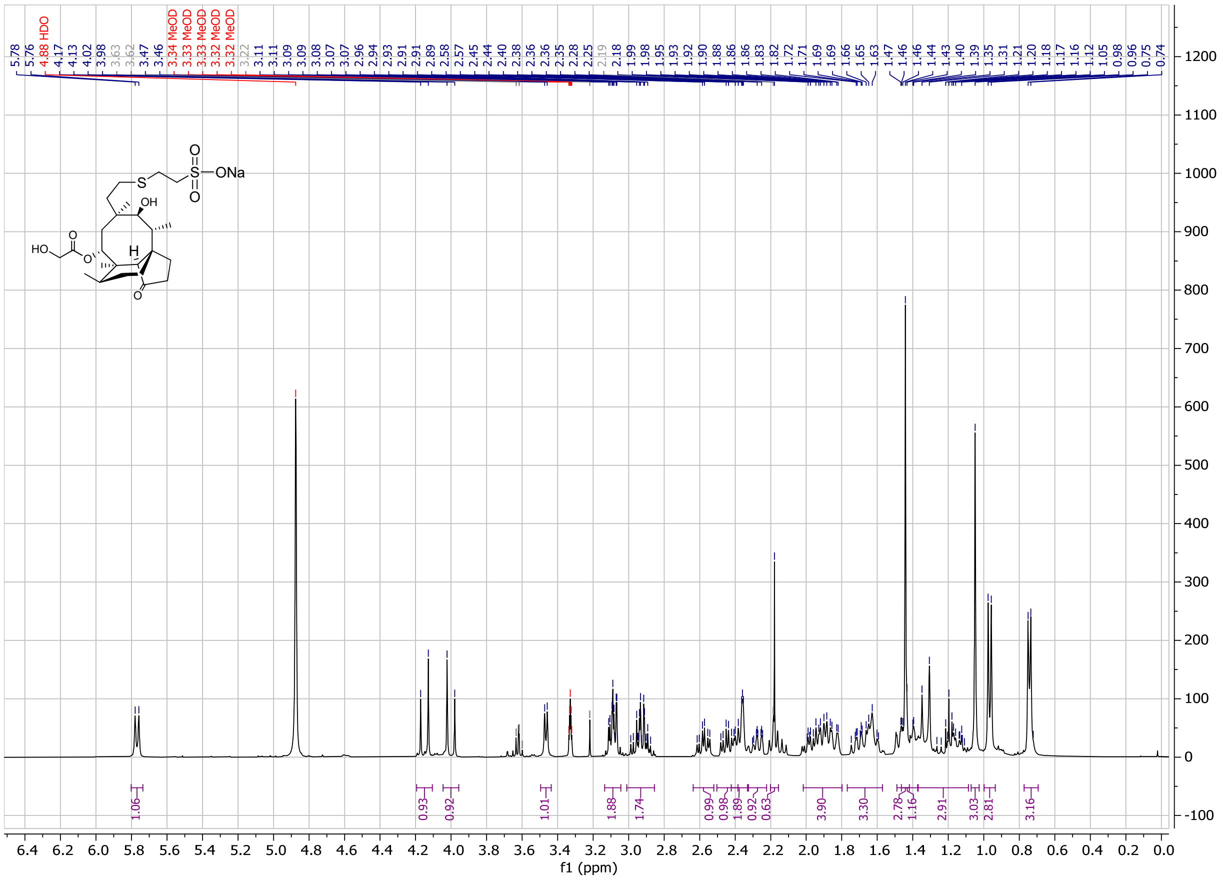

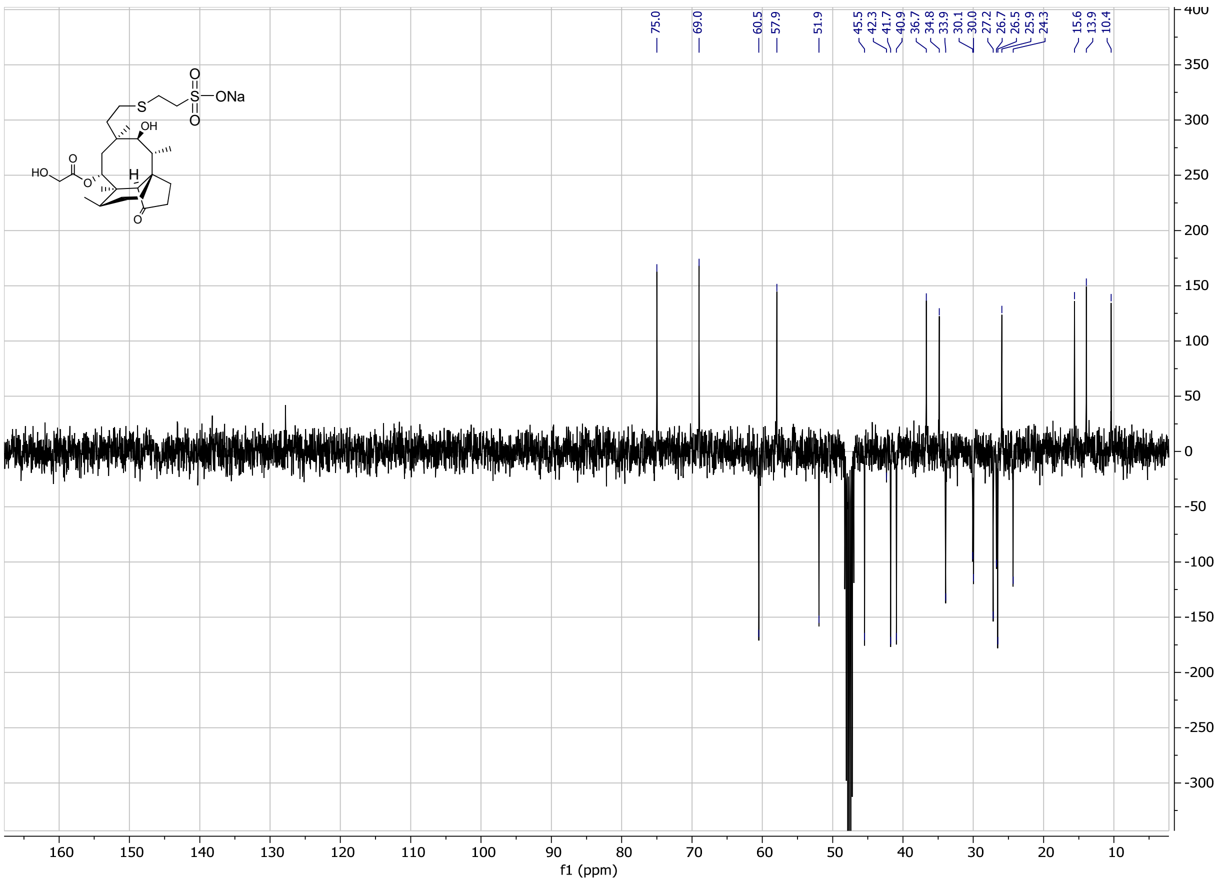

**Figure S13.** $^{1}H$ and $^{13}C$ NMR spectrum (400 MHz, MeOD) of compound **10m**.

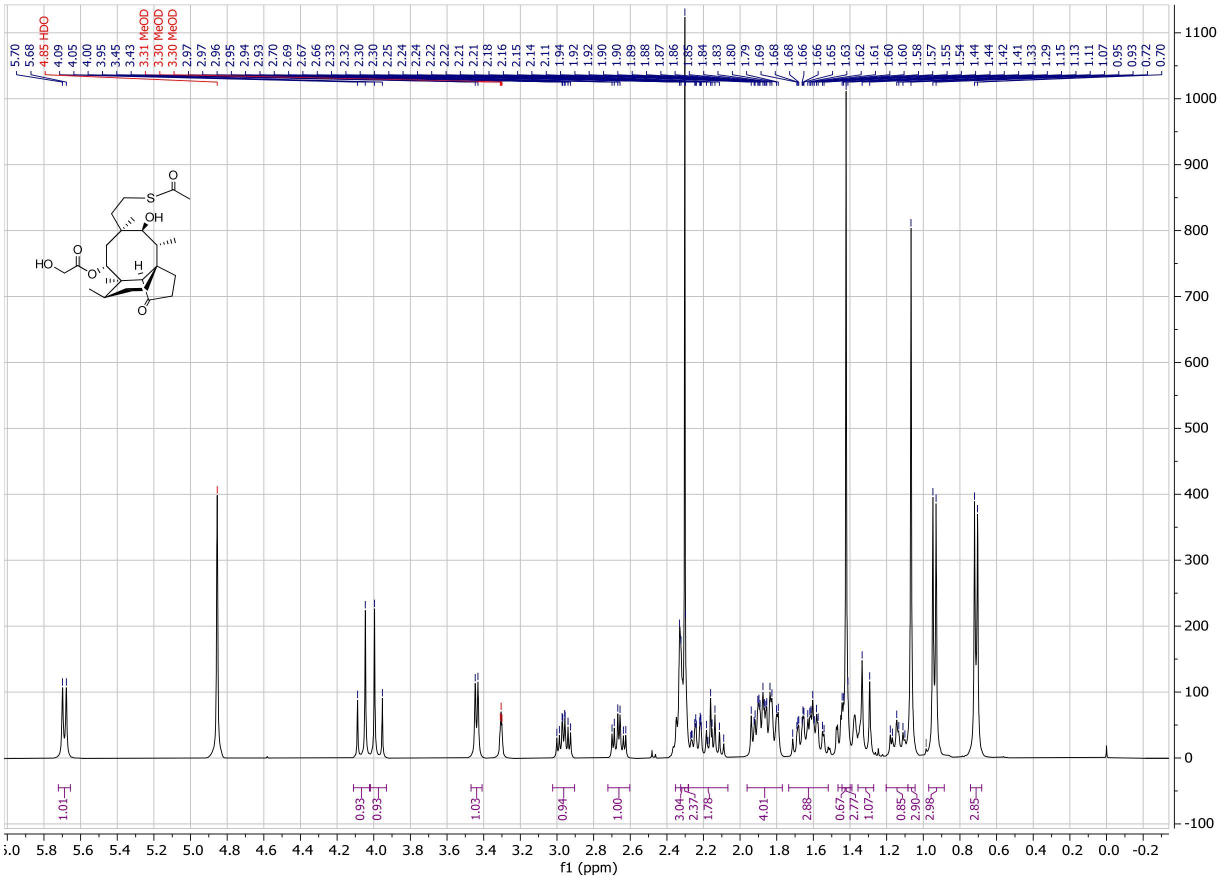



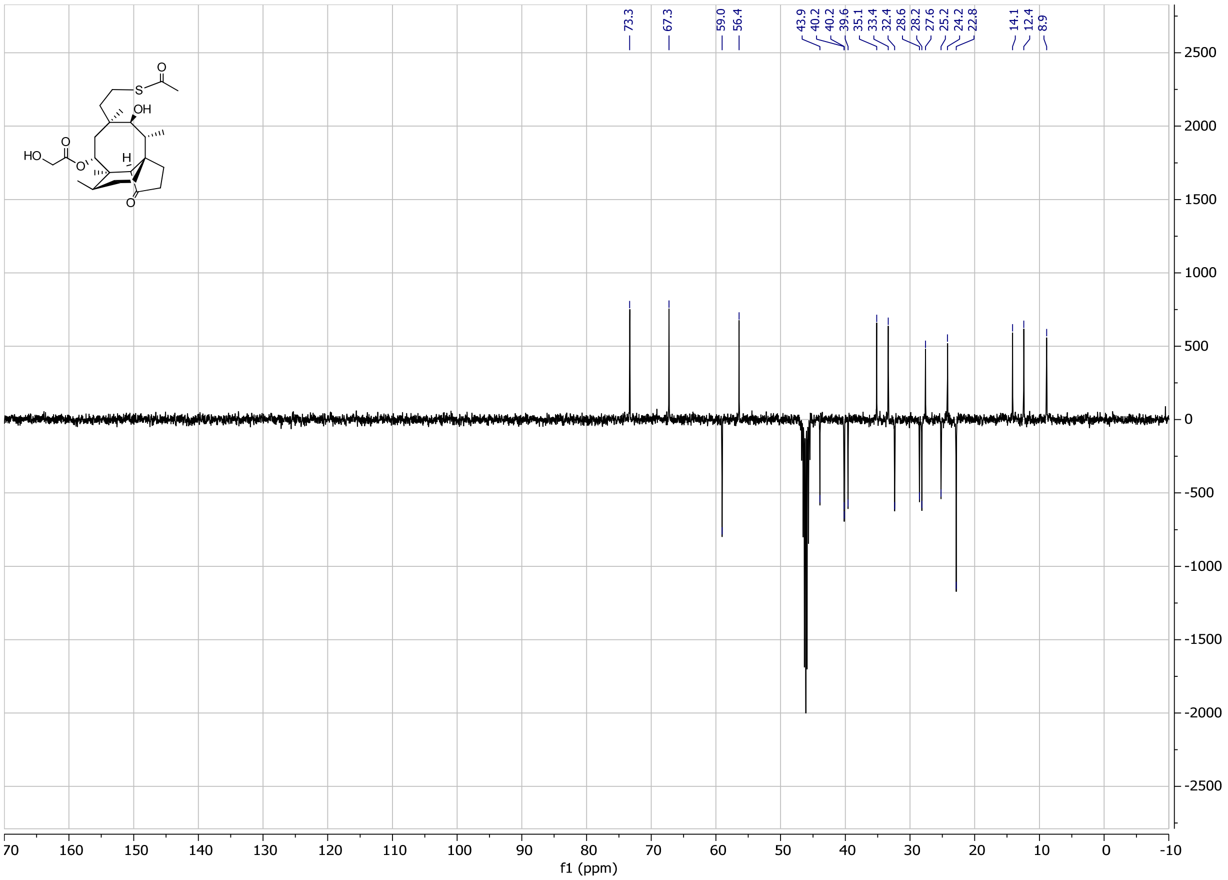



**Figure S14.** $^{1}$H and $^{13}$C NMR spectrum (400 MHz, MeOD) of compound **10n**.

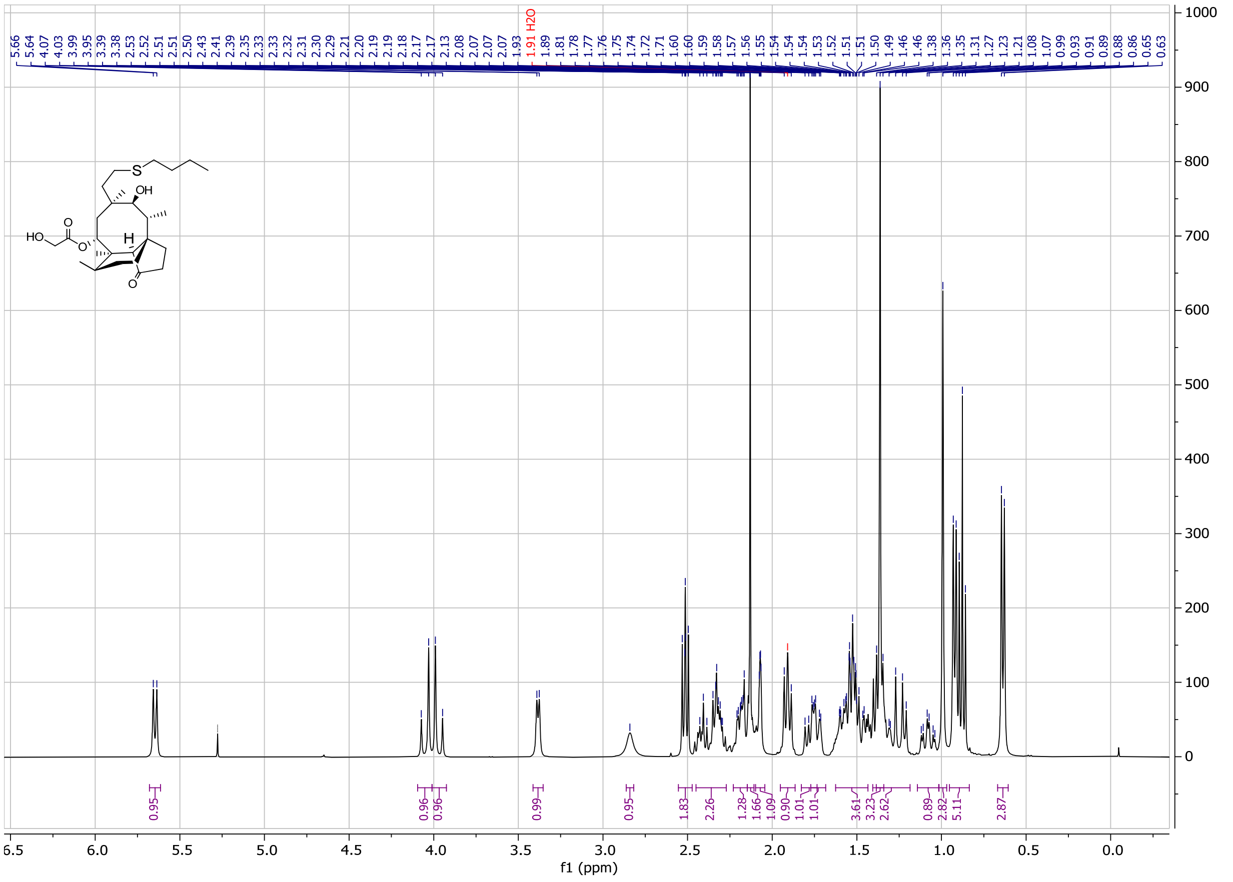

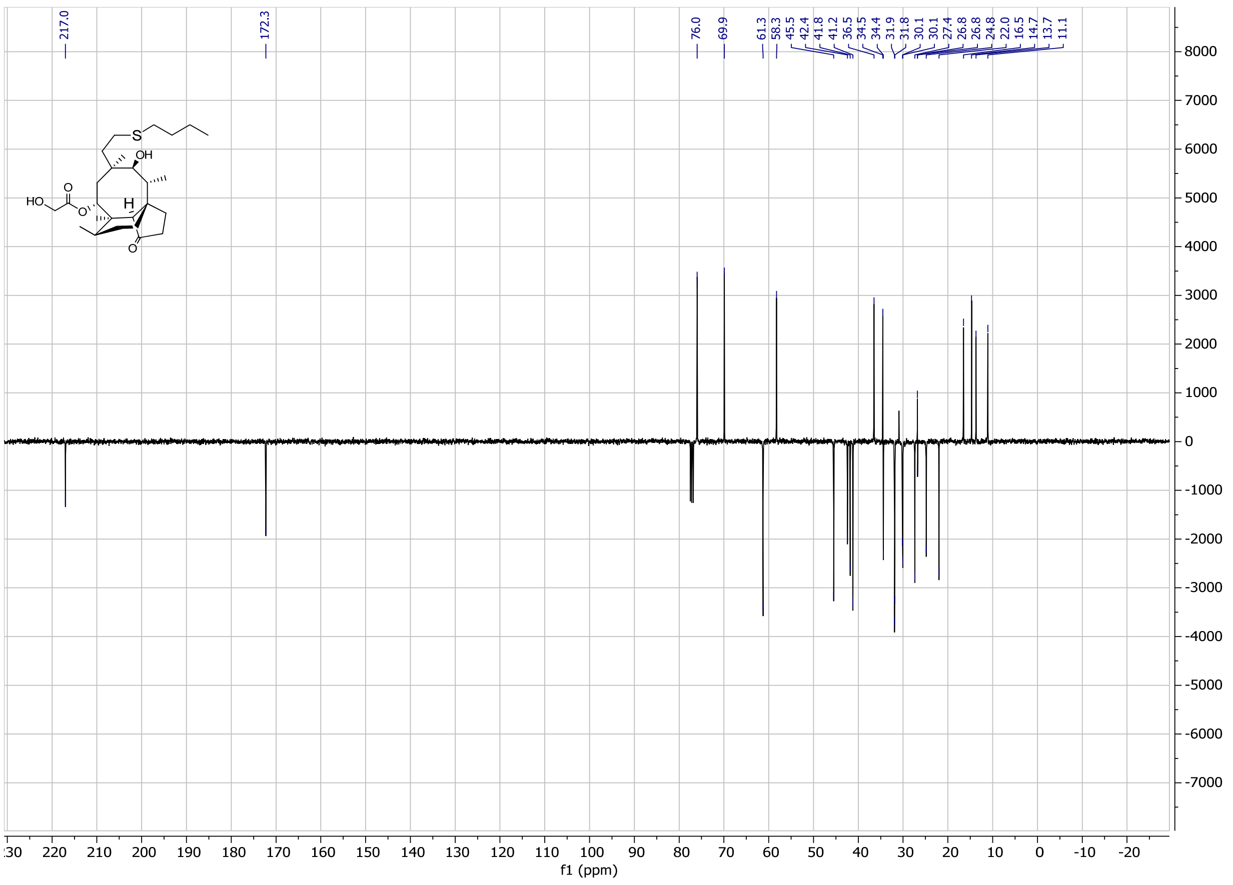

**Figure S15.** $^{1}H$ and $^{13}C$ NMR spectrum (400 MHz, $CDCl_3$) of compound **10o**.

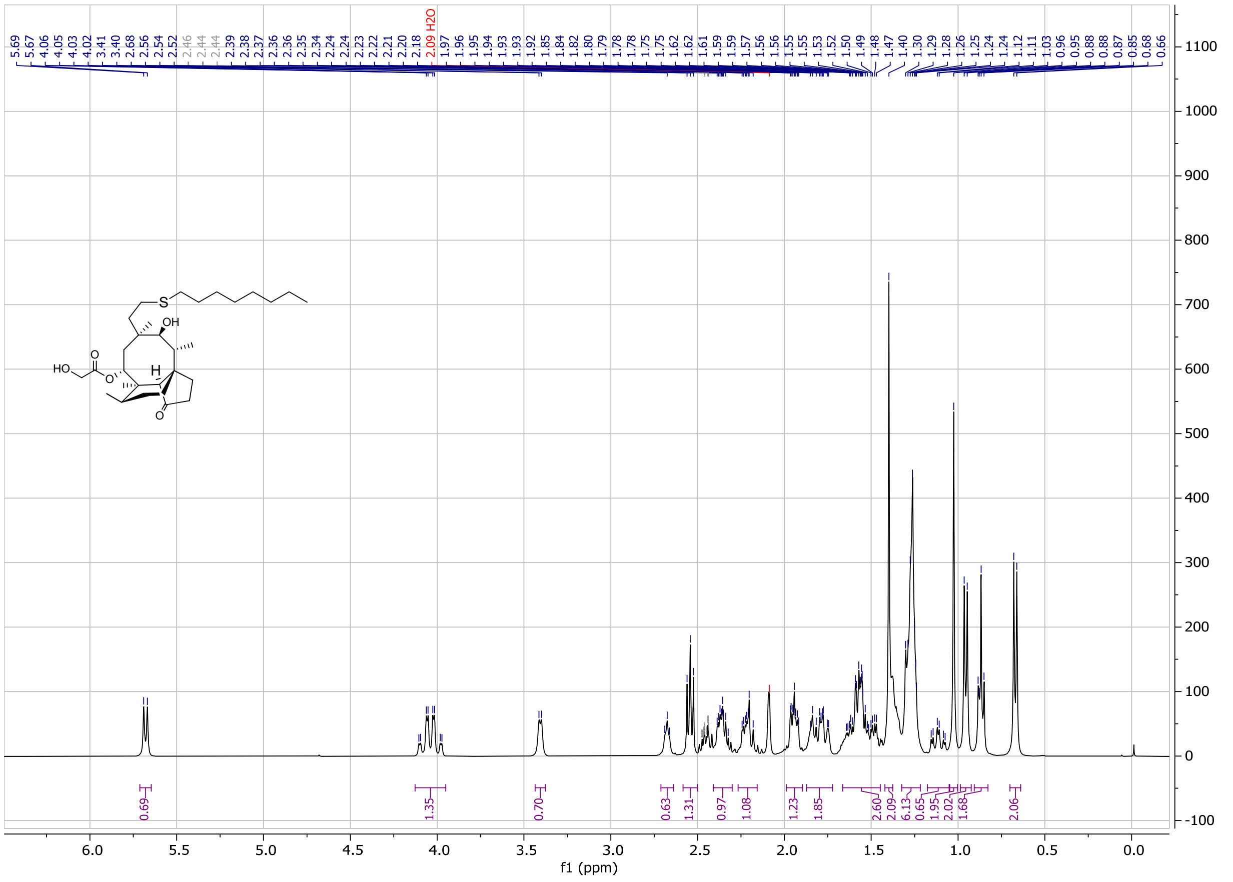

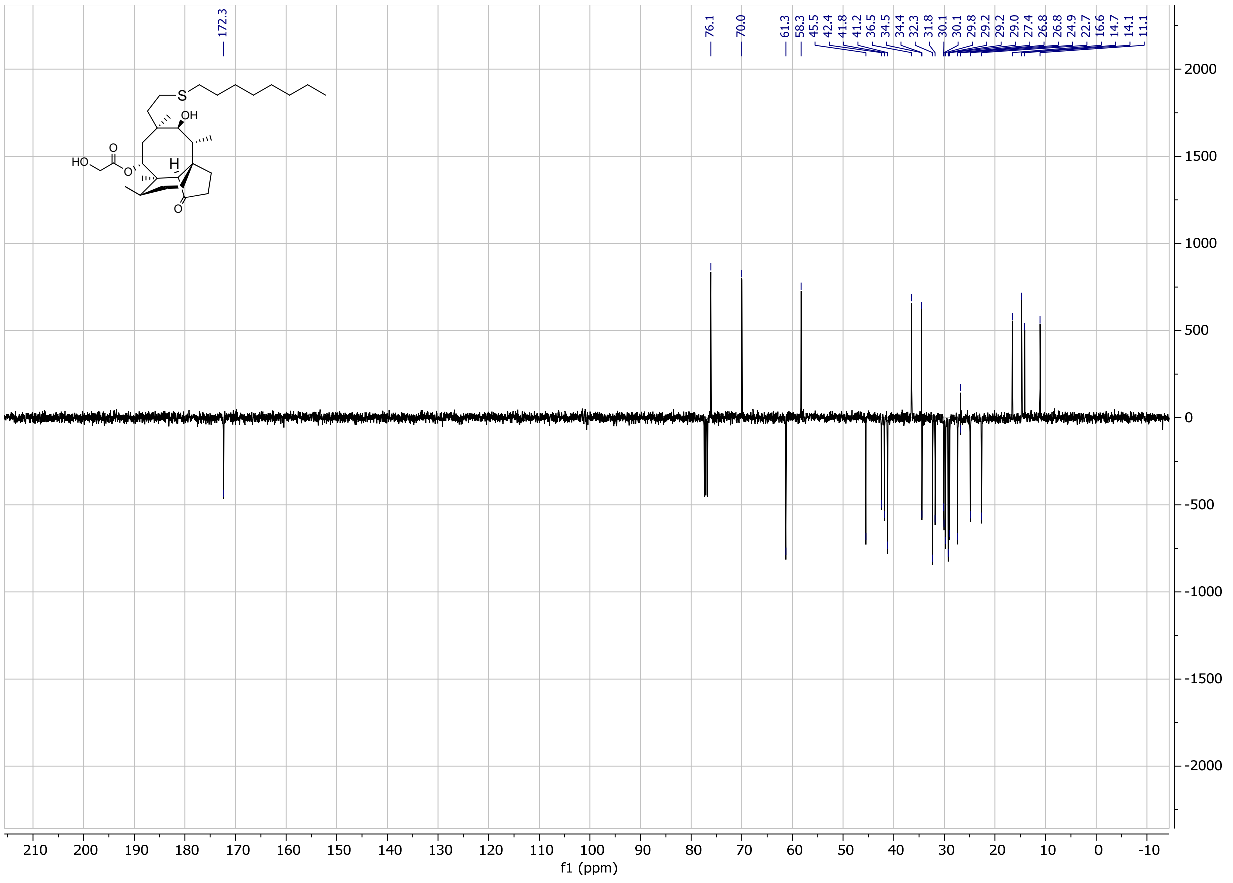

**Figure S16.** $^{1}H$ and $^{13}C$ NMR spectrum (400 MHz, $CDCl_3$) of compound **10p**.

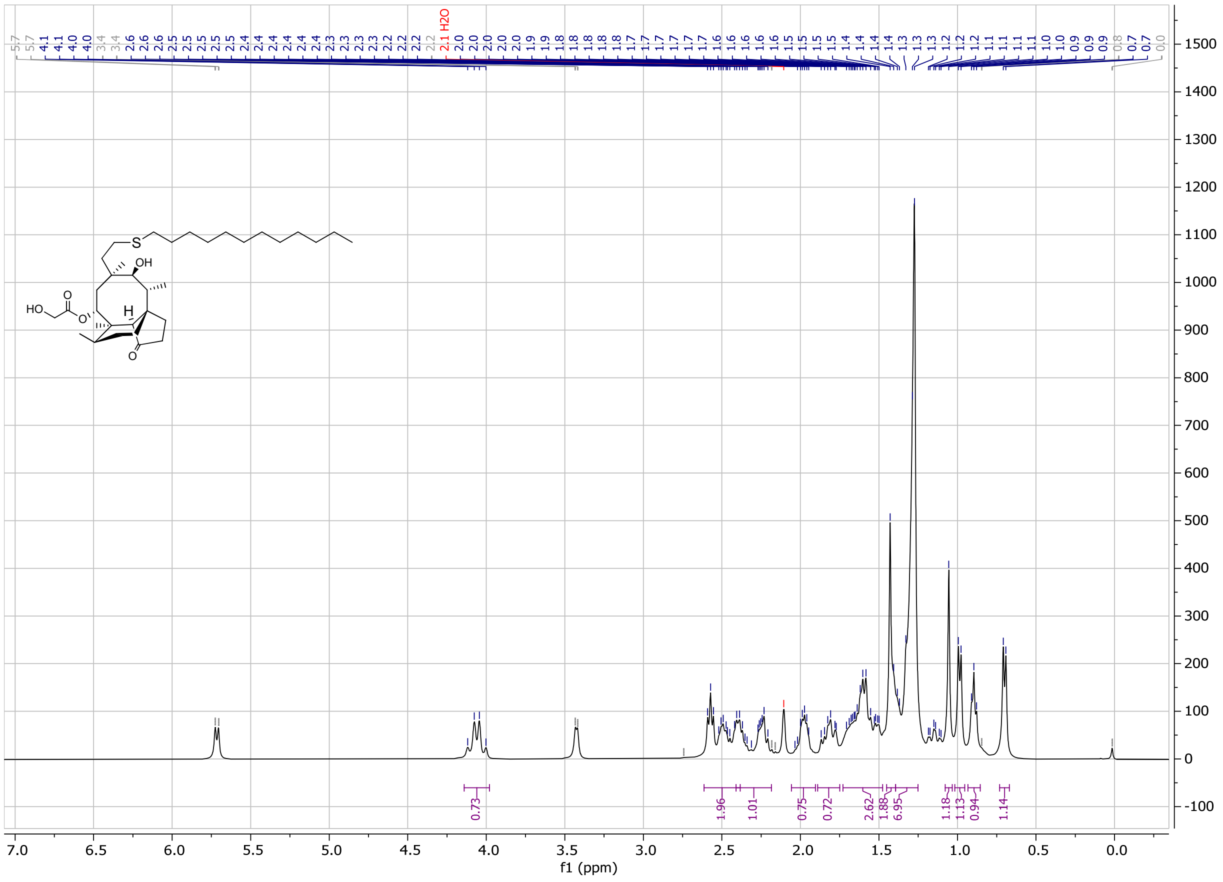

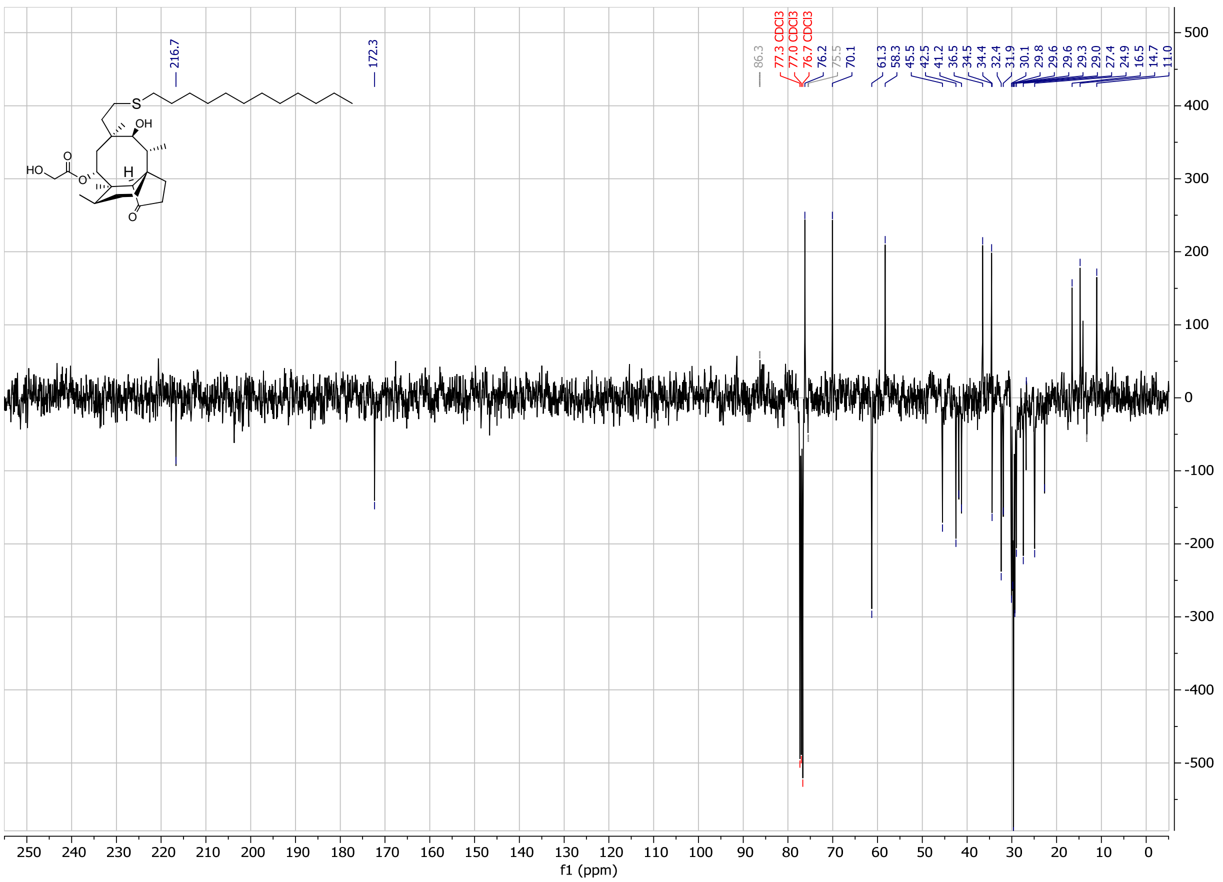

**Figure S17.** $^{1}H$ and $^{13}C$ NMR spectrum (400 MHz, $CDCl_3$) of compound **10q**.

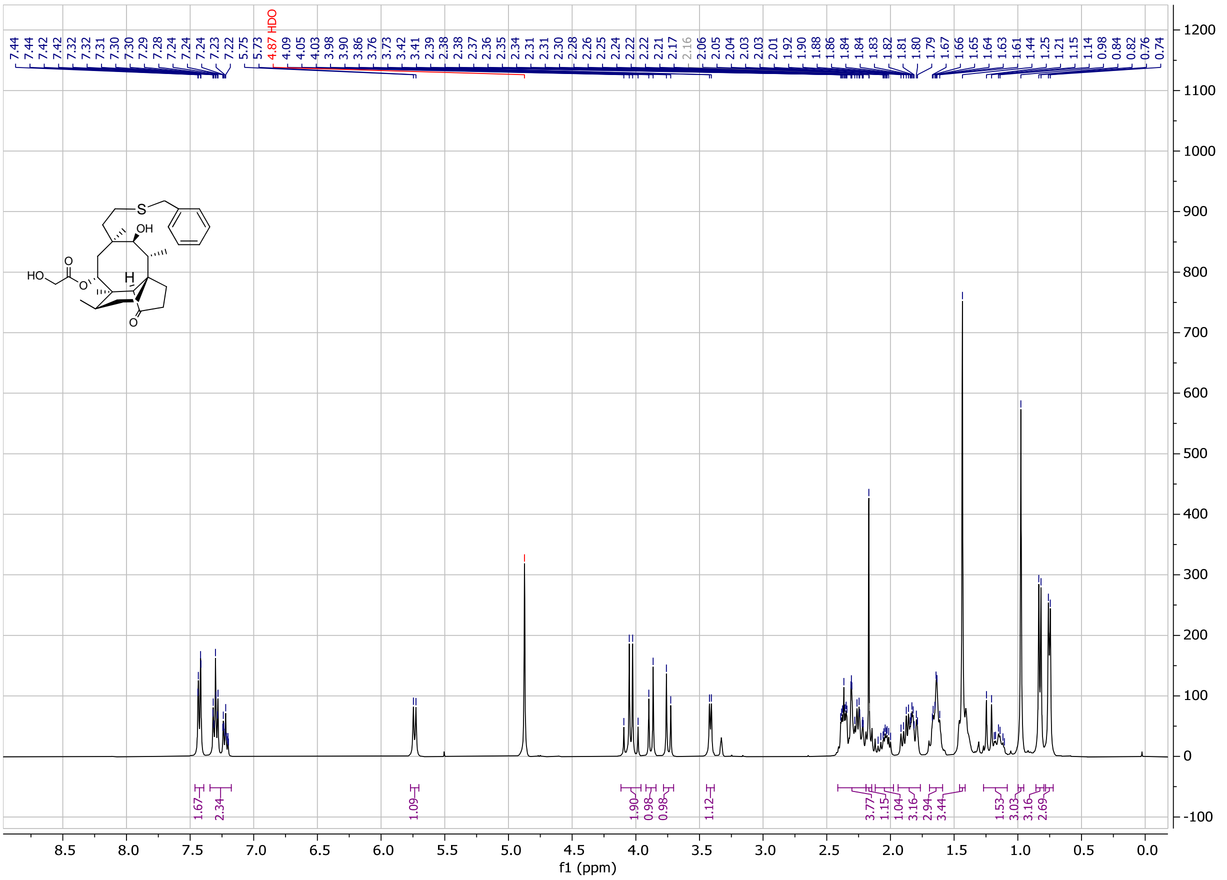


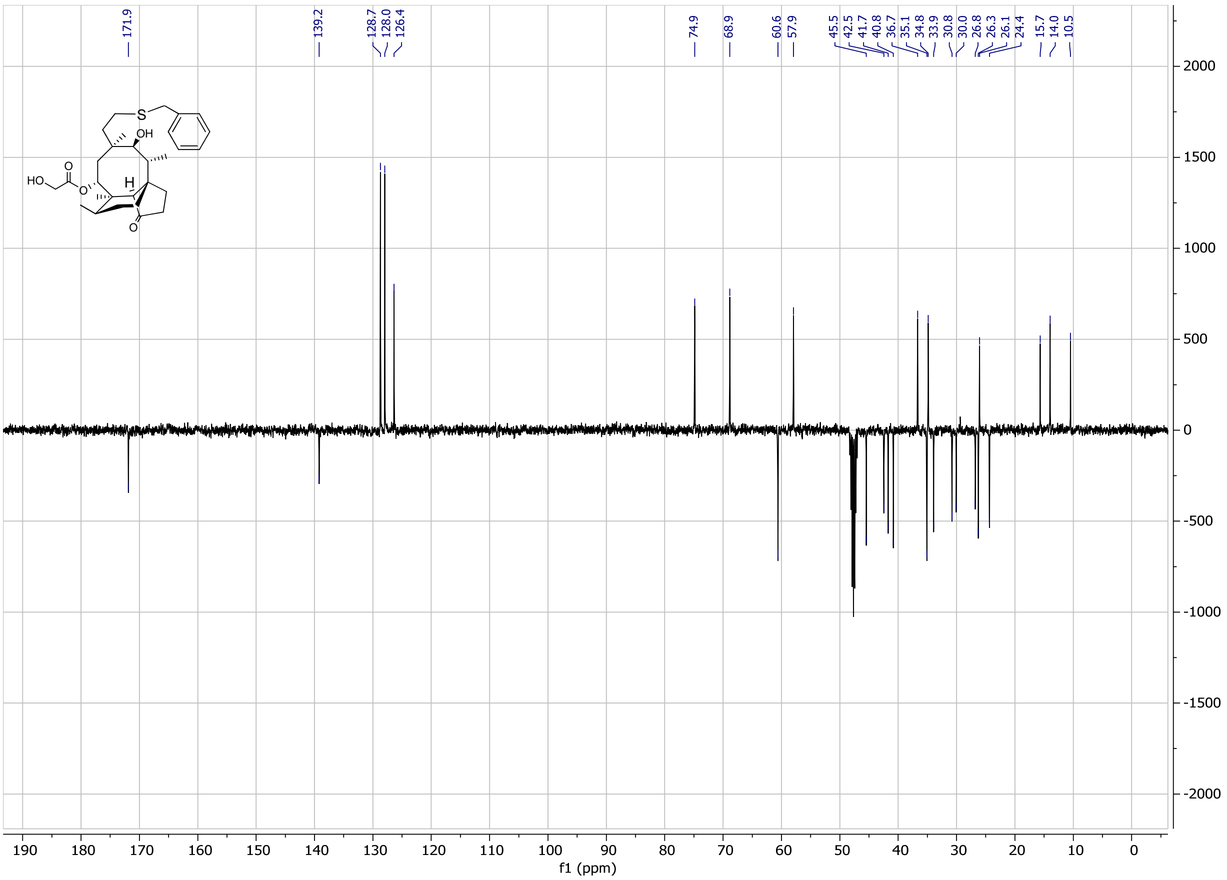


**Figure S18.** $^{1}H$ and $^{13}C$ NMR spectrum (400 MHz, MeOD) of compound **10r**.

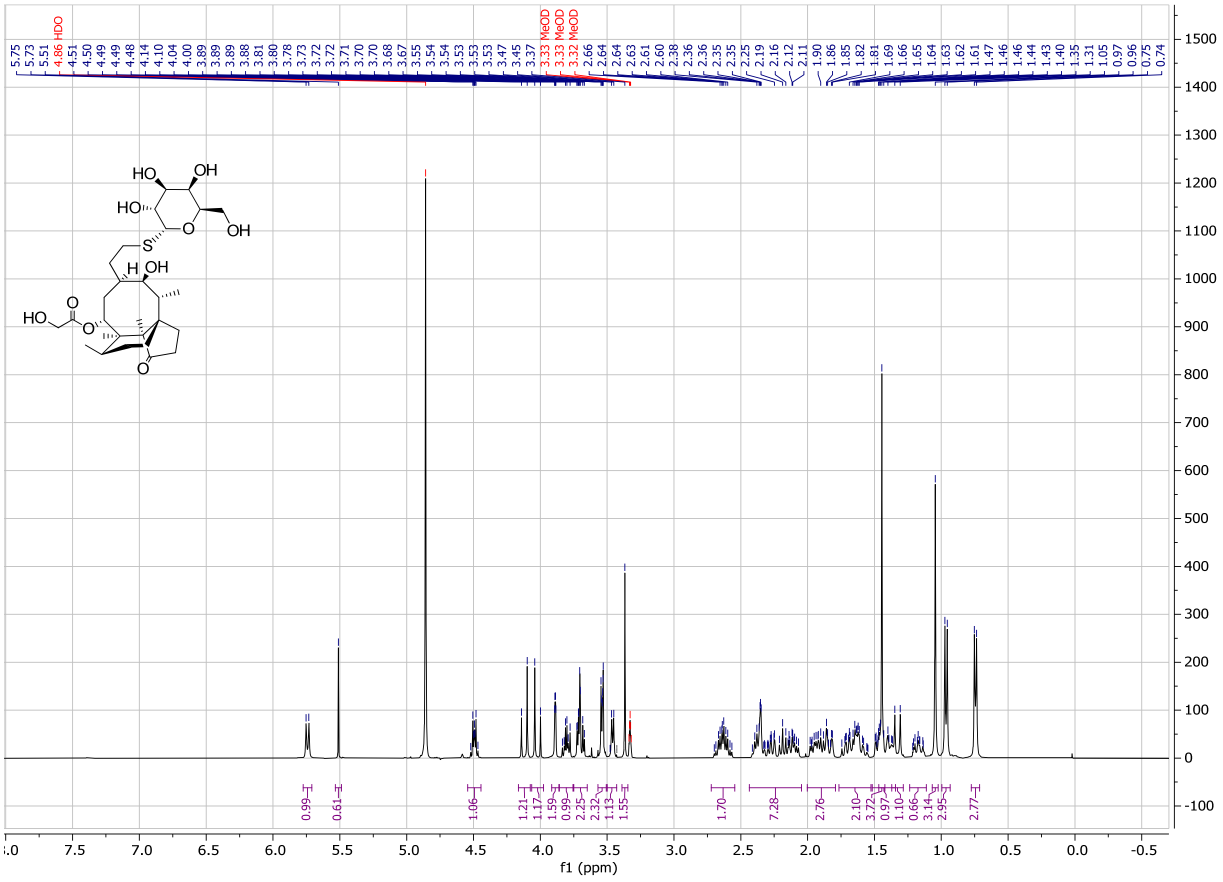

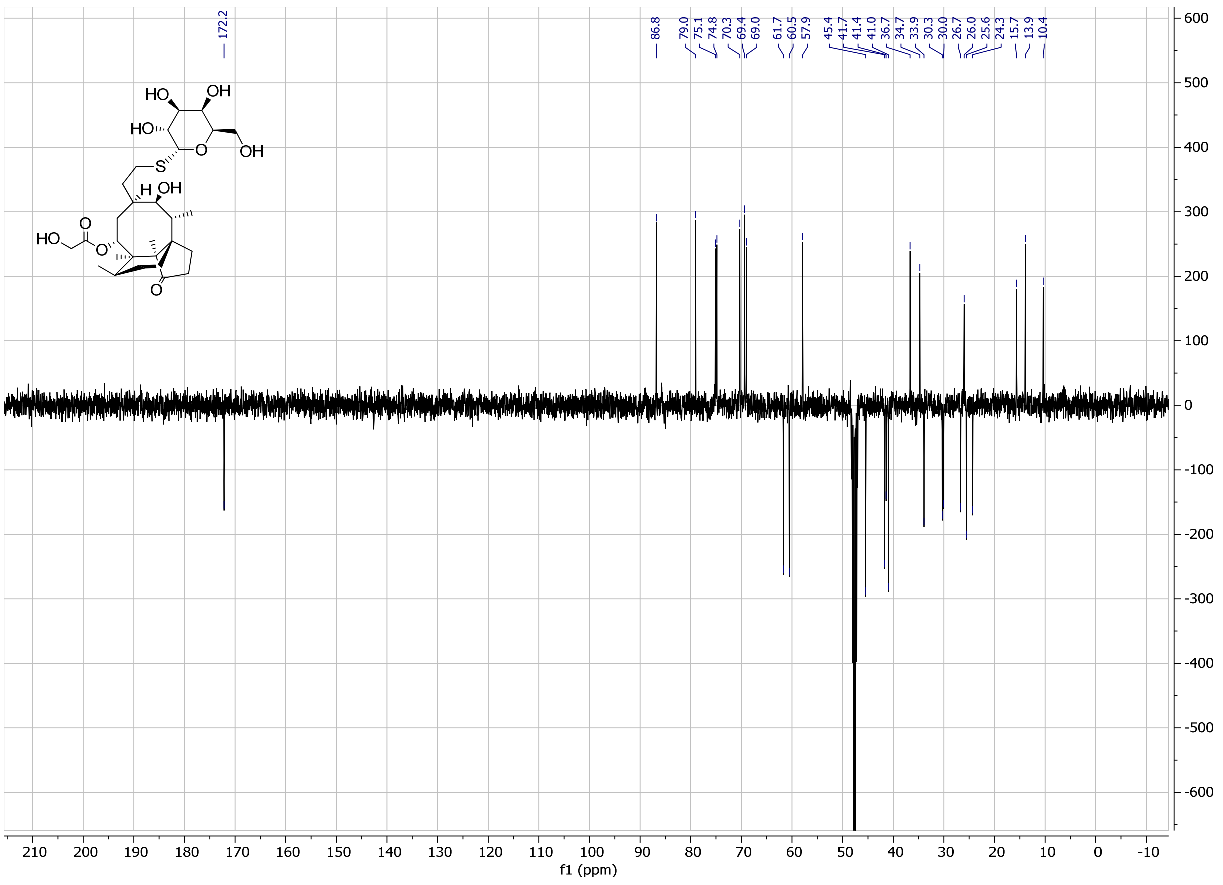

**Figure S19.** $^{1}H$ and $^{13}C$ NMR spectrum (400 MHz, MeOD) of compound **11g**.

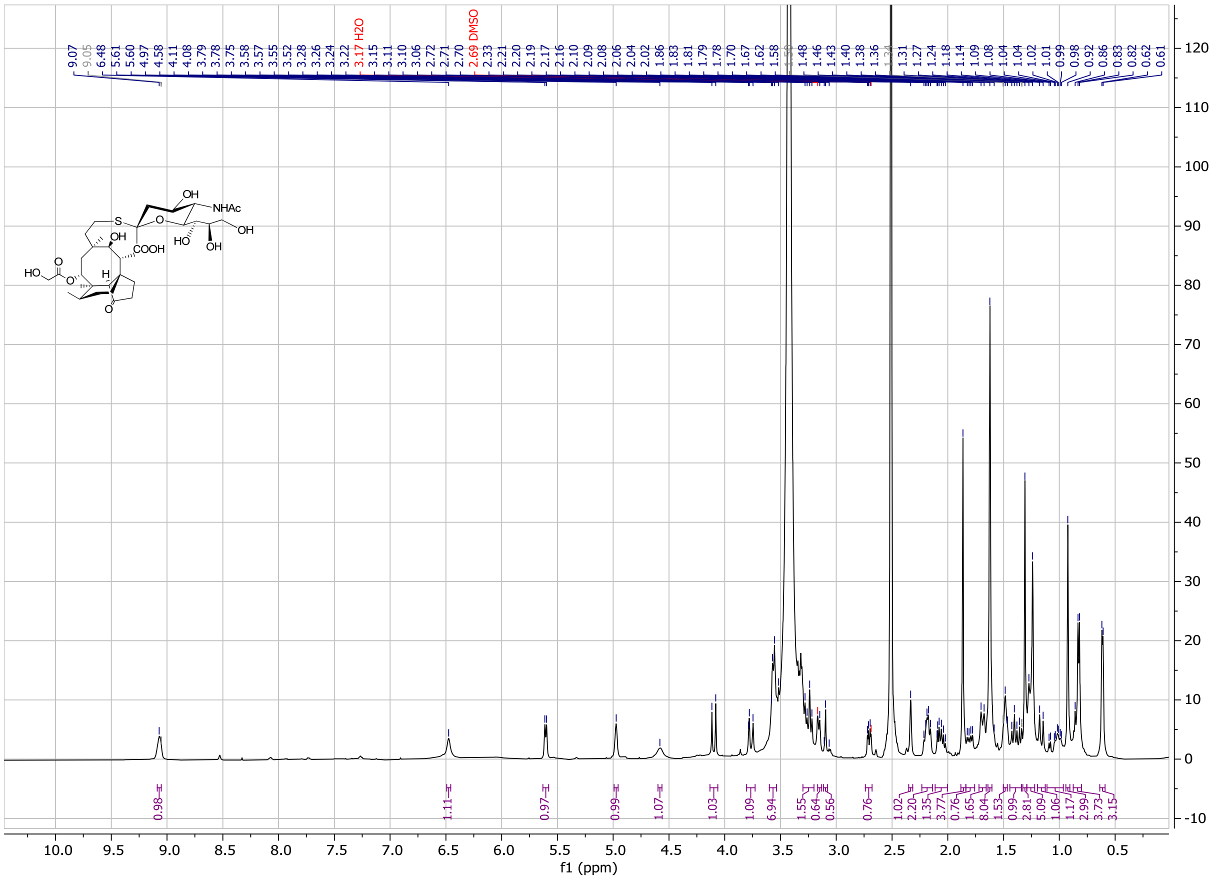

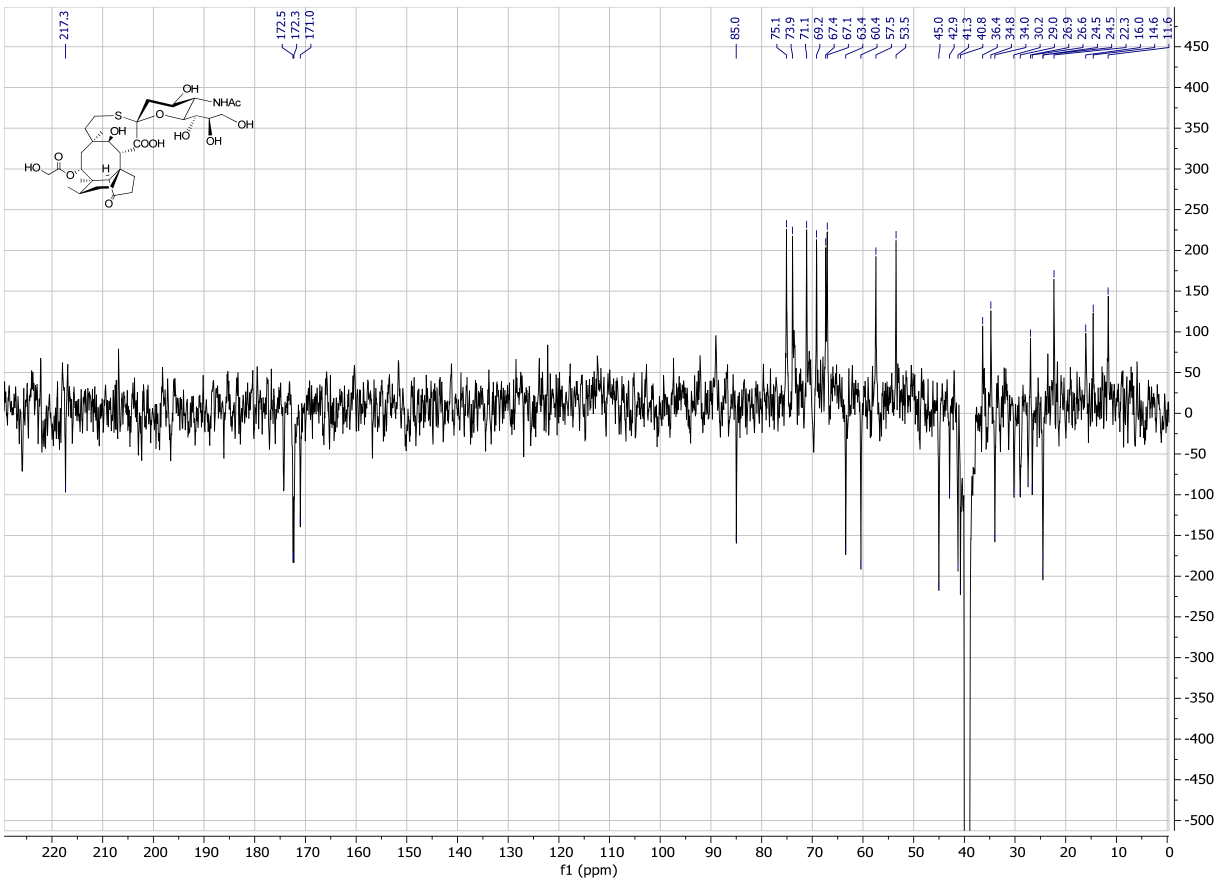

**Figure S20.** $^{1}H$ and $^{13}C$ NMR spectrum (400 MHz, DMSO) of compound **11h**.

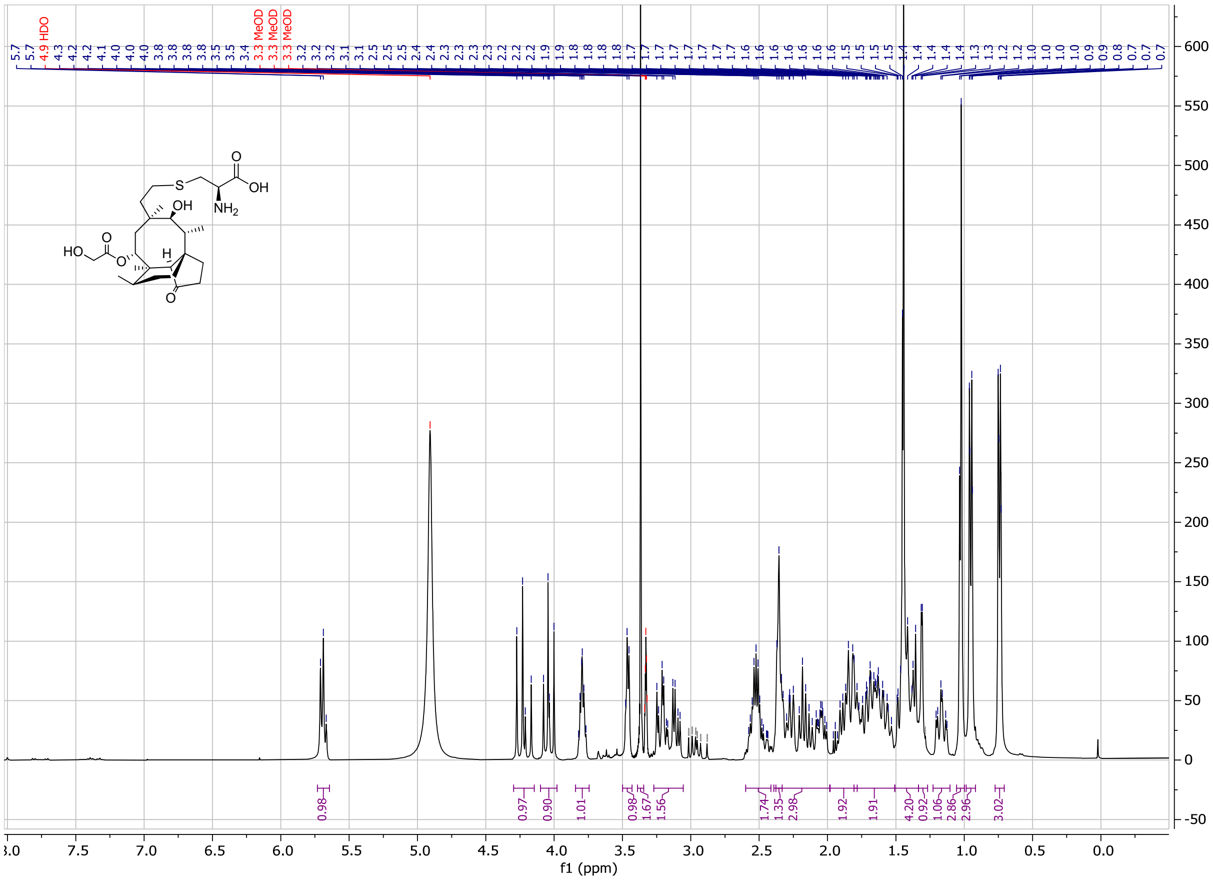


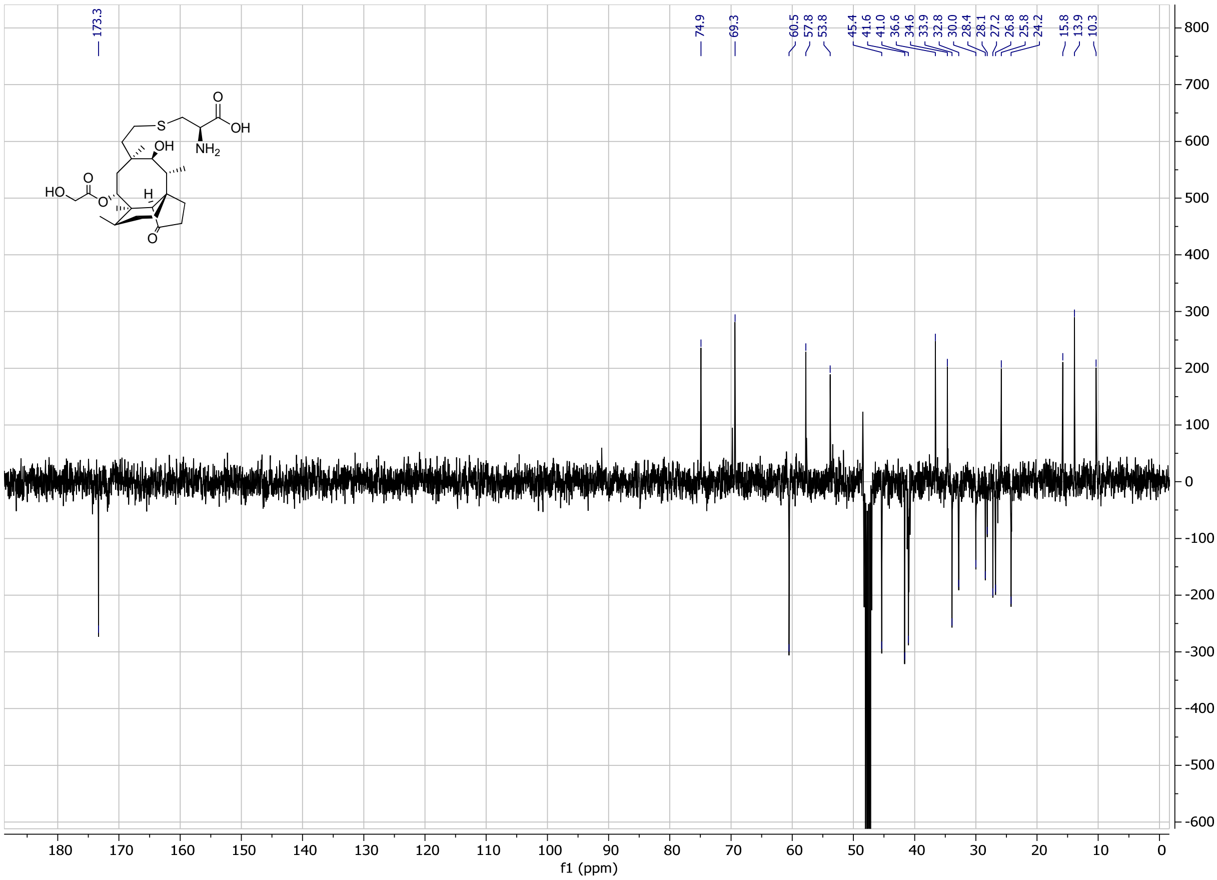


**Figure S21.** $^{1}H$ and $^{13}C$ NMR spectrum (400 MHz, MeOD) of compound **11l**.

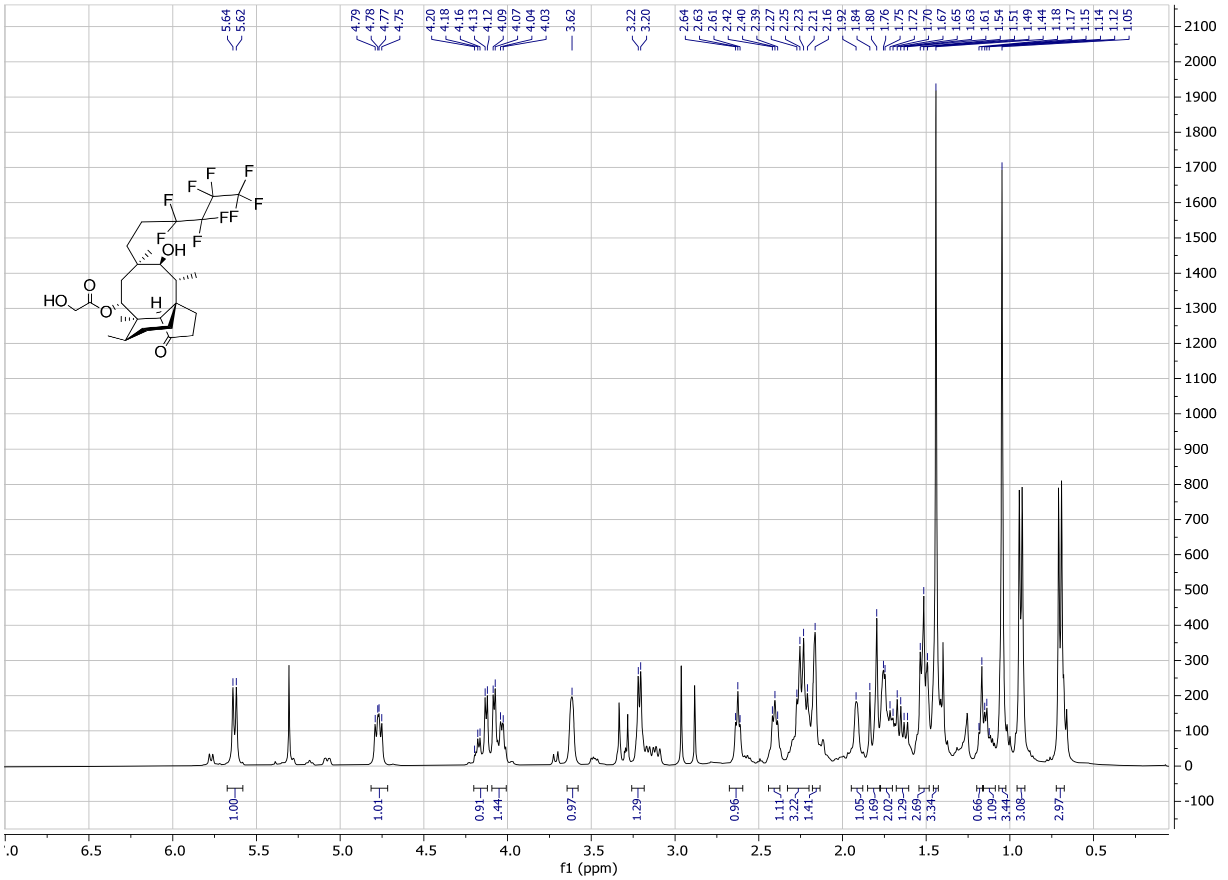

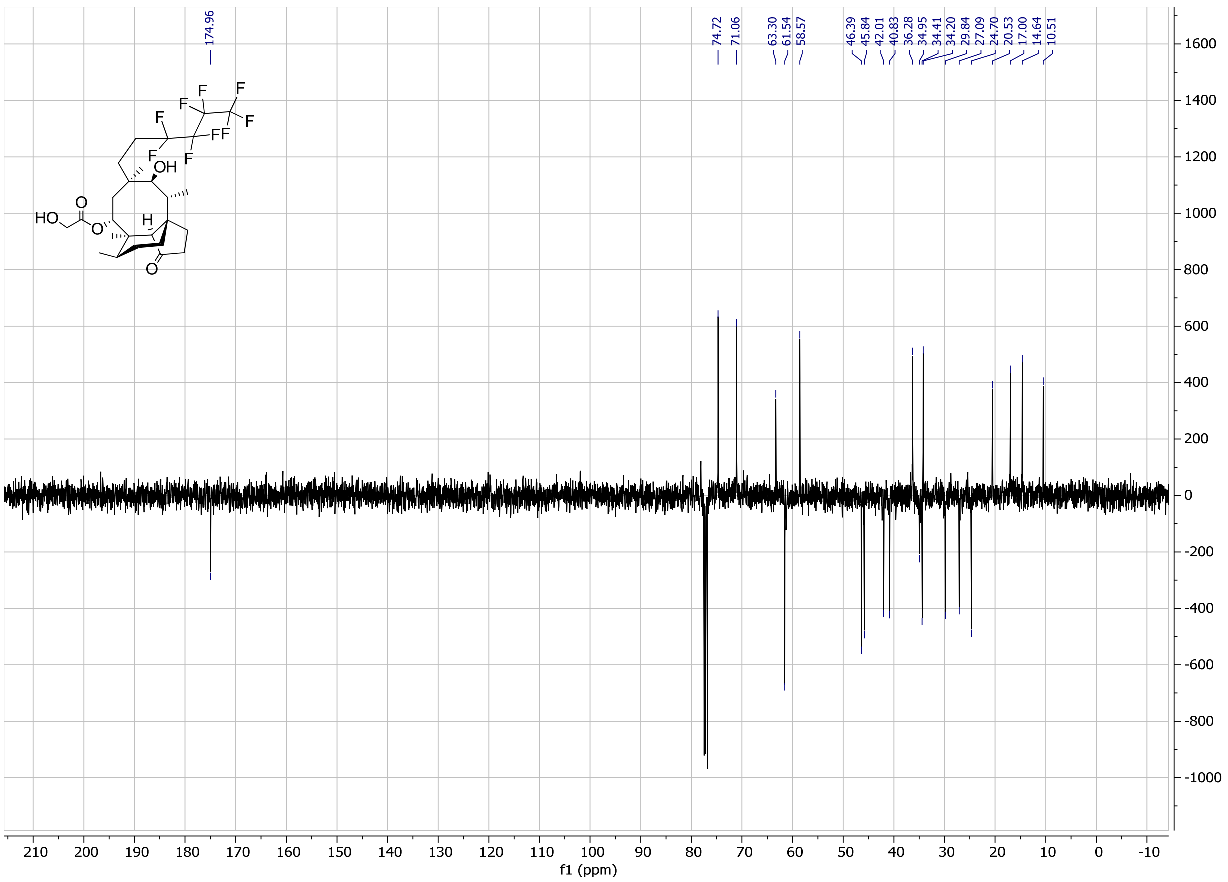

**Figure S22.** $^1$H and $^{13}$C NMR spectrum (400 MHz, $CDCl_3$) of compound **13a**.

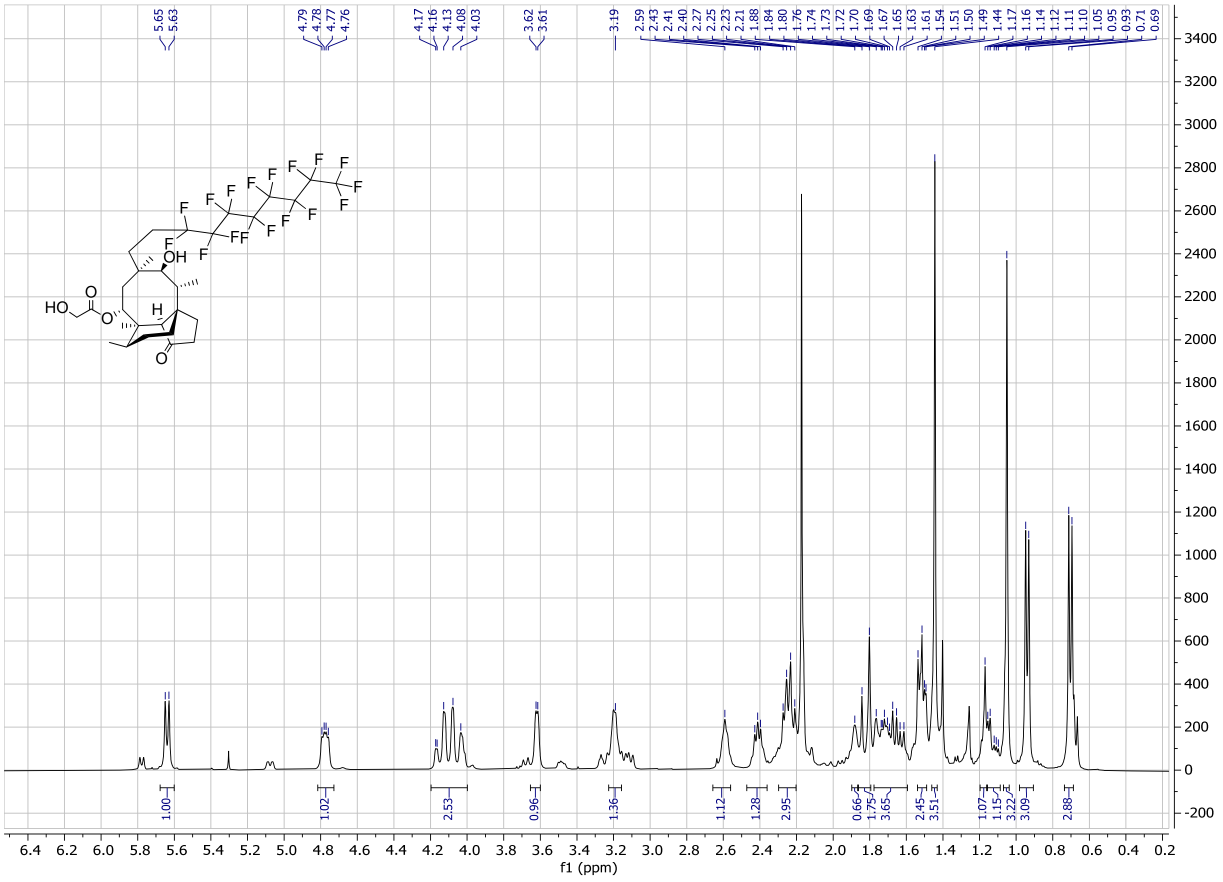

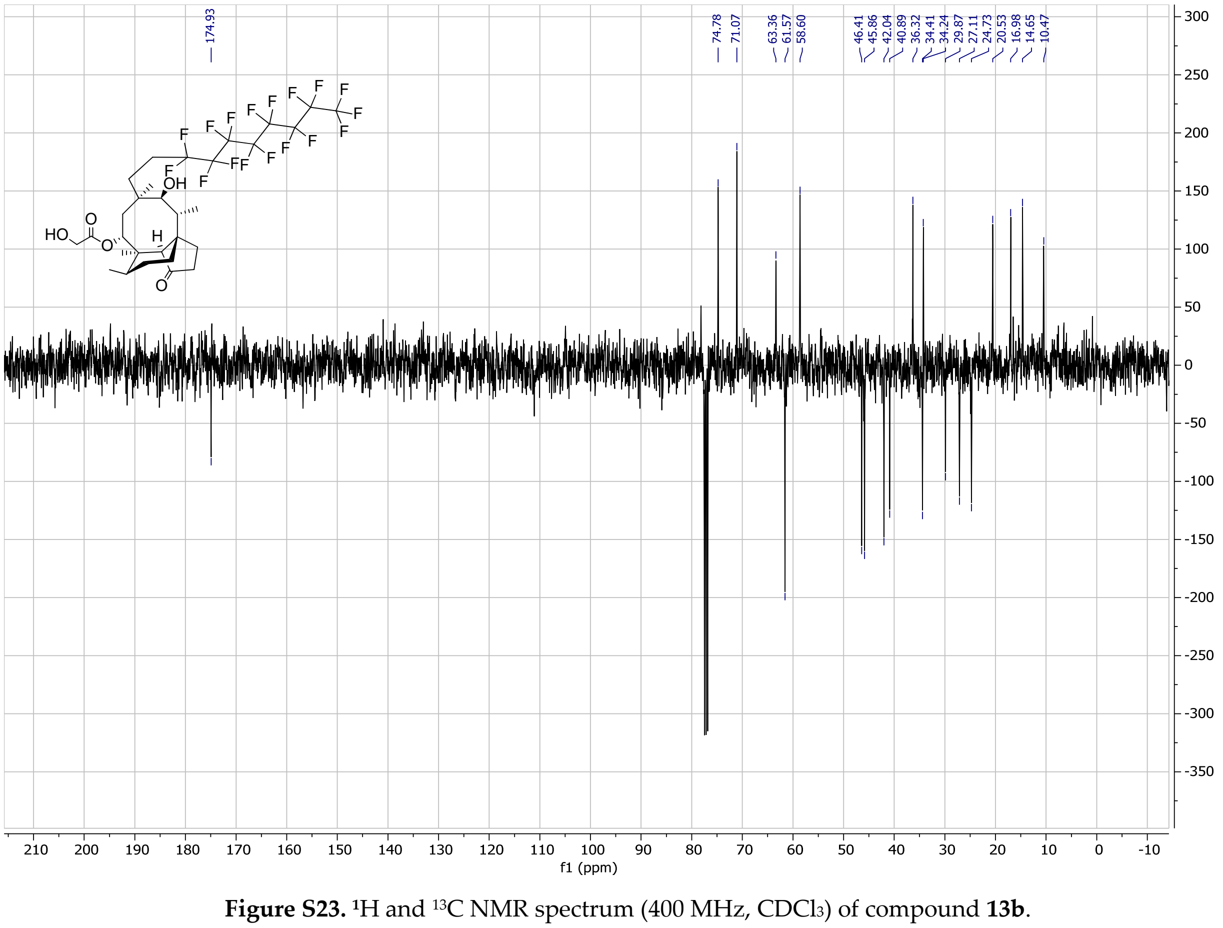

**Figure S23.** $^{1}H$ and $^{13}C$ NMR spectrum (400 MHz, $CDCl_3$) of compound **13b**.

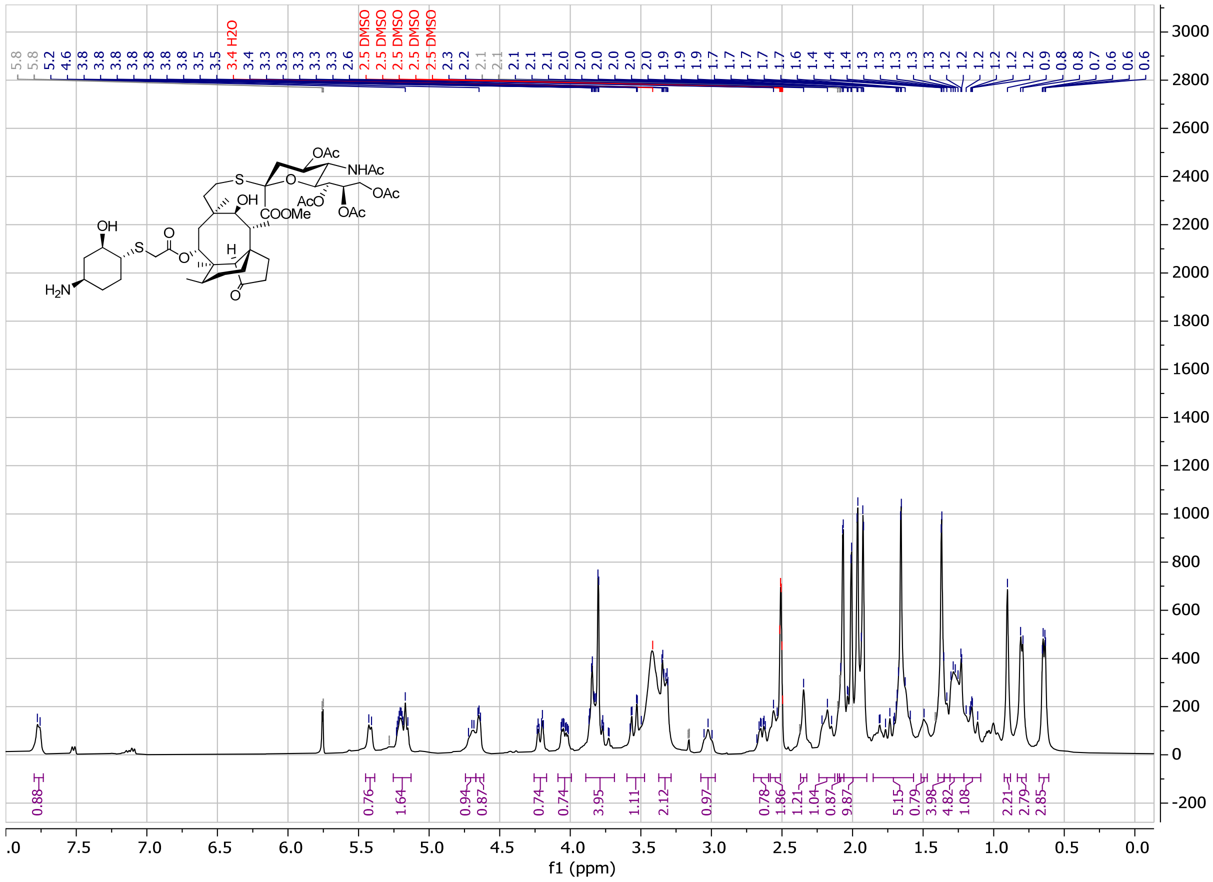

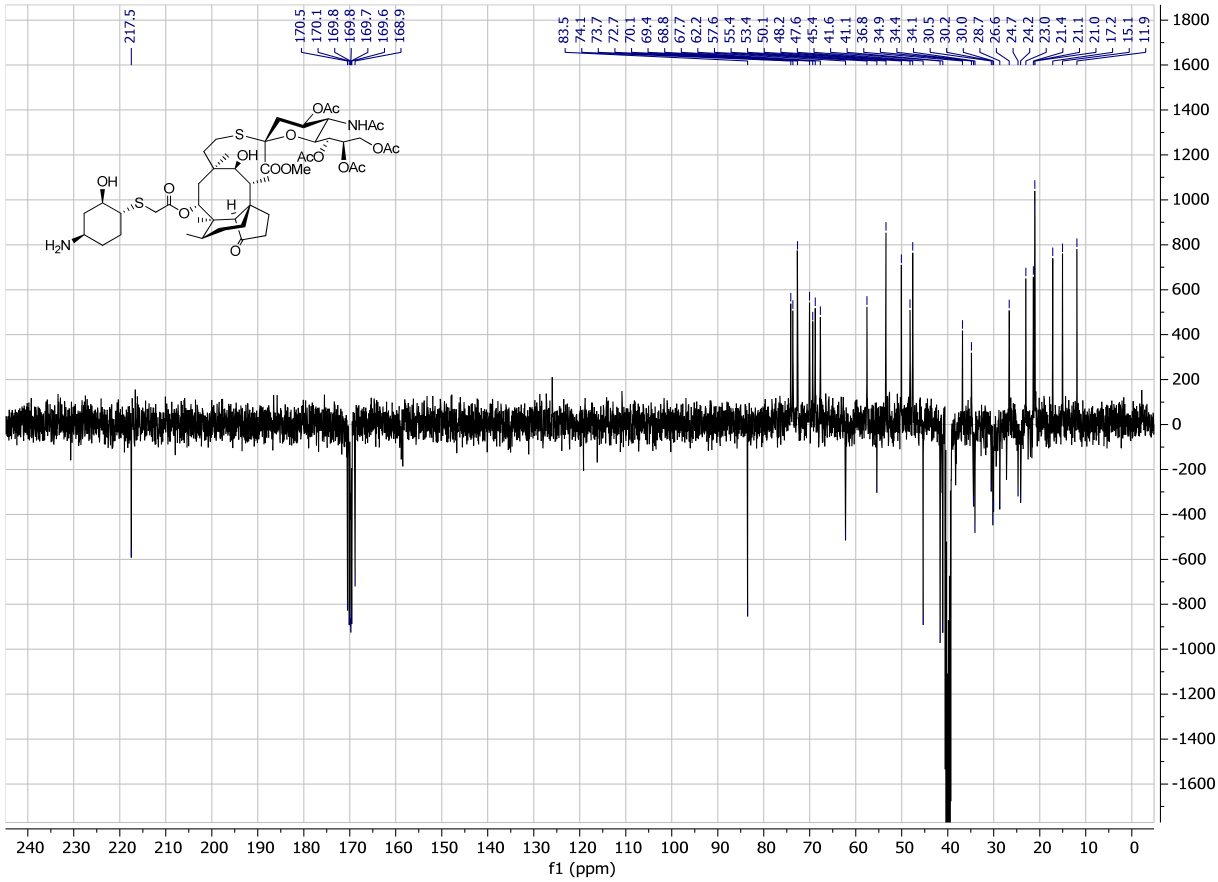

**Figure S24.** $^{1}H$ and $^{13}C$ NMR spectrum (400 MHz, DMSO) of compound **14h**.

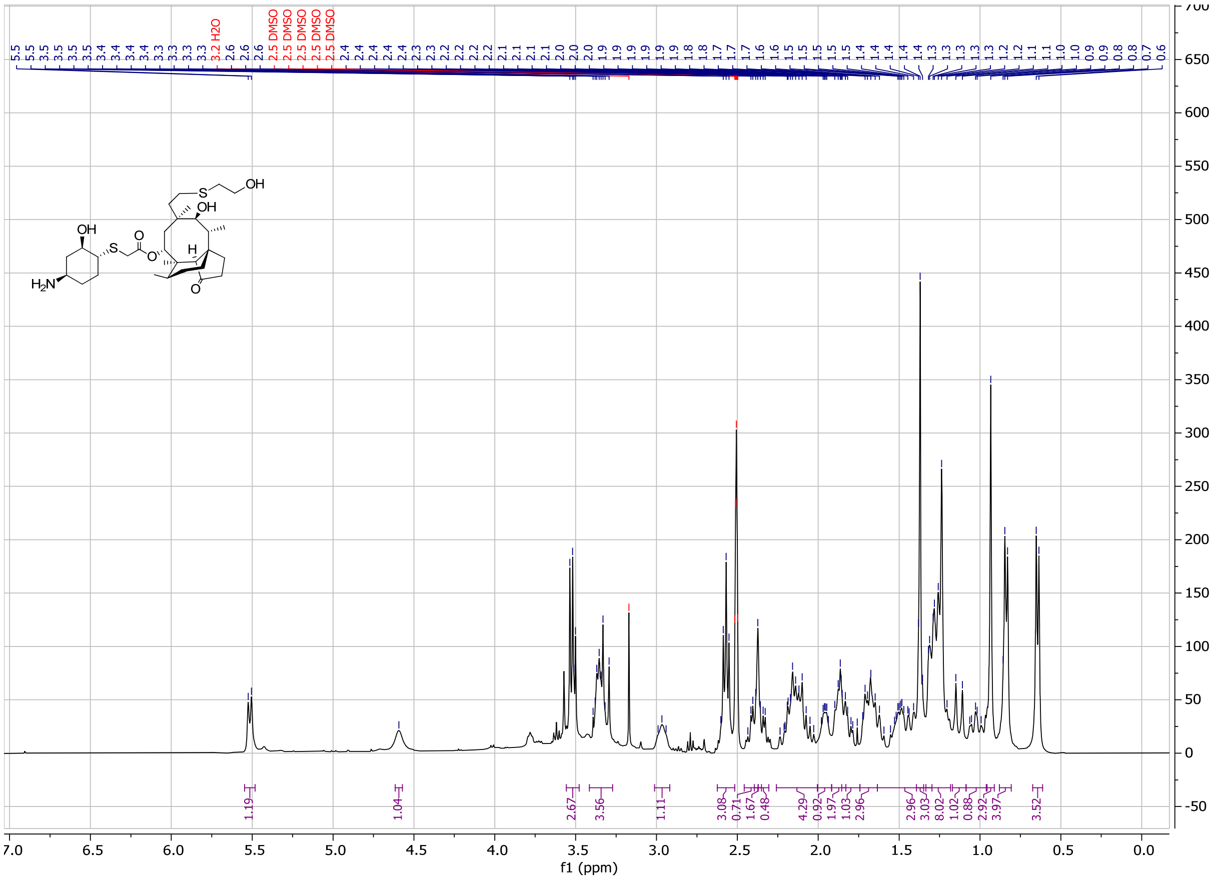

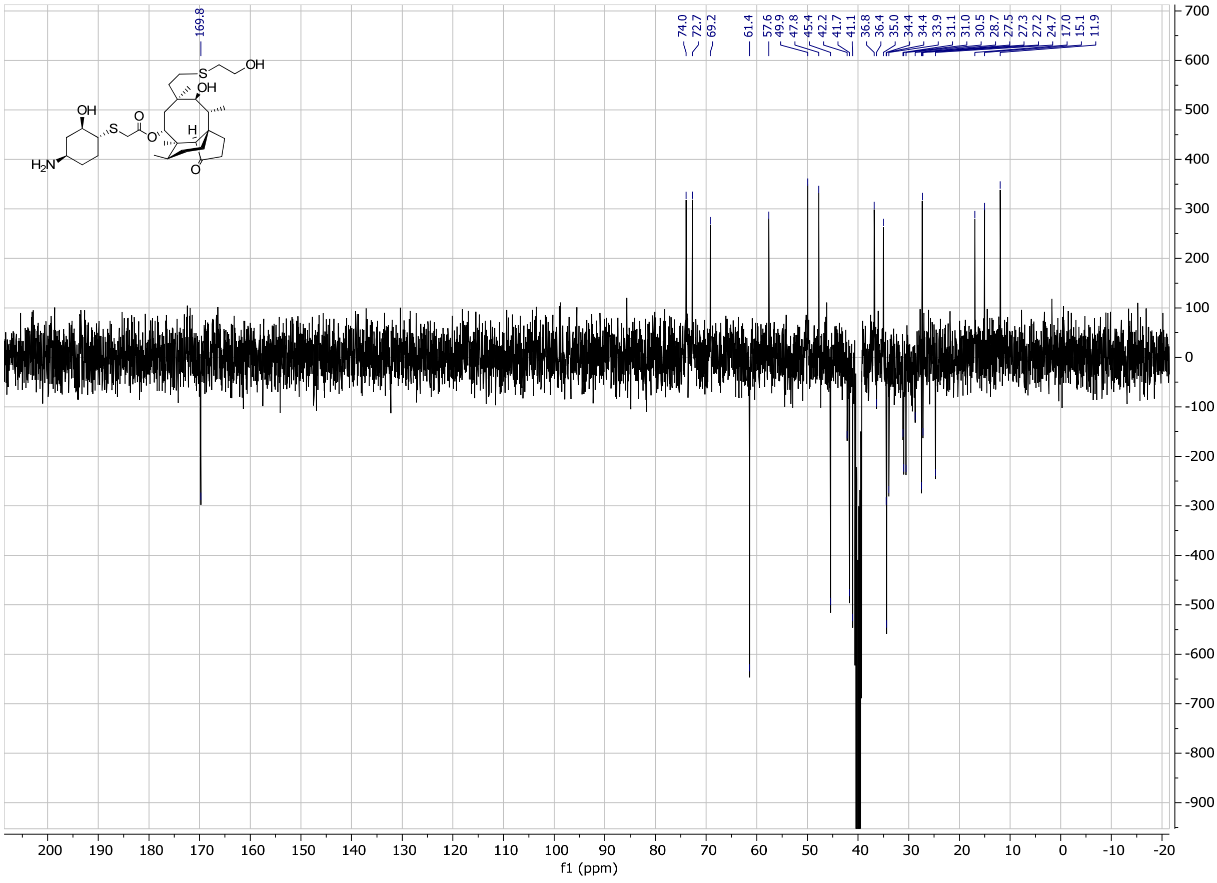

**Figure S25.** $^{1}H$ and $^{13}C$ NMR spectrum (400 MHz, DMSO) of compound **14j**.

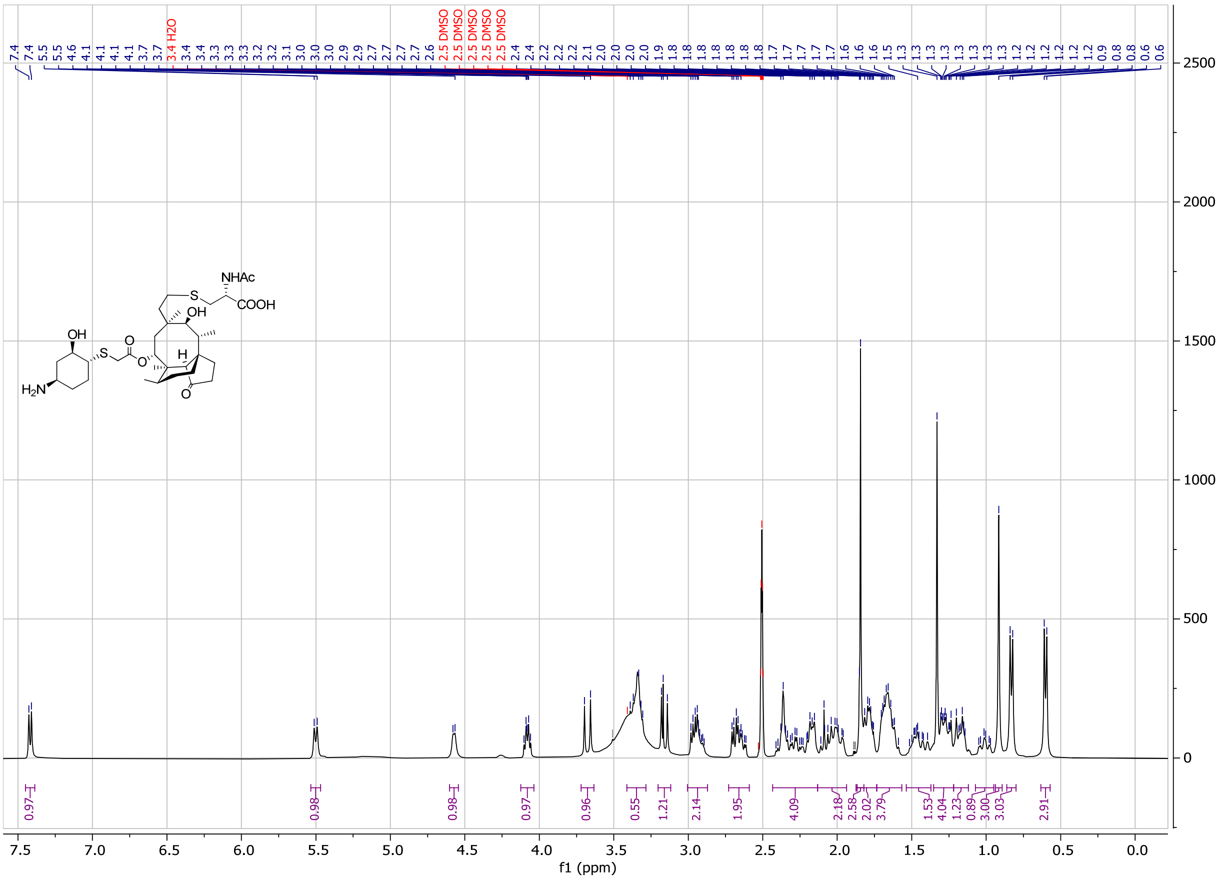

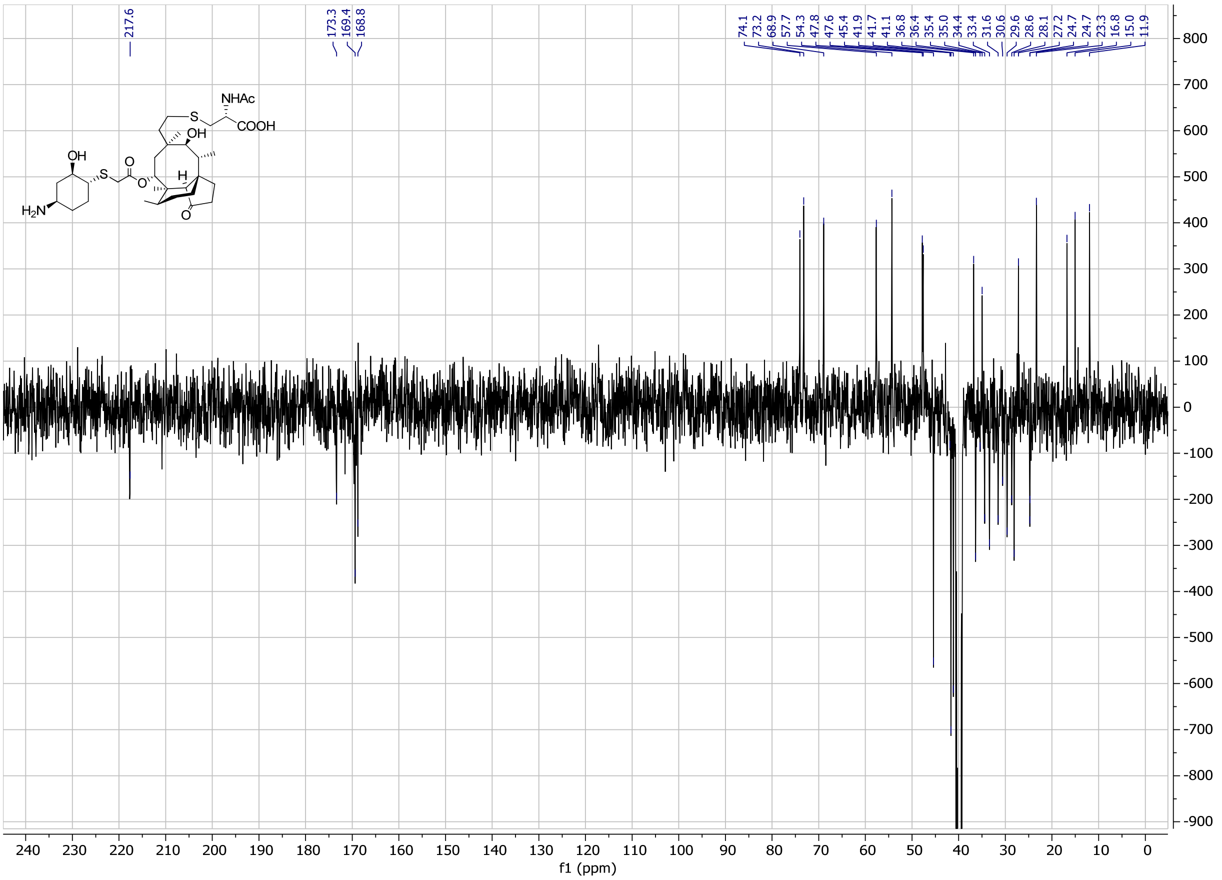

**Figure S26.** $^{1}H$ and $^{13}C$ NMR spectrum (400 MHz, DMSO) of compound **14k**.

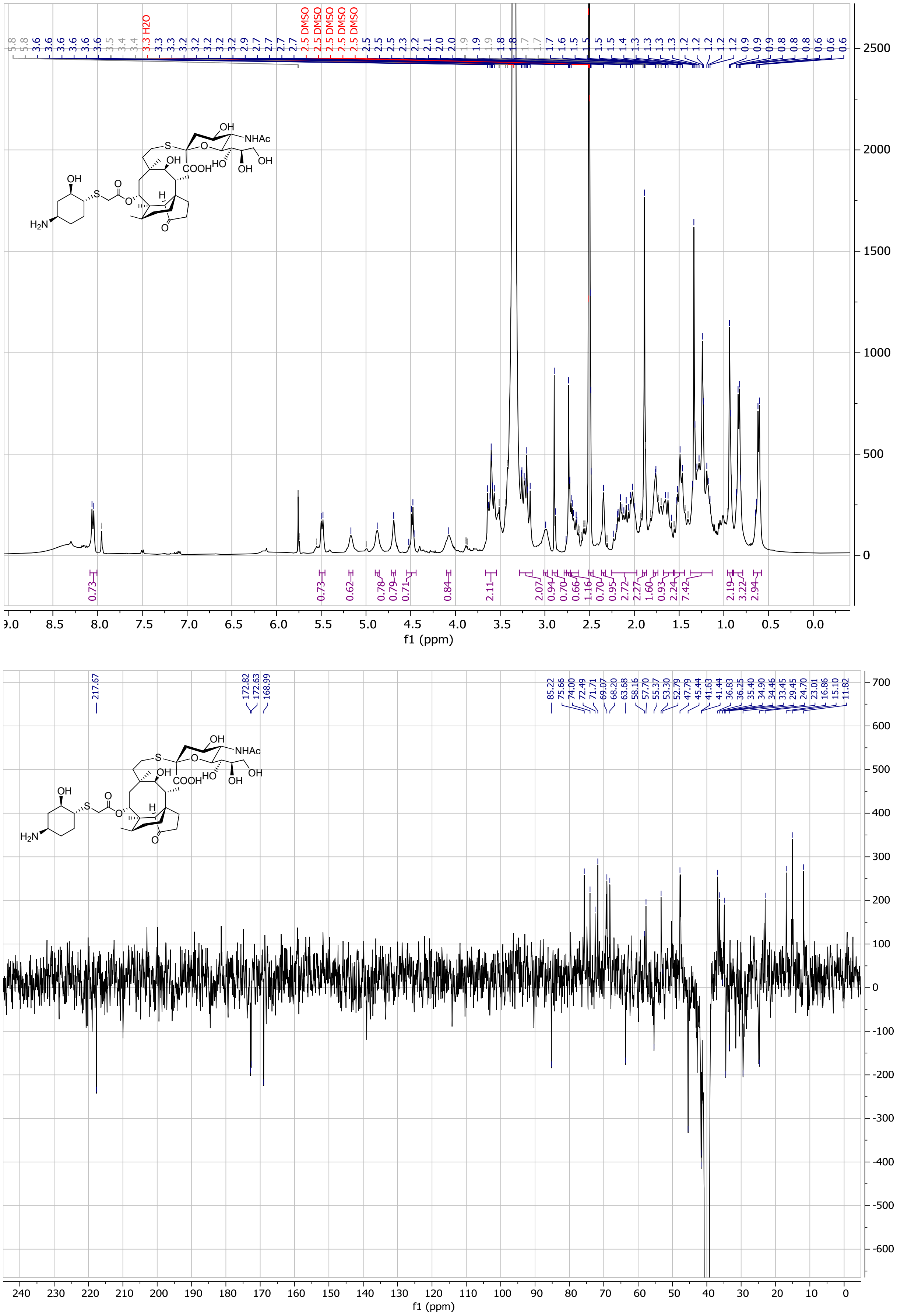


**Figure S27.** $^{1}H$ and $^{13}C$ NMR spectrum (400 MHz, DMSO) of compound **15h**.

For the low-temperature photoinitiated thiol-ene coupling reactions, the experimental set-up consists of the reaction vessel and the cooling medium (acetone–liquid nitrogen mixture) in a Dewar flask and a UV-lamp (Figure S1). Before irradiation, the entire set-up is covered by an aluminum foil tent to protect the laboratory personnel against UV light.

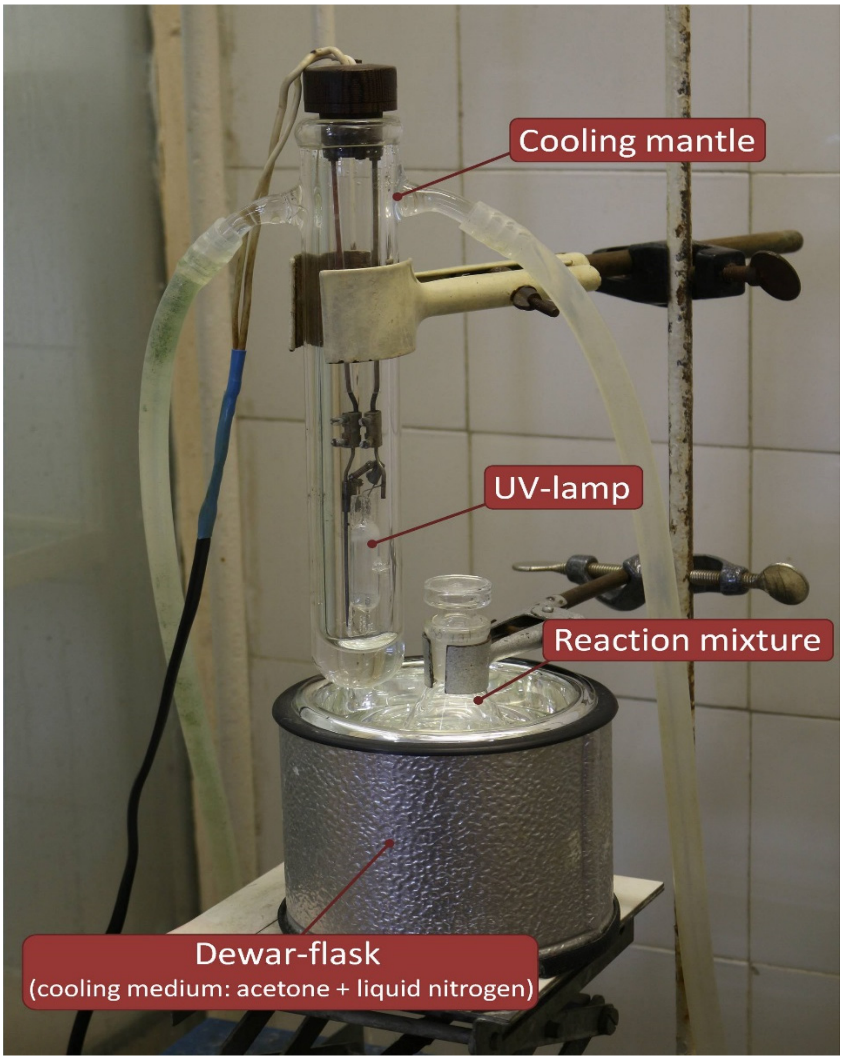


**Figure S28.** The experimental setup for carrying out hydrothiolation reactions at low temperature.

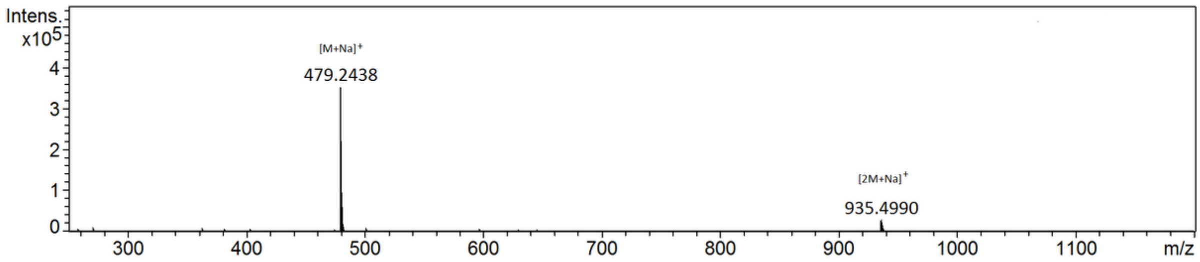


**Figure S29.** ESI-MS spectrum of compound **10j**.

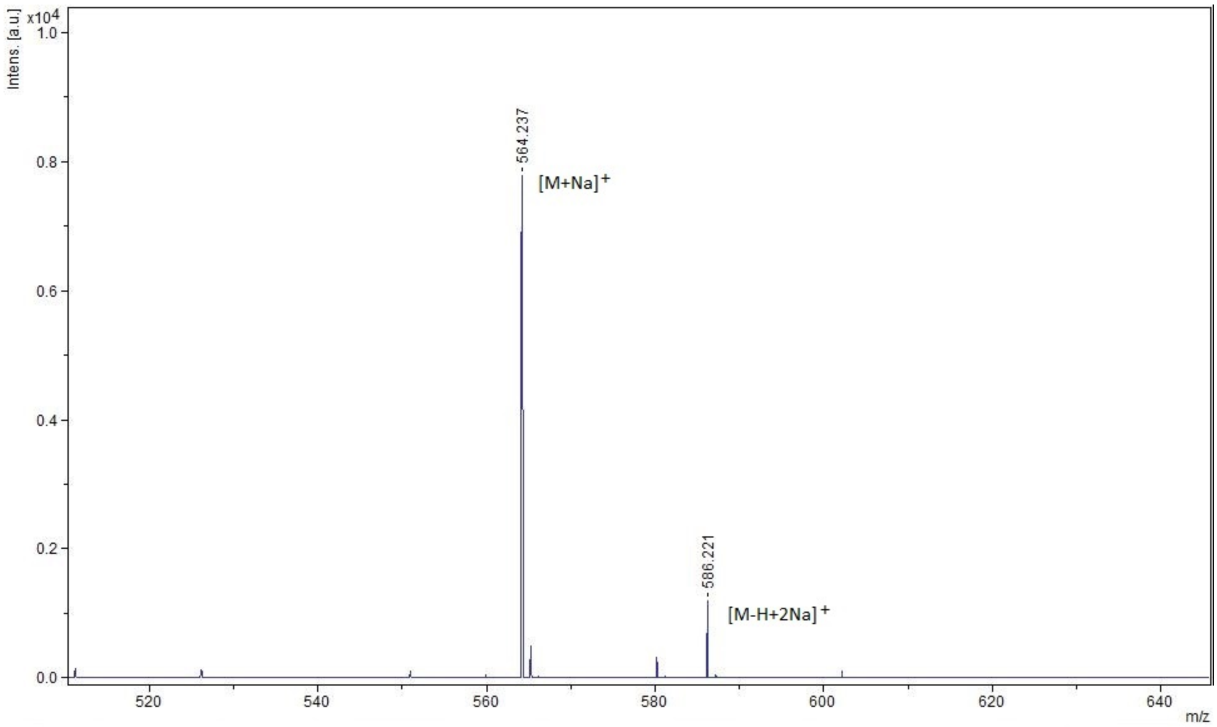


**Figure S30.** MALDI-MS spectrum of compound **10k**.

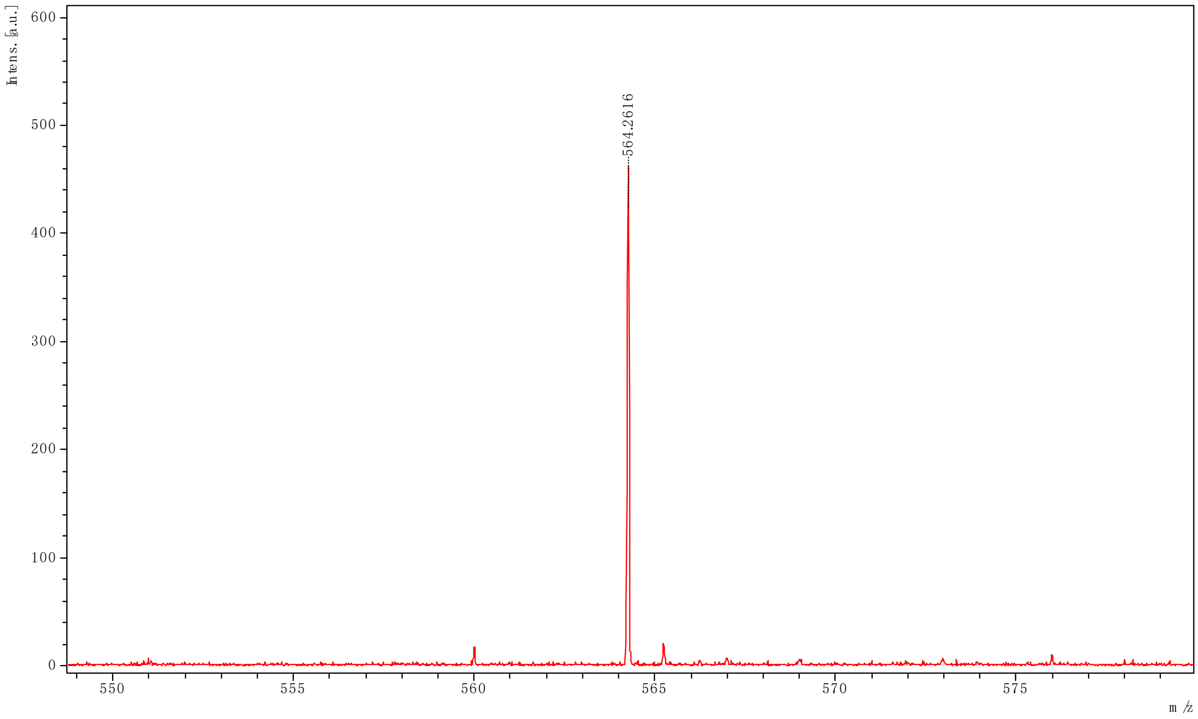


**Figure S31.** Calibrated MALDI-MS spectrum of compound **10k**.

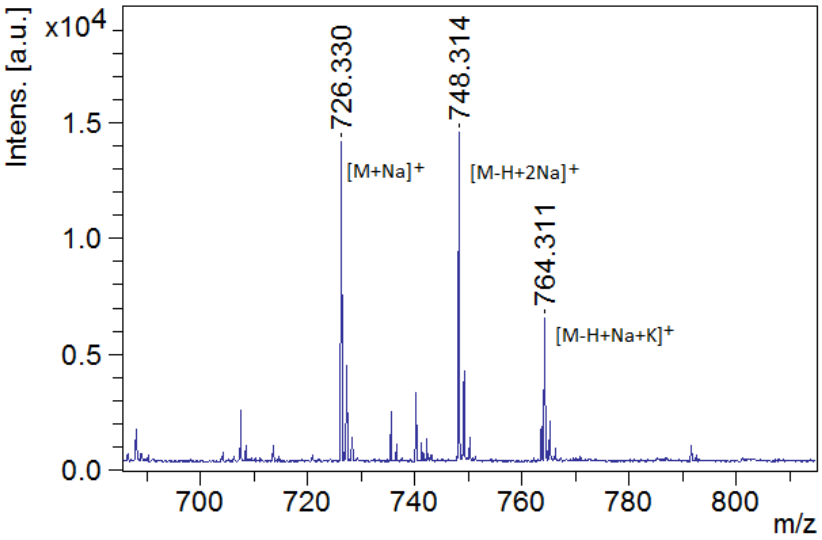


**Figure S32.** MALDI-MS spectrum of compound **11h**.

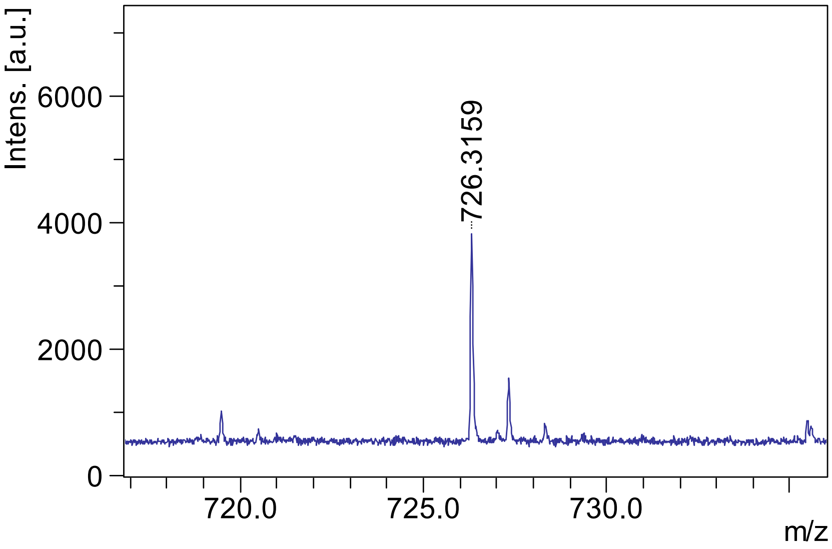


**Figure S33.** Calibrated MALDI-MS spectrum of compound **11h**.

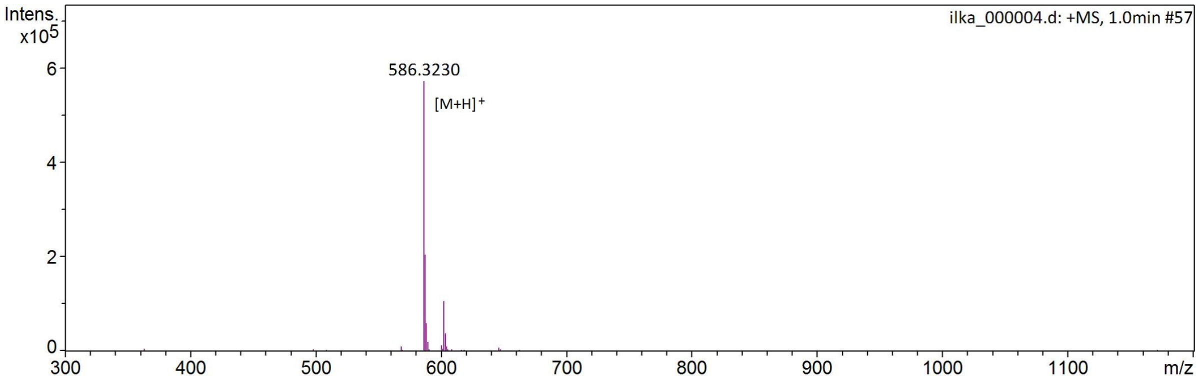


**Figure S34.** ESI-MS spectrum of compound **14j**.

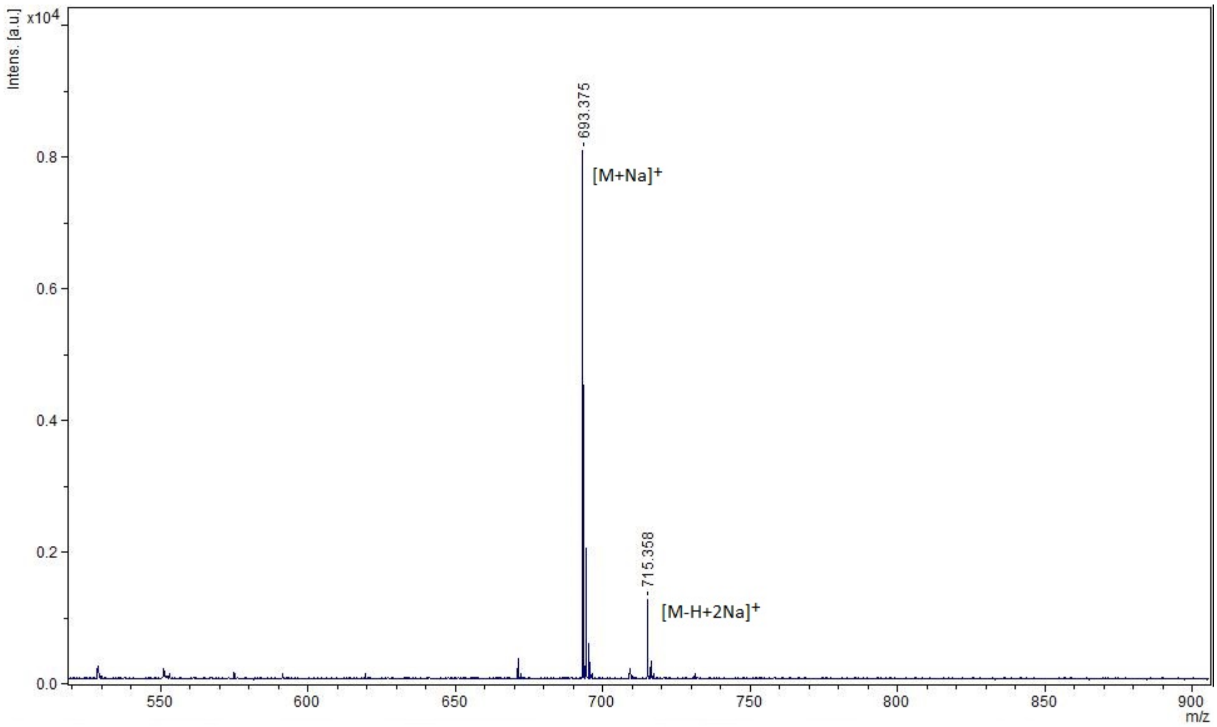


**Figure S35.** MALDI-MS spectrum of compound **14k**.

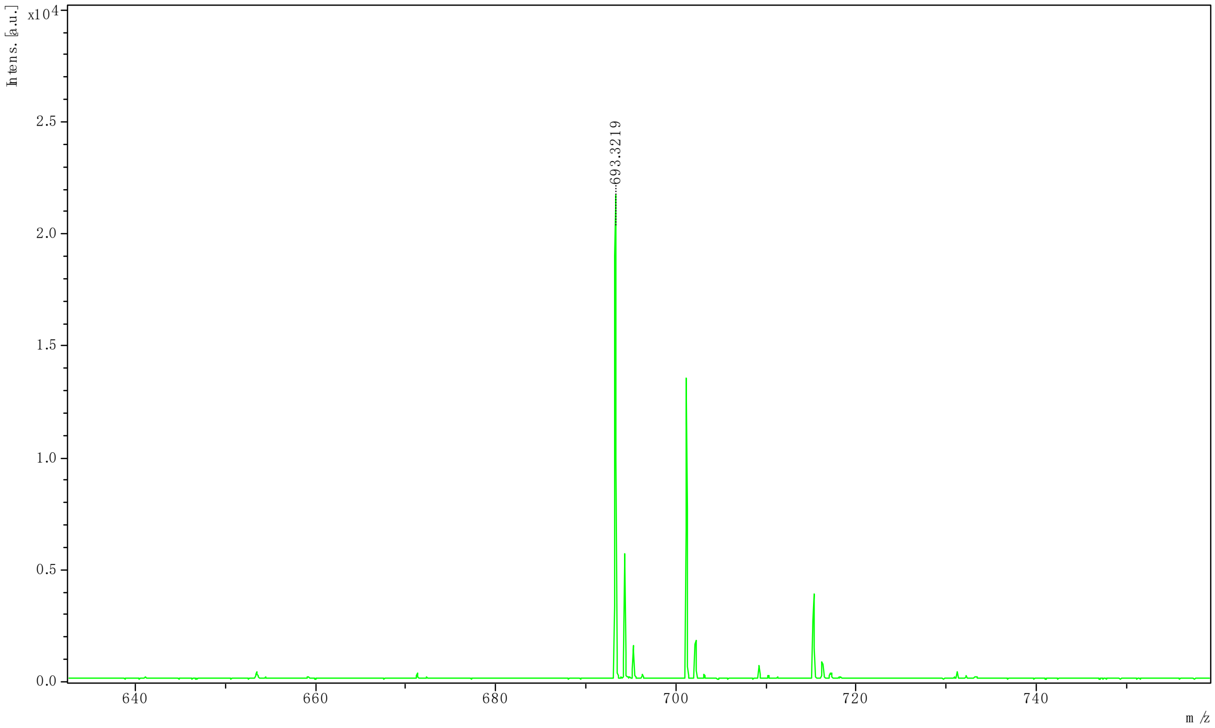


**Figure S36.** Calibrated MALDI-MS spectrum of compound **14k**.

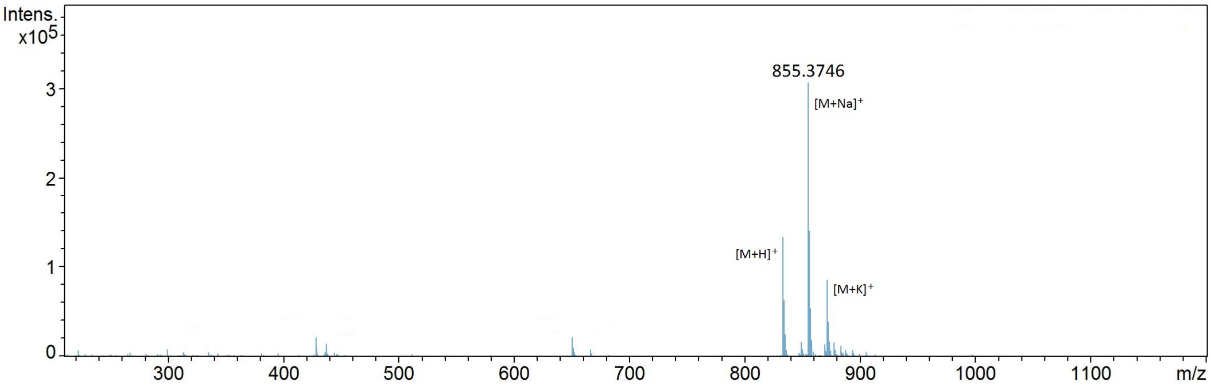


**Figure S37.** ESI-MS spectrum of compound **15h**.
